# Redundant ^15^N‑Mediated *J*‑Couplings Reveal an Aglycone Conformation in *N*‑Phenyl Glycosylamines

**DOI:** 10.1021/acs.joc.5c01892

**Published:** 2025-10-31

**Authors:** Nina Habanová, Jakub Kaminský, Kamil Parkan, Jakub Zýka, Vít Prouza, Blanka Klepetářová, Radek Pohl

**Affiliations:** † Institute of Organic Chemistry and Biochemistry, Czech Academy of Sciences, Flemingovo námĕstí 542/2, 166 10 Prague 6, Czech Republic; ‡ Department of Analytical Chemistry, 52735 University of Chemistry and Technology, Technická 5, 166 28 Prague, Czech Republic; § Department of Chemistry of Natural Compounds, 52735 University of Chemistry and Technology, Technická 5, 166 28 Prague, Czech Republic

## Abstract

Prediction of the conformation of novel *N*-glycomimetics is a crucial step in their rational design for drug discovery and glycobiology. We investigated the applicability of redundant ^15^N-mediated *J*-couplings for predicting the aniline aglycone conformation in ^15^N-labeled *N*-phenyl glycosylamines derived from d-glucose (d-Glc), d-mannose (d-Man), d-xylose (d-Xyl), and d-lyxose (d-Lyx). The compounds were prepared directly in an NMR tube by reacting free sugar with ^15^N-labeled aniline, achieving >90% conversion. The rate of *N*-phenyl glycosylamine formation depended on the concentration of an open-chain form of the sugar in the solution. In the closed form, the anilino substituent preferred equatorial orientation, which dictated the anomeric ratio and influenced the pyranose ring conformation, particularly in the case of d-Lyx and d-Xyl. The ^15^N label localized near the conformationally perturbed region provided redundant ^15^N-mediated *J*-couplings sensitive to the aniline aglycone conformation. By fitting the density functional theory (DFT)-calculated *J*-couplings of individual conformers to experimental data, we successfully predicted both the aniline aglycone conformation and the nitrogen atom configuration. Notably, the predicted major conformation of *N*-phenyl β-d-mannopyranosylamine (**6b**) corresponded to the conformer observed in the crystal structure.

## Graphical abstract

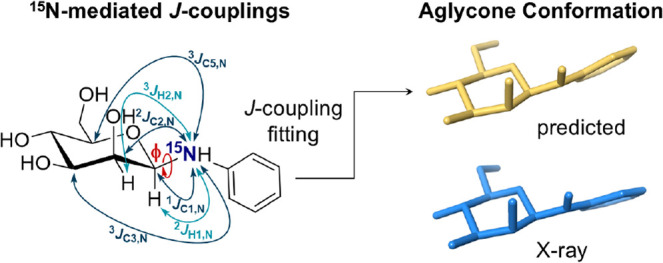

## Introduction

Glycomimetics are synthetic compounds that mimic the structure and function of natural carbohydrates. Structural modifications allow these molecules to achieve enhanced stability, affinity, and selectivity during lectin recognition.^1^ Since the recognition process is driven by the specific three-dimensional structure of a glycomimetic ligand, knowing the conformation in the free and bound states is crucial for designing effective drugs, studying carbohydrate-related biological processes, and optimizing the therapeutic potential of these compounds. The formation of a lectin/glycomimetic complex takes place in a solution where the inherent flexibility of sugar-like molecules often complicates the determination of glycomimetic conformation.^2^ This flexibility arises from conformational variability of pentose and hexose rings, rotations of hydroxyl and hydroxymethyl groups, and the orientation of substituents (such as aglycones or additional sugar moieties) attached to the anomeric carbon. The conformational flexibility is then manifested, for example, by binding of two distinct conformations of the mannobiose by concanavalin A.^3^ Additionally, lectins do not always bind the most populated conformer in the free state, as demonstrated in the case of the complex of galectin-1 with the 1,3-linked *C*-glycosyl derivative of lactose.^4^

Modified *N*-glycans and *N*-glycosides are the most prominent representatives of *N*-glycomimetics that hold considerable promise as therapeutic agents in diseases associated with aberrant carbohydrate recognition. Glycosylamines (often referred to as *N*-glycosides) are analogs of classical *O*-glycosides, differing in that a hemiaminal ether replaces the anomeric hemiacetal group. Despite the limited stability, several *N*-aryl or *N*-heteroaryl glycosylamines have been identified as antibiotic^5,6^ and anticancer^7^ compounds, or mechanistic probes of glycosidases.^8,9^

The most popular and frequently used method for conformational analysis of carbohydrate-like molecules in solution is nuclear magnetic resonance (NMR).^2,10,11^ Experimental NMR provides parameters such as chemical shifts, *J*-couplings, or NOEs, which correlate with the geometry of the conformers present in solution. However, the experimental spectra provide only conformer ensemble-averaged values of the NMR parameters. Therefore, combining NMR with molecular dynamics and calculating the NMR parameters is essential for decoding the geometry and population of individual conformers.

The present study builds upon the pioneering work of Serianni, who used redundant *J*-couplings (an ensemble of *J*-couplings sensitive to the same conformational element) mediated by ^13^C in the conformational analysis of ^13^C-enriched carbohydrates.^12^ He found that in addition to the well-known Karplus dependence of the vicinal (^3^ *J*) *J*-coupling on the dihedral angle of interacting nuclei, other *J*-couplings, such as direct (^1^ *J*), geminal (^2^ *J*) or long-range (^4^ *J*) ^13^C–^1^H or ^13^C–^13^C *J*-couplings provide useful conformational information.^13–16^ The analysis of redundant *J*-couplings has been recently upgraded by the *MA’AT* analysis that provides both conformational equilibria and dynamics of the studied system.^17^

X-ray crystallography was used in several cases to determine the conformation of *N*-phenyl glycosylamine derivatives in the solid state.^18–24^ In this study, we investigated the aglycone conformation of ^15^N-labeled *N*-phenyl glycosylamines in solution as model compounds for *N*-glycomimetics. The incorporation of a ^15^N label in the locality of conformational change gives rise to redundant ^15^N-mediated ^1^H–^15^N and ^13^C–^15^N *J*-couplings that are sensitive to the conformation and could be used as diagnostic signals. An example of such redundant *J*-couplings in ^15^N-labeled *N*-phenyl β-d-glucopyranosylamine is depicted in Figure 1. Such redundant *J*-couplings represent ensemble-averaged values of the individual conformers. The fitting of DFT-calculated *J*-couplings of individual conformers to experimental ones reveals the geometry and population of the conformers. Additionally, the method enables the prediction of the preferred nitrogen configuration. Since the nitrogen atom in aniline^25–27^ is pyramidal, it is chiral in *N*-phenyl glycosylamine derivatives.

**Figure 1:**
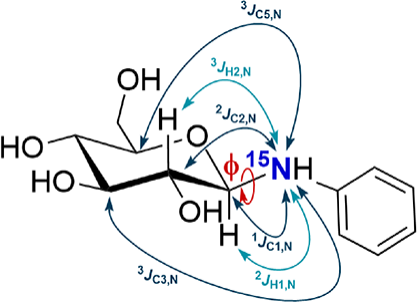
Redundant ^15^N-mediated *J*-couplings sensitive to ϕ changes in ^15^N-labeled *N*-phenyl β-d-glucopyranosylamine.

## Results and Discussion

### In Situ Preparation of ^15^N-Labeled *N*-Phenyl Glycosylamines

Glycosylamines are generally formed by the reaction of free sugar and an amine in an alcohol solvent, with or without acid catalysis. The formation of *N*-phenyl d-glucosylamine by this method has been known for more than 150 years.^28^ Although the reaction products can be isolated by crystallization, they, like other cyclic hemiaminals, are not stable and they undergo mutarotation and hydrolysis when dissolved in water.^29,30^ Recently, we reported a unified strategy for the synthesis of diglycosylamines using methanolic ammonia and demonstrated a novel approach to their *N*-acylation under acidic conditions in nitromethane, providing access to a new class of β-configured *N*-glycosidic derivatives.^31^ These studies highlight both the synthetic utility and the inherent lability of glycosylamines, which must be carefully considered when designing solution-state investigations.

We took advantage of glycosylamine stability in anhydrous methanol and prepared ^15^N-labeled *N*-phenyl glycosylamines (Scheme 1) by reacting a hexose (d-Glc and d-Man) or pentose (d-Xyl, d-Lyx) with ^15^N-aniline in methanol-*d*_4_ directly in the NMR tube. The selected hexoses and pentoses predominantly exist in solution in their pyranose forms but differ in their C-2 configuration, which could influence reactivity at the anomeric carbon and conformational preferences of the resulting *N*-phenyl glycosylamines.

**Scheme 1:**
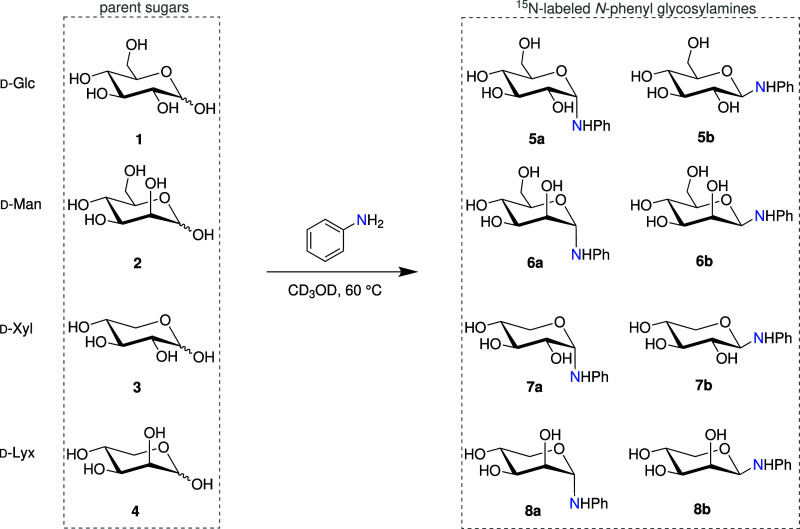
Structures of Investigated *N*-Phenyl ^15^N-Glycosylamines (**5–8**) and Their Parent Sugars (**1**–**4**)­^a^

a All structures are depicted in their ^4^ *C*_1_ pyranose form.

Reaction kinetics were monitored using ^1^H NMR spectroscopy for both uncatalyzed and acid-catalyzed (0.1 equiv of acetic acid-*d*_4_) conditions (Figures S1–S5, Supporting Information). The acid catalysis was found to significantly accelerate the reaction, as confirmed particularly for the pentose substrates (Table S1, Supporting Information). The relative rates of *N*-phenyl glycosylamine formation decreased in the order d-Lyx > d-Xyl > d-Man > d-Glc, which correlates with the equilibrium concentrations of the open-chain forms of the respective sugars.^32,33^ This observation is mechanistically consistent with the formation of a Schiff base intermediate (Scheme 2), which requires access to the aldehyde form of the sugar. Therefore, it is important for the kinetics monitoring (Figure S5, Supporting Information) to allow mutarotation of the starting sugar to reach equilibrium (Table S2, Supporting Information) before the addition of ^15^N-aniline. This was done by dissolving the starting sugar in dry methanol-*d*_4_ and heating the solution at 60 °C for 1 h (Figure 2a,b). The equilibrium anomeric ratio of the parent sugar in the presence or absence of 0.1 equiv of acetic acid-*d*_4_ is shown in Table S2, Supporting Information.

**Scheme 2:**
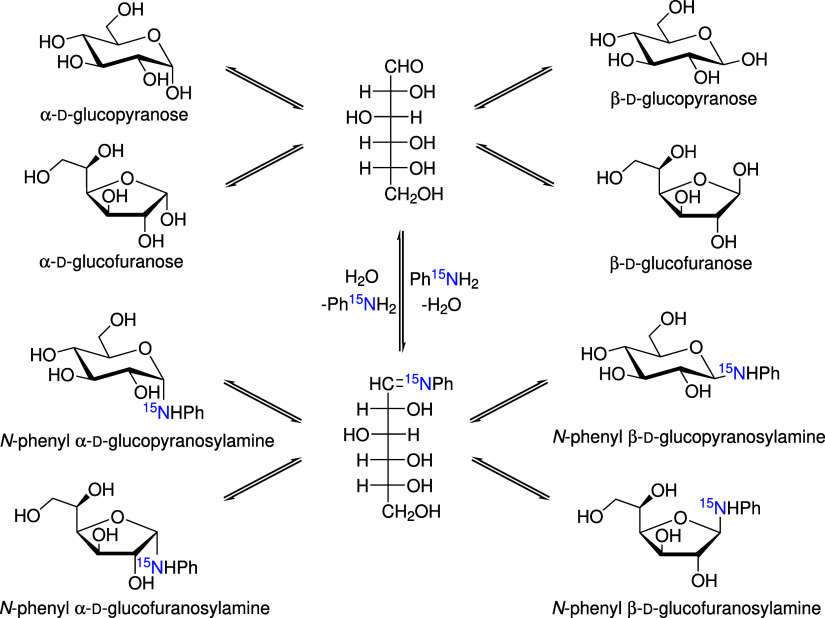
Possible Compounds Existing in the Equilibrium Mixture when Reacting d-Glucose with ^15^N-Aniline

**Figure 2:**
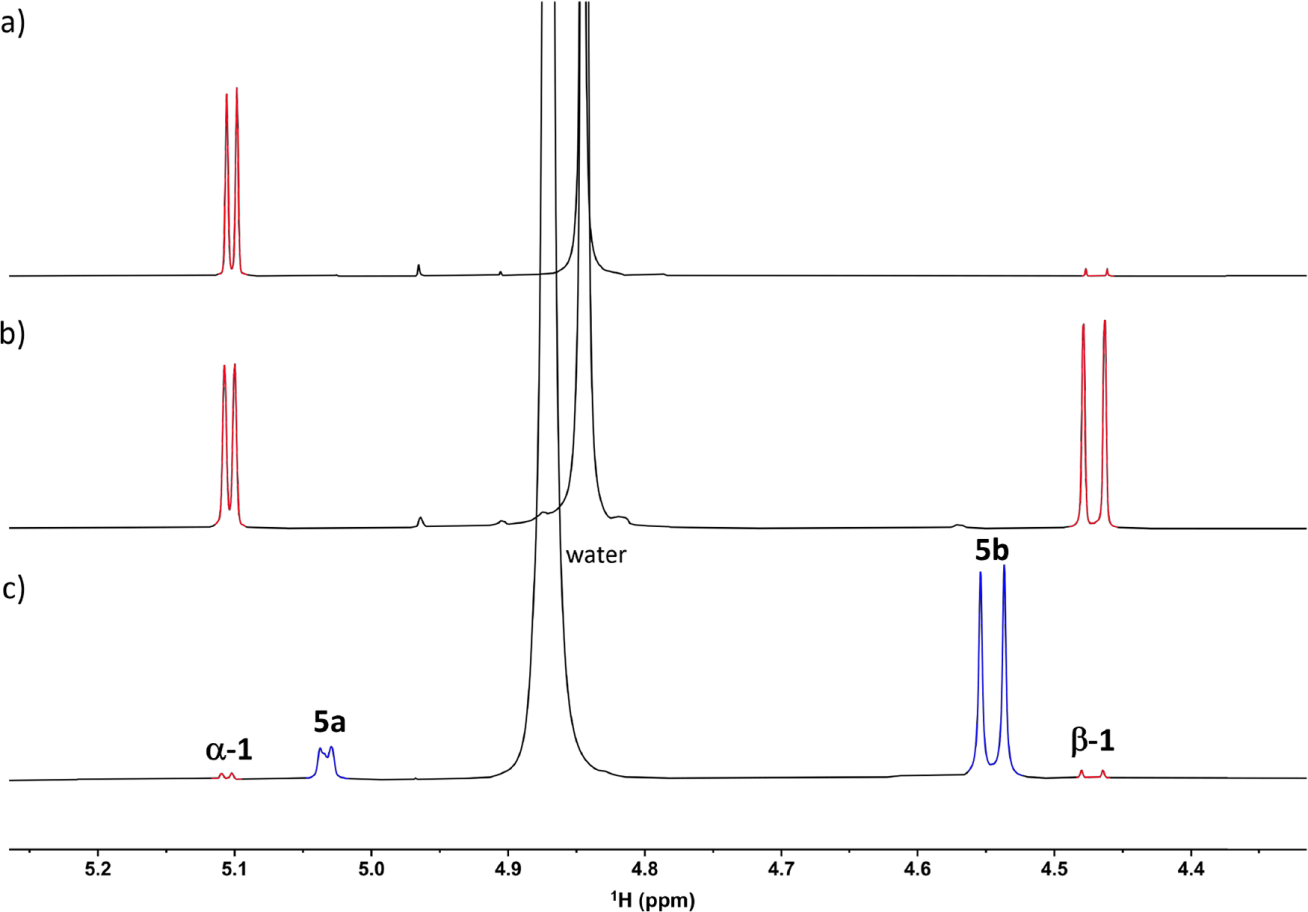
Expansion of ^1^H NMR spectra showing anomeric protons of (a) freshly dissolved **1** (in red) in methanol-*d*_4_; (b) **1** (in red) in methanol-*d*_4_ after mutarotation; (c) **5a** and **5b** (in blue) in the reaction mixture after reaction of **1** (in red) with ^15^N-aniline with marked anomeric protons.

The final composition of the reaction mixture follows the equilibrium depicted in Scheme 2 for d-glucose, and can be influenced by the solvent, temperature, pH, and ratio of the starting sugar and aniline.^34,35^ In our experimental setup (1.3 eq of ^15^N-aniline, 60 °C, catalytic amount of acetic acid-*d*_4_ in dry methanol-*d*_4_, sealed NMR tube), we detected both anomers of *N*-phenyl ^15^N-glycopyranosylamines in the ratios shown in Table 1. The conversion of the starting sugars was ≥ 90%, and Figure 2c illustrates the final composition of the reaction mixture after the reaction of d-glucose with ^15^N-aniline. The anomeric ratio was determined by integration of the anomeric protons H-1 or *o*-protons of the phenyl moiety in the ^1^H NMR spectra.

**Table 1:** Characteristics of the In Situ Acid-Catalyzed Reaction of Sugars with ^15^N-Aniline Yielding an Anomeric Mixture of ^15^N-Labeled *N*-Phenyl d-Glycopyranosylamines

parent sugar	time to equilibrium (hours)	*k* (s^–1^)	conversion (%)	anomeric mixture (%)	
d-Glc **1**	36	3.1 × 10^–5^	90	**5a**:**5b**	18:82
d-Man **2**	8	1.6 × 10^–4^	93	**6a**:**6b**	25:75
d-Xyl **3**	6	2.2 × 10^–4^	96	**7a** ^a^:**7b**	36:64
d-Lyx **4**	5	2.5 × 10^–4^	96	**8a** ^a^:**8b** ^a^	50:50

a ^4^ *C*_1_ ⇌ ^1^ *C*_4_ equilibrium.

### Structure of *N*-Phenyl ^15^N-Glycosylamines

The main goal of this study was to utilize ^15^N labeling for the NMR-based conformational analysis of the aniline aglycone. It was therefore essential to be certain of (1) whether the labeling was successful, (2) in which cyclic form (pyranose/furanose) products exist, (3) the sugar ring conformation, and (4) the configuration at the anomeric carbon. Careful analysis of the NMR spectra can answer all of these queries. We assigned all ^1^H and ^13^C resonances using the combination of H,H-COSY, H,H-ROESY, and H,C-HSQC techniques. Briefly, the assignment started with downfield anomeric signals in ^1^H and ^13^C NMR spectra. Proton resonances were then assigned using H,H-COSY, and carbon resonances using H,H-HSQC. Anomeric configuration was decided by inspection of vicinal *J*-couplings of ring protons. In case of an ambiguity (**6a** and **6b**), H,H-ROESY spectra were supportive. To read the value of ^3^ *J*(NH,H1), we switched the solvent CD_3_OD for CD_3_OH. The complete assignment of the ^1^H, ^13^C, and ^15^N resonances and *J*-coupling values is available in Tables S3 and S4 in the Supporting Information.

The incorporation of ^15^N isotope was evident by the splitting of C-1, C-2, C-3 and C-5 signals in ^1^H-decoupled ^13^C NMR spectra due to ^15^N–^13^C scalar spin–spin interaction (Table 2). Furthermore, the splitting of C-5 by ^15^N confirmed the pyranose form in all of the prepared ^15^N-labeled *N*-phenyl glycosylamines. An example of ^13^C APT NMR spectrum for *N*-phenyl α- (**6a**) and β-d-mannopyranosylamine (**6b**) is shown in Figure 3. Other ^13^C spectra with signal assignment for all studied ^15^N-labeled compounds are presented in Figures S7, S11, S15, and S19 in the Supporting Information.

**Figure 3:**
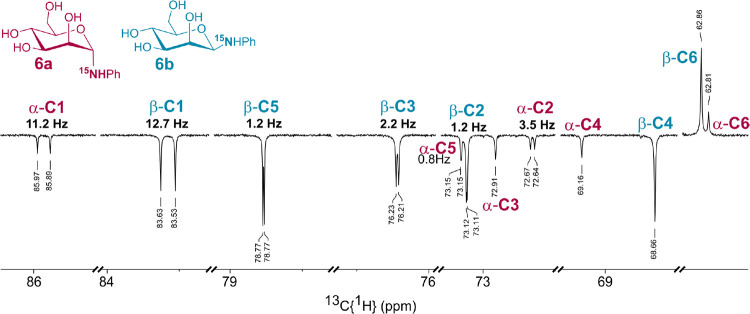
Expansion of ^13^C APT NMR spectrum of **6a** and **6b** showing splitting of mannose ^13^C signals by ^15^N nucleus.

**Table 2:** Experimental ^1^H–^1^H, ^15^N–^1^H, and ^15^N–^13^C *J*-Couplings of ^15^N-Labeled *N*-Phenyl Glycosylamines in Methanol-*d*_4_ ^a^

*J*-coupling [Hz]	**5a**	**5b**	**6a**	**6b**	**7a**	**7b**	**8a**	**8b**
^3^ *J*(H1,H2)	4.2	8.7	1.8	1.0	3.6	8.5	7.6	2.0
^3^ *J*(H2,H3)	nd	8.7	3.4	3.2	6.8	8.7	3.2	3.4
^3^ *J*(H3,H4)	nd	8.7	9.3	9.3	6.8	8.9	4.8	8.3
^3^ *J*(H4,H5)	nd	9.6	nd	9.3	4.0	5.3	2.2	4.7
^3^ *J*(H4,H5′)					7.1	10.4	3.3	8.7
^3^ *J*(H5,H6)	3.2	2.3	4.7	2.5				
^3^ *J*(H5,H6′)	4.2	5.2	3.0	5.5				
^2^ *J*(H5,H5′)					11.7	11.3	12.1	11.5
^2^ *J*(H6,H6′)	11.9	12.0	11.7	11.9				
^4^ *J*(H3,H5)	0	0	0	0	0	0	1.0	0
^3^ *J*(NH,H1)	3.4	8.5	4.6	9.8	5.0	8.6	7.7	nd
^3^ *J*(^15^N,H2)	nd	2.1	0.5	1.0	1.8	2.0	1.7	1.3
^1^ *J*(^15^N,C1)	10.9	13.1	11.2	12.7	11.8	13.1	12.5	12.1
^2^ *J*(^15^N,C2)	1.3	1.3	3.5	0.9	1.2	1.2	1.7	b
^3^ *J*(^15^N,C3)	0	2.3	0	2.2	b	2.3	1.3	b
^3^ *J*(^15^N,C5)	1.1	1.4	0.8	1.2	b	1.6	1.0	b
^1^ *J*(^15^N,C*i*)	13.0	13.7	13.6	13.9	13.7	13.7	13.7	14.0
^2^ *J*(^15^N,C*o*)	2.3	2.3	2.3	2.1	2.3	2.3	2.3	2.3
^3^ *J*(^15^N,C*m*)	1.4	1.4	1.4	1.3	1.3	1.3	1.4	1.4

a b: broad signal; nd: not determined due to the signal overlap.

#### Sugar Ring Conformation

The sugar ring conformation in the pyranose form is traditionally determined by the evaluation of ^3^ *J*(H,H) of ring protons.^36^ We focused our attention on ^3^ *J*(H3,H4) and ^3^ *J*(H4,H5) couplings that indicate ^4^ *C*_1_ or ^1^ *C*_4_ ring conformation or an equilibrium mixture of these two states. Large values (8.8–10.4 Hz)^36^ of ^3^ *J*(H3,H4) and ^3^ *J*(H4,H5) suggesting ^4^ *C*_1_ conformation due to the axial–axial interaction were observed for glucosylamine **5b** and mannosylamines **6a** and **6b**. Extensive overlap of the H-2,3,4 and 5 signals in the ^1^H NMR spectrum of **5a** made reading of the *J*-couplings difficult, but we expected the ^4^ *C*_1_ conformation as for other known α-d-glucopyranoside derivatives. A somewhat different situation occurred in the case of pentopyranoses **7a**, **8a**, and **8b**. Exceptional is **7b**, where the ^4^ *C*_1_ conformation with H-3 and H-4 in the axial position was evident, the other *N*-phenyl pentopyranosylamines exist in the CD_3_OD solution as an equilibrium mixture of ^4^ *C*_1_ and ^1^ *C*_4_ conformers as follows from unexpected values of ^3^ *J*(H3,H4), ^3^ *J*(H4,H5) and ^3^ *J*(H4,H5′) in Table 2. A similar observation was recently reported for d-xylopyranosylamines and amides.^37^ The ratio of the conformers was estimated by fitting the theoretical (calculated by DFT or Altona-Karplus equation^38^) *J*-couplings of the ring protons to the experimental by the linear combination of the theoretical values of both conformations (Tables S5–S12, Supporting Information). *J*-coupling analysis revealed that **8a**, primarily by 86%, adopts the ^1^ *C*_4_ conformation in a CD_3_OD solution. When we acquired ^13^C NMR we noticed significant broadening of C-2 and C-5 in **8b** suggesting intermediate chemical exchange between ^4^ *C*_1_ and ^1^ *C*_4_ conformers. Low temperature measurements at −90 °C resulted in the splitting of the ^13^C signals into two resolved sets of resonances, thus providing experimental evidence of the two conformers of **8b** (Figure S22, Supporting Information). We also analyzed the conformation of starting pentopyranoses and found, in agreement with the literature,^39^ that d-lyxopyranose **4** exists in solution as ^4^ *C*_1_/^1^ *C*_4_ conformational equilibrium (Tables S11 and S12, Supporting Information).

#### Anomeric Effect

We found that the aniline substituent in *N*-phenyl glycosylamines prefers the equatorial orientation in all investigated *N*-phenyl glycosylamines (Table 1). This observation is obvious for d-glucosyl, d-mannosyl, and d-xylosyl derivatives, in which β-anomers prevail. In the case of d-lyxosylamine **8**, we observed a 1:1 mixture of α- and β-anomers, however, the α-anomer occurs predominantly in the ^1^ *C*_4_ conformation with an aniline substituent in the equatorial arrangement. The prevalence of the equatorial orientation of the aglycone can be explained by so-called reverse anomeric effect, which counteracts the classical anomeric effect.^40,41^ This phenomenon has been reported for various glycosides bearing an ammonium or phosphonium group at C-1, certain sulfonium derivatives, or glycosyl oxocarbenium ions.^42–46^ Currently, the reverse anomeric effect is considered to result from a complex of multiple, often opposing factors, including electrostatic repulsion, lone pair (dipole–dipole) interactions, hyperconjugation, and steric effects, rather than a single dominant interaction.^47–50^ In the case of classical anomeric effect, the electrostatic repulsion usually explains the destabilization of the equatorial orientation due to the electrostatic repulsion between the electron-rich ring oxygen O-5 and the substituent at C-1. On the contrary, in glycosides with ammonium or phosphonium groups at C-1, attraction between the lone pairs of the ring oxygen and positively charged substituents favors the equatorial orientation. Hyperconjugation is a stabilizing interaction involving the *n*_X_ → σ* delocalization between antibonding σ orbitals and lone pair orbitals of oxygen or nitrogen.^51^ According to recent high-level DFT and ab initio calculations, hyperconjugation usually plays a minor role.^52–54^ To determine which effect plays a decisive role in the case of compounds **5** and **6**, we performed a series of DFT calculations for all their minima obtained by systematic conformational scanning (Figure S33, Supporting Information).

For each obtained conformer, we calculated the total hyperconjugation energy, including the delocalization of a lone pair from a donor orbital *i* (here, the lone pair at O-5 or N-1) to an acceptor orbital *j* (σ_*C*1–*N*1_^*^ or σ_*O*5–*C*1_^*^), as well as the dipole moment. Then, the properties were Boltzmann averaged for the α- and β-anomers according to B3LYP/aug-cc-pVTZ Gibbs energies (see Methods for details). Boltzmann averaging of the hyperconjugation effect or dipole moment was performed within the conformers of a particular anomer. Only for the calculation of the population of individual anomers were the conformers of both anomers combined. Table S13 in Supporting Information summarizes the calculated properties for all studied conformers of **5** and **6**, as well as the average values for the α- and β-anomers. Note that the overall molecular dipole moment can be both an indicator of the electrostatic environment and a consequence of charge distribution. Therefore, higher dipole moments often correlate with higher destabilization of axial conformers owing to more localized charge distribution.

The table shows that, while for **5** the average stabilization by hyperconjugation is higher for β-anomers than for α-anomers, the opposite is true for **6**. For both compounds **5** and **6**, we observed higher stabilization of the α-anomer due to delocalization of the lone pair on O-5 to the C1–N1 bond. The apparent disadvantage of the β-anomer is compensated by the higher stabilization due to the delocalization of the lone pair on N-1 to the C1–O5 bond. Compound **5a** has a higher dipole moment than **5b**, which in turn favors the β-anomer compared to the α-anomer due to lower electrostatic repulsion. For **6a** and **6b**, however, the trend is reversed. Thus, the higher stability of the β-anomer in compound **5** appears to be a consequence of more favorable hyperconjugation and less electrostatic repulsion. On the other hand, effects other than those discussed here must contribute to the observed stability of compound **6**. It is important to note that the effects studied vary across individual conformers and the resulting average values for anomers depend on the quality of the predictions of conformational energies, and hence populations. This is particularly evident in the dipole moment of **5**, where the average value for **5a** is unexpectedly higher than for **5b**. This discrepancy likely results from an overestimation of the DFT population of the (*R*)-(+*gauche*) conformer of compound **5a**, which has a substantially higher calculated dipole moment compared to the other conformers. However, the total anomeric populations predicted by Boltzmann weighting roughly match the experimental ratios. In conclusion, other influences such as the solvent effect, intramolecular hydrogen bonding, temperature and entropic effects may also affect the stability of individual anomers.^55^ For example, we observed a slight stabilization of **6b** compared to **6a** by 0.1 kcal/mol due to entropy.

### Conformational Analysis of Aniline Aglycone

Three staggered conformations are considered when discussing the plausible orientations of an aglycone in glycosides. The orientation of the aniline aglycone in the *N*-phenyl d-glycopyranosylamines presented here is defined by the dihedral angle ϕ between the O5–C1–N–C*i* atoms, providing three conformers: –*gauche*, +*gauche*, and *trans*. The overall conformation of the aniline aglycone, however, is more complex. The geometry on the nitrogen atom in substituted anilines is a compromise between *sp* ^3^ hybridization, observed for aliphatic amines, and conjugation of the nitrogen lone electron pair with the aromatic system. This results in a shallow nitrogen pyramidization, and its extent depends on the substitution of the aromatic system and on supramolecular interactions.^27^ In *N*-phenyl glycosylamines, therefore, the pyramidal arrangement of substituents on nitrogen introduces an additional element of chirality, resulting in two nitrogen configurations, *R* and *S*, for each conformer (Figure 4).

**Figure 4:**
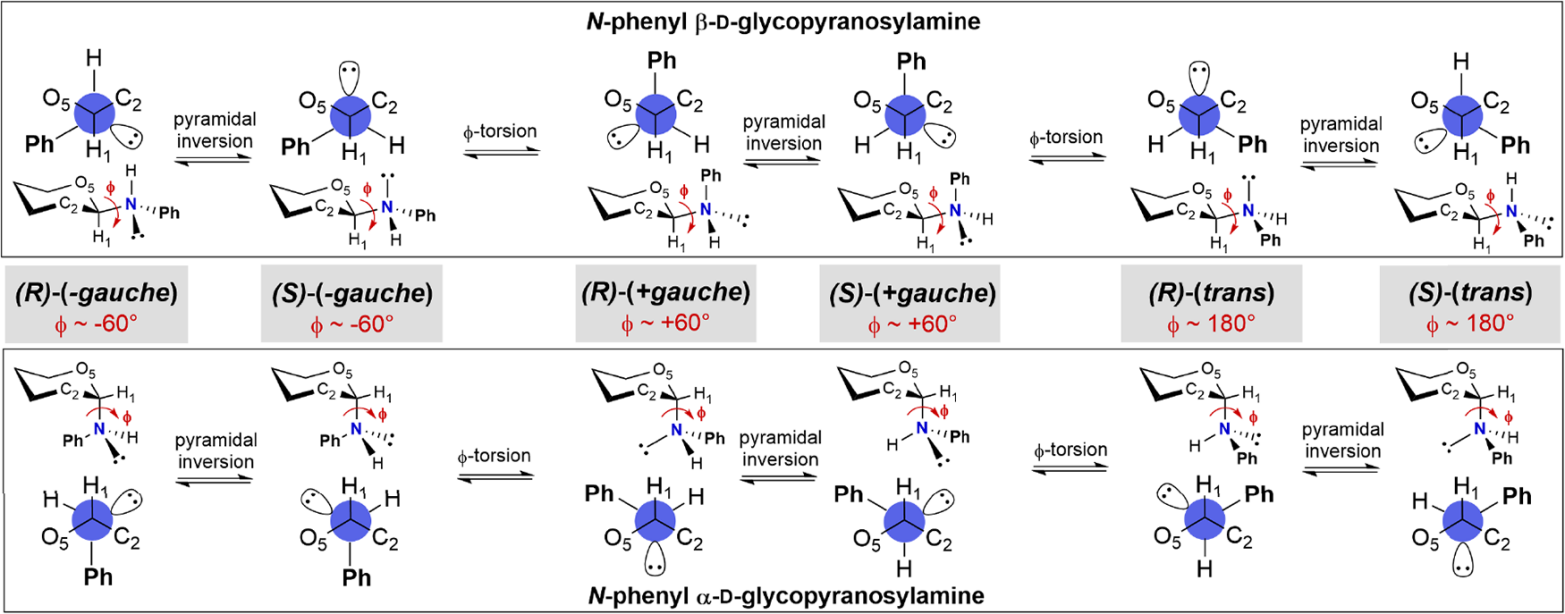
Aglycone conformation in *N*-phenyl d-glycopyranosylamines (ϕ = O5–C1–N–C*i*).

The factors driving the preferred orientation of the aglycone include orbital interactions known as the *exo*-anomeric effect,^50^ hydrogen bonding and steric repulsions. The aglycone conformation of a few substituted *N*-phenyl glycosylamines has already been studied in the solid state using X-ray crystallography.^18–20^ Although these results provide valuable insight into *N*-phenyl glycosylamines structure, they do not reflect the flexibility and dynamics of molecules in solution, such as fast pyramidal inversion of nitrogen configuration,^56^ a subtle balance between hybridization and conjugation in substituted anilines,^27^ and rotation around the glycosidic bond. The work presented here, therefore, sheds light on the conformational preferences of the aniline aglycone in *N*-phenyl glycosylamines in solution using a combination of molecular modeling, DFT calculations of NMR parameters, particularly *J*-couplings, and experimental NMR.

For subsequent conformational analysis of the aniline aglycone, we selected *N*-phenyl glycosylamines **5a**, **5b**, **6a**, **6b**, and **7b** with a stable ring conformation, where more than 90% of the pyranose ring exists in the ^4^ *C*_1_ conformation.

#### Well-Tempered Metadynamics Simulations

We performed well-tempered metadynamics (WTMtD) simulations in the Desmond program using the OPLS4 force field to gain insight into the dynamics and preferred conformations of the studied systems. The dihedral angle ϕ was selected as the collective variable and the bias factor (kTemp) was set to 2.4 kcal/mol, assuming an energy barrier of approximately 4 kcal/mol for the interconversion between individual conformers. The results for WTMtD are presented in Figure 5, which shows the dependence of the relative free energy on the dihedral angle ϕ.

**Figure 5:**
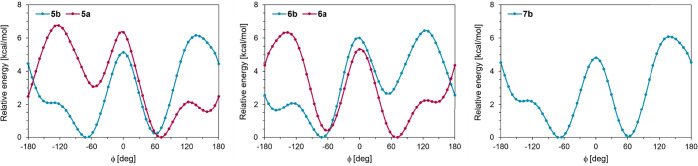
Relative energies of compounds as a function of ϕ extracted from well-tempered metadynamics simulations.

As expected, WTMtD provided the three energy minima corresponding to the –*gauche*, +*gauche*, and *trans* conformers; however, their populations differ for compounds with the opposite anomeric configurations (**5a** vs **5b**, or **6a** vs **6b**) and for compounds with opposite configurations at C-2 (**5a** vs **6a**, or **5b** vs **6b**). The free energy profiles are almost identical for *N*-phenyl β-d-glycosylamines **5b** and **7b** with the same both anomeric and C-2 configurations. In such cases, –*gauche* and +*gauche* conformers are almost equally populated with a slight excess of the –*gauche* conformation. When the C-2 configuration changes as in *N*-phenyl β-d-mannopyranosylamine **6b**, the –*gauche* conformer becomes major, followed by the *trans* conformer and the least populated +*gauche* conformer. In general, WTMtD showed that the –*gauche* conformation is preferred by *N*-phenyl β-d-glycosylamines, whereas *N*-phenyl α-d-glycosylamines favor the +*gauche* conformation. Furthermore, the analysis of the WTMtD trajectory provided information about the preferred orientation around all rotatable bonds, including the conformation of the exocyclic hydroxymethyl group, phenyl substituent, and all hydroxyls (Figures S29–S31, Supporting Information). For example, WTMtD simulations predicted only one orientation of the phenyl substituent in all investigated compounds, where the dihedral angle C1–N–C*i*–C*o* is preferably 0° or 180°. On the other hand, the pyramidalization of the nitrogen atom oscillated between planar and pyramidal arrangements during the simulation (Figure S32, Supporting Information).

It is well accepted that the relative energies and corresponding populations of individual conformers suggested by WTMtD depend on the applied force field.^57,58^ Moreover, molecular mechanics approaches rely on predefined atom types to capture the hybridization on nitrogen atoms. This limitation can be addressed by the application of DFT methods; however, the calculation of conformer energy by DFT and its application in the conformer population determination based on the Boltzmann distribution is still challenging, especially for carbohydrates where hydroxyl groups can rotate and be involved in a cooperative hydrogen bonding network.^59^ It has been shown, for example, that DFT-calculated energy can vary as much as 6 kcal/mol depending on the rotation of the C-2 hydroxyl group in methyl d-glucopyranoside and d-mannopyranoside.^15^ A realistic picture of the conformational behavior can not be obtained by energy calculation of a single static conformer, and, at the same time, generating representative Boltzmann-weighted conformer ensembles is inherently difficult. Therefore, in the conformational analysis of *N*-phenyl d-glycopyranosylamines presented here, we focused on using ^15^N-mediated *J*-couplings as a conformational criterion.

#### DFT Calculation of *J*-Couplings

The two-step method was tested for the prediction of ^1^ *J*(H,N) in NH_3_, with an experimental value^60^ of −61.6 Hz. Using the standard contracted 6-311++G** basis set, the calculated value was −54.6 Hz. However, when the two-step method was applied with an uncontracted basis set and the addition of a tight polarization function for the core, the value improved significantly to −59.7 Hz.

In principle, *J*-coupling is sensitive to the spatial arrangement of interacting nuclei. To employ the experimental ^15^N-mediated *J*-couplings listed in Table 2 in the conformational analysis of the aniline aglycone, we first verified the sensitivity of the *J*-couplings to the conformational change. As mentioned above, the nitrogen atom in *N*-phenyl glycosylamines is pyramidal, and therefore chiral. Accordingly, we calculated the ^15^N-mediated *J*-couplings and ^3^ *J*(NH,H1) as a function of ϕ for the *R* and *S* configurations of **5b** and found that they are sensitive to both aglycone conformation and nitrogen configurations (Figure 6). The conformationally most sensitive (Tables S14 and S15, Supporting Information) was ^3^ *J*(NH,H1) with *J*-coupling value maximum differences of 13.0 Hz for (*S*)-**5b** and 14.5 Hz for (*R*)-**5b**, followed by ^2^ *J*(^15^N,H1) with *J*-coupling value maximum differences of 8.1 Hz for (*R*)-**5b** and 8.6 Hz for (*S*)-**5b**, ^1^ *J*(^15^N,C1) with *J*-coupling value maximum differences of 7.4 Hz for (*R*)-**5b** and 7.8 Hz for (*S*)-**5b**, and ^2^ *J*(^15^N,C2) with *J*-coupling value maximum differences of 5.8 Hz for (*R*)-**5b** and 6.5 Hz for (*S*)-**5b**. However, other *J*-couplings such as ^3^ *J*(^15^N,H2), ^3^ *J*(^15^N,C3), and ^3^ *J*(^15^N,C5) were found to be less susceptible to conformational changes of the aniline aglycone but may be still useful as additional parameters in the conformational analysis.

**Figure 6:**
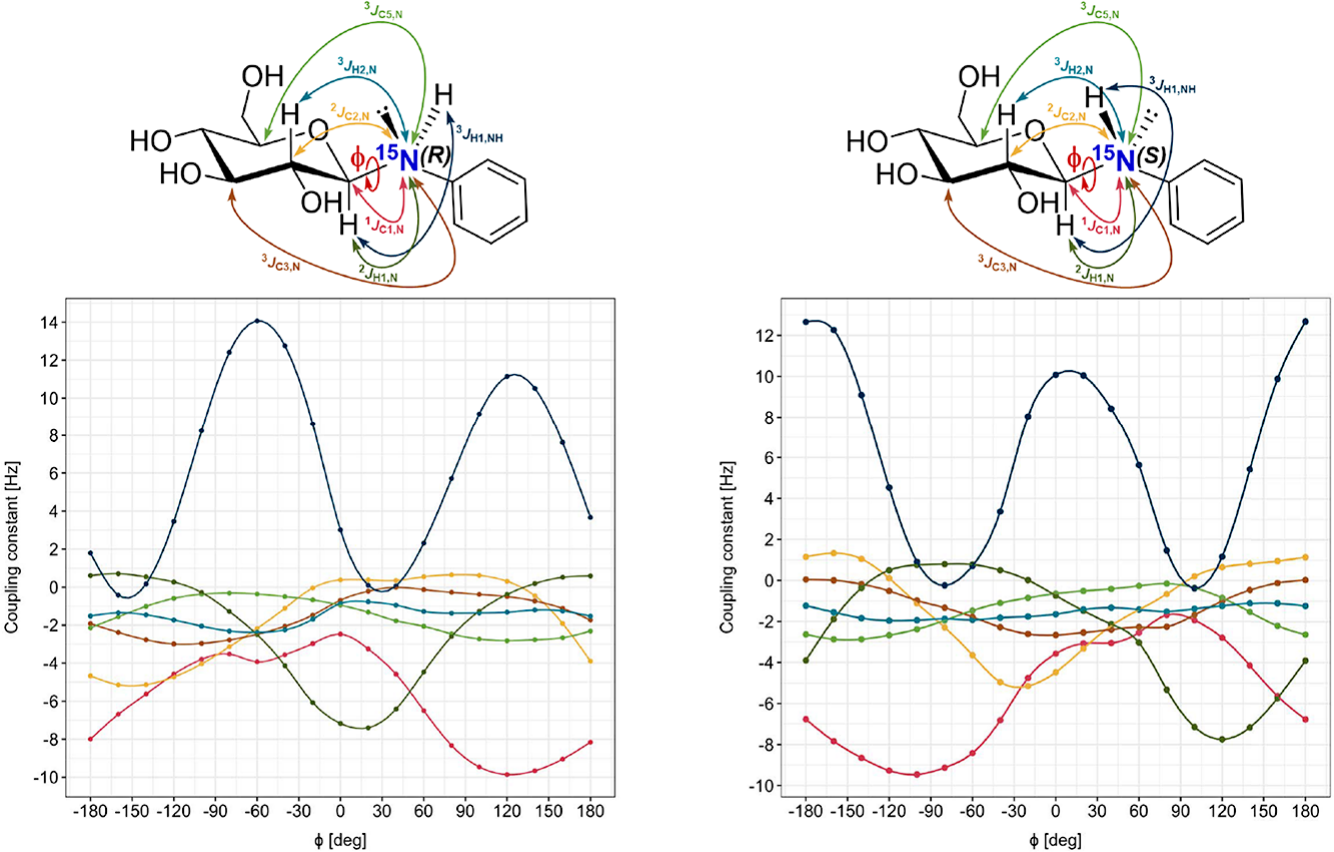
Calculated *J*-couplings as a function of ϕ for (*R*)-**5b** (left) and (*S*)-**5b** (right).

Ideally, couplings used for conformational analysis should be sensitive to anticipated conformational changes but not responsive to other changes in molecular geometry. To test this projection, we generated 54 conformers of (*R*)-**5b** with the *gt* conformation of the exocyclic hydroxymethyl group and various orientations of the hydroxyl groups, obtained from WTMtD simulations (Table S16, Supporting Information), while keeping the –*gauche* conformation of the aniline aglycone fixed. We then calculated shielding constants and *J*-couplings. We found that ^13^C chemical shift is extremely sensitive to the orientation of hydroxyl groups, experiencing maximum differences of almost 10 ppm for C3 (Table S17, Supporting Information), while *J*-couplings used for conformational analysis of the aniline aglycone remain relatively unchanged with maximum differences of 1 Hz for ^1^ *J*(^15^N,C1) (Tables S18 and S19, Supporting Information). These results encouraged us to use ^15^N-mediated *J*-couplings and ^3^ *J*(NH,H1) for conformational analysis of the aniline aglycone in **5a**, **5b**, **6a**, **6b**, and **7b**.

#### ^15^N-Mediated *J*-Coupling-Based Conformational Analysis

In the next step, we optimized the geometries of all conformers corresponding to local minima identified in Figure 5 considering both *R* and *S* configurations of the nitrogen atom for each investigated *N*-phenyl glycosylamine at the B3LYP/6-31G* DFT level using the PCM solvation model in Gaussian 16.^61^ Thus, overall 12 geometries - *(R)*-(−*gauche*), *(S)*-(−*gauche*), *(R)*-(+*gauche*), *(S)*-(+*gauche*), *(R)*-(*trans*), and *(S)*-(*trans*) with the clockwise and counterclockwise orientation of hydroxyls - were optimized for each compound. Some conformers changed their geometry in the course of the geometry optimization, and ended up with either a different aglycone conformation or an opposite nitrogen atom configuration. For example, the geometry optimization of 12 conformers of **5b** resulted in obtaining four optimized geometries *(R)*-(−*gauche*), *(S)*-(−*gauche*), *(R)*-(+*gauche*) and *(S)*-(+*gauche*). The geometries of all optimized conformers and their relative energies are shown in Figure S33, Supporting Information. Conformers with relative energy ≤ 5 kcal/mol were selected for the calculation of *J*-couplings listed in the Figure 6. The conformer populations were then obtained by a linear combination of individual calculated *J*-couplings to achieve the lowest mean absolute error (MAE) between the experimental *J*-coupling value and the absolute value of the linear combination of *J*-couplings (Tables S41–S45, Supporting Information). The MAE was 0.2 to 0.6 Hz.

The results of the conformational analysis are summarized in Table 3. We found that *N*-phenyl α-d-glycosylamines **5a** and **6a** exist in methanolic solution exclusively in the +*gauche* conformation predominantly in the *R* configuration at the nitrogen atom. This finding is in sync with the structure of *N*-α-d-glucopyranosyl anthranilic acid, in which (*R*)-(+*gauche*) conformer is found in the available crystal structure.^19^ In contrast, *N*-phenyl β-d-glycosylamines with the equatorial hydroxyl at C-2 position (**5b** and **7b**) were found to be more conformationally flexible, adopting both –*gauche* and +*gauche* conformation with a slight excess of the former. The most populated (*R*)-(−*gauche*) conformer was also coincidentally found in the crystal structure of peracetylated *N*-(4-methoxyphenyl) β-d-glucopyranosylamine.^20^ *N*-Phenyl β-d-mannopyranosylamine (**6b**) with an axial hydroxyl at the C-2 position showed a significantly different conformational behavior. This compound was found to exist primarily (81%) in the (*R*)-(−*gauche*) conformation, with the remaining 19% adopting the (*R*)-(*trans*) conformer.

**Table 3:** Percentages of Conformer Populations for Studied Compounds Obtained by the Method Employing ^15^N-Mediated Redundant *J*-Couplings

*N*-phenyl glycosylamine	(*R*)-(−*gauche*)	(*S*)-(−*gauche*)	(*R*)-(+*gauche*)	(*S*)-(+*gauche*)	(*R*)-(*trans*)	(*S*)-(*trans*)
**5a**	0	0	79	21	0	0
**5b**	33	22	17	28	0	0
**6a**	0	0	78	22	0	0
**6b**	81	0	0	0	19	0
**7b**	37	20	27	17	0	0

The results presented in Table 3 represent the conformer populations in the CD_3_OD solution, where all spectroscopic observables are averaged due to the fast exchange between conformers. Consequently, assessing the accuracy of the proposed methodology is inherently challenging. One possible validation approach is to compare the predicted conformer with the available crystal structure in cases where one conformer strongly predominates in solution. This is, for example, the case of **6b**, in which one conformation (*R*)-(−*gauche*) is more than 80% populated. We succeeded in obtaining the crystal structure of unlabeled **6b** prepared on a preparative scale (Figure 7a), which matched with the structure reported by Ojala.^21^ To our satisfaction, the conformer found in the solid state corresponded to the major conformer predicted by the method using ^15^N-mediated *J*-couplings (Figure 7c). Similarly, crystal structures of other reported substituted *N*-phenyl β-d-mannopyranosylamines^21–23^ also represent the same (*R*)-(−*gauche*) conformer. Another compound that readily crystallized was *N*-phenyl β-d-lyxopyranosylamine **8b**, whose crystal structure is shown in Figure 7b. When comparing crystal structures of **6b** and **8b**, we noticed slightly different pyramidalization of the nitrogen atom, which is shallower in the case of **8b** (see the pyramidalization angle between the C*i*–N bond and the bisector of the H–N–C1 angle in Figure 7). This variable degree of the nitrogen pyramidalization is attributed to supramolecular interactions associated with crystal packing in the solid state.^27^ The aglycone conformation in the crystal structure of **8b** and **6b** was, however, identical, further supporting the stability and preference for the (*R*)-(−*gauche*) conformer under crystallization conditions.

**Figure 7:**
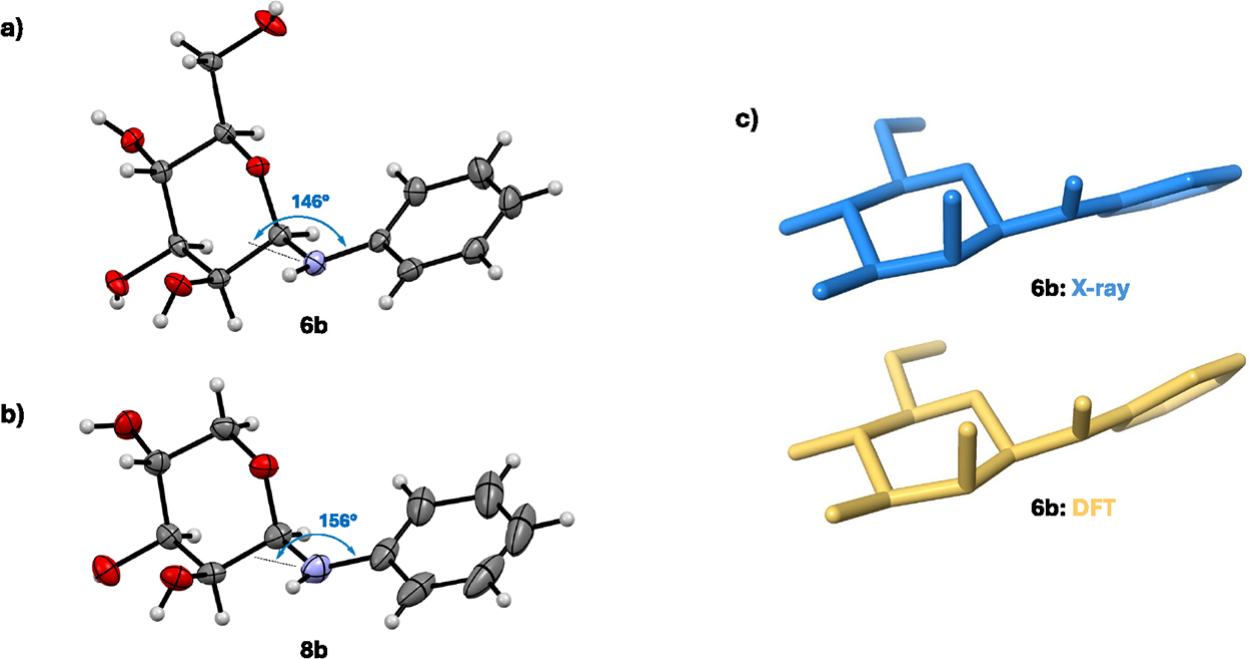
(a) Crystal structure of **6b** showing the *(R)*-(−*gauche*) conformer (thermal ellipsoids at 50% probability level). (b) Crystal structure of **8b** showing the *(R)*-(−*gauche*) conformer (thermal ellipsoids at 50% probability level). (c) Comparison of the crystal structure (blue) and the major conformer predicted by the method presented here (yellow) for **6b**.

## Conclusions

In this paper, we have proposed a method for the prediction of the aniline aglycone conformation in *N*-phenyl glycosylamines that employs ^15^N-mediated redundant *J*-couplings in solution. This method is based on the fitting of the calculated *J*-couplings of individual conformers to the experimental values. We found that ^15^N-mediated redundant *J*-couplings are sensitive to aglycone conformation and less sensitive to other conformational changes, such as the orientation of the hydroxyl groups. This enabled us to use static conformer geometries obtained by simple geometry optimization for *J*-coupling calculations. We applied this method to the conformational analysis of *N*-phenyl glycosylamines that predominantly adopt a single stable ring conformation in solution. The analysis revealed that *N*-phenyl α-d-glycopyranosylamines **5a** and **6a** exist in a methanolic solution exclusively in the +*gauche* conformation, whereas *N*-phenyl β-d-glycopyranosylamines **5b** and **7b** are more conformationally flexible, populating both –*gauche* and +*gauche* conformations, with a slight preference for the –*gauche* conformer. Moreover, this method also predicts the preferred configuration of the pyramidal nitrogen atom. It enabled identification of the major (*R*)-(−*gauche*) conformer of *N*-phenyl β-d-mannopyranosylamine (**6b**), which was confirmed by X-ray cystallography.

## Experimental Section

### General Methods and Materials

^1^H and ^13^C NMR spectra were acquired using Bruker AVANCE III HD 500 MHz (^1^H at 500.0 MHz, ^13^C at 125.7 MHz), Bruker AVANCE III HD 600 MHz (^1^H at 600.1 MHz, ^13^C at 150.9 MHz) and Jeol JNM-ECZR 500 MHz (^1^H at 500.2 MHz, ^13^C at 125.8 MHz) spectrometers. The variable temperature NMR experiments were carried out on Bruker AVANCE II 500 MHz (^1^H at 500.0 MHz, ^13^C at 125.7 MHz). NMR spectra were referenced to the signal of the solvent. ^15^N-nitromethane (381.7 ppm in ^15^N) was used as an external reference standard for ^1^H, ^15^N-HMBC technique. Complete assignment of ^1^H and ^13^C resonances was performed using H,H-COSY and H,C-HSQC techniques. For more accurate determination of *J*-couplings in ^1^H NMR, spectra were processed by Mnova, FIDs were zero-filled twice, and apodized by exponential function (LB = −2 Hz) and Gaussian function (GB = 1 Hz). No apodization was applied when reading *J*-couplings in ^13^C NMR spectra.

Infrared (IR) spectra were recorded using a Nicolet 6700 spectrometer (Thermo Fisher Scientific, USA). Absorption maxima (νmax) are reported in wavenumbers (cm^–1^).

High-resolution mass spectra were measured on an LTQ Orbitrap XL spectrometer (Thermo Fisher Scientific, USA) using ESI ionization. Nominal and exact *m*/*z* values are reported in Daltons.

Melting points (m.p.) were recorded on an MPM-HV3 (Schorpp-Gerätetechnik, Germany) capillary melting-point meter and are reported uncorrected in degrees Celsius (°C).

Crystals of **6b** and **8b** suitable for X-ray analysis were obtained by slow crystallization from ethanol. Single-crystal diffraction data were collected using Bruker D8 VENTURE system equipped with a Photon 100 CMOS detector, a multilayer monochromator, and a CuKα Incoatec microfocus sealed tube (λ = 1.54178 Å) at 180 K. The frames were integrated with the Bruker SAINT^62^ software package. Structures were solved by direct methods with SIR92 ^63^ and refined by full-matrix least-squares on F with CRYSTALS.^64^ The positional and anisotropic thermal parameters of all non-hydrogen atoms were refined. All hydrogen atoms were located in a difference Fourier map, but those attached to carbon atoms were repositioned geometrically. They were initially refined with soft restraints on the bond lengths and angles to regularize their geometry, then their positions were refined with riding constraints.

Anhydrous solvents were used directly as received from commercial suppliers (Sigma-Aldrich or Acros Organics). Anhydrous methanol-*d*_4_ was obtained by refluxing commercial methanol-*d*_4_ with magnesium turnings and iodine, and by distilling off the dry solvent. All the other solvents were utilized as supplied (analytical or HPLC grade) without prior purification. Reagents were purchased from various commercial suppliers and used as supplied, unless otherwise indicated. Distilled water was employed for chemical reactions.

### General Procedures for the Preparation of *N*-Phenyl Glycosylamines

#### In Situ Preparation in an NMR Tube

A solution of the corresponding monosaccharide (5 mg; 28 μmol for hexoses, 33 μmol for pentoses, 1.0 equiv) in 0.6 mL of anhydrous methanol-*d*_4_ was heated in an NMR tube at 60 °C in an aluminum heating block for 1 h to ensure complete mutarotation. After cooling to room temperature, ^15^N-aniline (3.4 mg, 36 μmol for hexoses; 4.0 mg, 43 μmol for pentoses, 1.3 equiv) and a drop of acetic acid-*d*_4_ (2 μL, 0.12 equiv) were added. The NMR tube was sealed and heated at 60 °C for 8–38 h (Table 1) to allow equilibrium to be reached. The conversion of all monosaccharides exceeded 90% (Table 1).

#### Preparative-Scale Synthesis of *N*-Phenyl Glycosylamines

Generally, to a suspension of the respective monosaccharide (2 mmol, 1.0 equiv) in anhydrous methanol 10 mL, aniline (200 μL, 2.2 mmol, 1.2 equiv) and acetic acid (10 μL, 0.18 mmol, 0.1 equiv) were added. The reaction mixture was heated in an aluminum heating block with stirring at 60 °C for 1 or 20 h. Reaction progress was monitored by thin-layer chromatography (ACN/H_2_O, 19:1). Upon complete conversion of the starting material, the reaction mixture was cooled to room temperature and filtered. The resulting crystalline product was washed with cold EtOH and Et_2_O and dried at room temperature under vacuum. The filtrate was concentrated and the residue was crystallized. The product was collected by filtration, washed as above, and dried under vacuum.

#### Unlabeled *N*-Phenyl β-d-Glucopyranosylamine (**5b**)

Prepared from d-glucose (360 mg, 2 mmol) following the general procedure (reaction time: 20 h). The product **5b** was isolated as a white crystalline powder (254 mg, 55%) after filtration and recrystallization from hot ethanol. **M.p.**: 135–136 °C (decomposition); (lit. 135–136 °C).^65^ **IR** (CH_3_OH): 3357, 3057, 3031, 2927, 2878, 1604, 1592, 1500, 1443, 1268, 1178, 1156, 1099, 1075, 1035, 995, 752 cm^–1^. **HRMS** (ESI) [M + Na]^+^ *m*/*z* calculated for C_12_H_17_O_5_NNa 278.0997, found 278.0999; [M + H]^+^ *m*/*z* calculated for C_12_H_18_O_5_N 256.1180, found 256.1179. ^**1**^ **H NMR** and ^**13**^ **C­{** ^**1**^ **H} NMR** assignment has been already published.^31^

#### Unlabeled *N*-Phenyl β-d-Mannopyranosylamine (**6b**)

Prepared from d-mannose (360 mg, 2 mmol) following the general procedure (reaction time: 20 h). The product **6b** was isolated as colorless needles (313 mg, 61%) after filtration and recrystallization from hot ethanol. **M.p.**: 180–181 °C (decomposition); (lit. 181 °C).^66^ **IR** (CH_3_OH): 3529, 3412, 3337, 3088, 3059, 3023, 1604, 1584, 1512, 1440, 1179, 1073, 1060, 1042, 1037, 1026, 995, 872, 820, 745, 691, 500, 427 cm^–1^. **HRMS** (ESI) [M + Na]^+^ *m*/*z* calculated for C_12_H_17_O_5_NNa 278.0999, found 278.0999; [M + H]^+^ *m*/*z* calculated for C_12_H_18_O_5_N 256.1180, found 256.1179. ^**1**^ **H NMR** (600.1 MHz, methanol-*d*_4_): δ 7.16–7.10 (m, 2H, H*m*), 6.79–6.75 (m, 2H, H*o*), 6.70 (tt, *J*_*p*,*m*_ = 7.4 Hz, *J*_*p*,*o*_ = 1.1 Hz, 1H, H*p*), 4.88 (in the water signal, H-1), 3.91 (dd, *J*_2,3_ = 3.2 Hz, *J*_2,1_ = 1.1 Hz, 1H, H-2), 3.84 (dd, *J*_6*a*,6*b*_ = 11.9 Hz, *J*_6*a*,5_ = 2.5 Hz, 1H, H-6a), 3.70 (dd, *J*_6*b*,6*a*_ = 11.9 Hz, *J*_6*b*,5_ = 5.6 Hz, 1H, H-6b), 3.61 (t, *J*_4,3_ = *J*_4,5_ = 9.3 Hz, 1H, H-4), 3.57 (dd, *J*_3,4_ = 9.5 Hz, *J*_3,2_ = 3.3 Hz, 1H, H-3), 3.32 (ddd, *J*_5,4_ = 9.3 Hz, *J*_5,6*b*_ = 5.5 Hz, *J*_5,6*a*_ = 2.5 Hz, 1H, H-5). ^**13**^ **C­{** ^**1**^ **H} NMR** (150.9 MHz, methanol-*d*_4_): δ 147.1 (C*i*), 130.0 (CH-*m*), 119.6 (CH-*p*), 115.2 (CH-*o*), 83.6 (CH-1), 78.8 (CH-5), 76.2 (CH-3), 73.1 (CH-2), 68.7 (CH-4), 62.9 (CH_2_-6). **Crystal data** (colorless, 0.033 × 0.104 × 0.116 mm): C_12_H_17_N_1_O_5_, orthorhombic, space group *P*2_1_2_1_2_1_, *a* = 6.44150(10) Å, *b* = 6.73240(10) Å, *c* = 28.1270(5) Å, *V* = 1219.78(2) Å^3^, *Z* = 4, M = 255.27, 14156 reflections measured, 2234 independent reflections. Final R = 0.027, *wR* = 0.029, *GoF* = 1.071 for 2168 reflections with *I* > 2σ­(*I*) and 164 parameters. Flack parameter *x* = 0.22(13). CCDC 2404410.

#### Unlabeled *N*-Phenyl β-D-Xylopyranosylamine (**7b**)

Prepared from d-xylose (300 mg, 2 mmol) following the general procedure (reaction time: 1 h). The product **7b** was isolated as colorless plates (257 mg, 57%) after filtration and recrystallization from ethanol. **M.p.**: 142–144 °C (decomposition); (lit. 144–145 °C).^66^ **IR** (CH_3_OH): 3346, 3056, 2968, 2908, 2862, 1604, 1514, 1499, 1443, 1178, 1156, 1041, 1041, 824, 752, 694, 510 cm^–1^. **HRMS** (ESI) [M + Na]^+^ *m*/*z* calculated for C_11_H_15_O_4_NNa 248.0893, found 248.0891; [M + H]^+^ *m*/*z* calculated for C_11_H_16_O_4_N 226.1074, found 226.1072. ^**1**^ **H NMR** (500.0 MHz, methanol-*d*_4_): δ 7.16–7.10 (m, 2H, H*m*), 6.77–6.74 (m, 2H, H*o*), 6.71 (tt, *J*_*p*,*m*_ = 7.4 Hz, *J*_*p*,*o*_ = 1.1 Hz, 1H, H*p*), 4.49 (d, *J*_1,2_ = 8.5 Hz, 1H, H-1), 3.83 (dd, *J*_5*a*,5*b*_ = 11.3 Hz, *J*_5*a*,4_ = 5.3 Hz, 1H, H-5a), 3.52 (ddd, *J*_4,5*b*_ = 10.3 Hz, *J*_4,3_ = 8.9 Hz, *J*_4,5*a*_ = 5.3 Hz, 1H, H-4), 3.41 (t, *J*_3,4_ = *J*_3,2_ = 8.8 Hz, 1H, H-3), 3.32 (dd, *J*_5*b*,5*a*_ = 11.3 Hz, *J*_5*b*,4_ = 10.3 Hz, 1H, H-5b), 3.30 (dd, *J*_2,3_ = 8.8 Hz, *J*_2,1_ = 8.5 Hz, 1H, H-2). ^**13**^ **C­{** ^**1**^ **H} NMR** (125.7 MHz, methanol-*d*_4_): δ 147.9 (C*i*), 130.0 (CH-*m*), 119.6 (CH-*p*), 115.0 (CH-*o*), 87.6 (CH-1), 78.9 (CH-3), 74.5 (CH-2), 71.4 (CH-4), 67.5 (CH_2_-5).

#### Unlabeled *N*-Phenyl β-d-Lyxopyranosylamine (**8b**)

Prepared from d-lyxose (300 mg, 2 mmol) following the general procedure (reaction time: 1 h). The product **8b** was isolated as colorless plates (344 mg, 76%) after filtration and recrystallization from hot ethanol. **M.p.**: 144–145 °C (decomposition); (lit. 145–146 °C).^67^ **IR** (CH_3_OH): 3325, 3053, 3028, 2925, 2855, 1603, 1590, 1584, 1508, 1455, 1441, 1179, 1151, 1089, 1075, 1059, 1037, 1028, 995, 875, 821, 749, 691, 506, 420 cm^–1^. **HRMS** (ESI) [M + Na]^+^ *m*/*z* calculated for C_11_H_15_O_4_NNa 248.0893, found 248.0892; [M + H]^+^ *m*/*z* calculated for C_11_H_16_O_4_N 226.1074, found 226.1073. ^**1**^ **H NMR** (600.1 MHz, methanol-*d*_4_): δ 7.15–7.10 (m, 2H, H*m*), 6.75–6.72 (m, 2H, H*o*), 6.70 (tt, *J*_*p*,*m*_ = 7.4 Hz, *J*_*p*,*o*_ = 1.0 Hz, 1H, H*p*), 4.87 (in the water signal, H-1), 3.95 (dd, *J*_2,3_ = 3.4 Hz, *J*_2,1_ = 2.0 Hz, 1H, H-2), 3.86 (dd, *J*_5*a*,5*b*_ = 11.5 Hz, *J*_5*a*,4_ = 4.7 Hz, 1H, H-5a), 3.78 (td, *J*_4,3_ = *J*_4,5*b*_ = 8.5 Hz, *J*_4,5*a*_ = 4.7 Hz, 1H, H-4), 3.59 (dd, *J*_3,4_ = 8.3 Hz, *J*_3,2_ = 3.4 Hz, 1H, H-3), 3.25 (dd, *J*_5*b*,5*a*_ = 11.5 Hz, *J*_5*b*,4_ = 8.7 Hz, 1H, H-5b). ^**13**^ **C**{^**1**^ **H**} **NMR** (150.9 MHz, methanol-*d*_4_): δ 147.1 (C*i*), 130.0 (CH-*m*), 119.5 (CH-*p*), 115.1 (CH-*o*), 84.2 (CH-1), 75.5 (CH-3), 71.4 (CH-2), 68.9 (CH-4), 66.2 (CH_2_-5). **Crystal data** (colorless, 0.060 × 0.186 × 0.204 mm): C_11_H_15_N_1_O_4_, orthorhombic, space group *P*2_1_2_1_2_1_, *a* = 6.05670(10) Å, *b* = 6.4441(2) Å, *c* = 28.6219(7) Å, *V* = 1117.11(3) Å^3^, *Z* = 4, M = 225.24, 23689 reflections measured, 2122 independent reflections. Final R = 0.023, *wR* = 0.024, *GoF* = 1.064 for 2116 reflections with *I* > 2σ­(*I*) and 201 parameters. Flack parameter *x* = 0.12(11). The phenyl group is disordered in two positions. The site occupancy factors of the two disordered parts were refined to 0.59(3) and 0.41(3). CCDC 2404411.

### Computational Methods

#### Well-Tempered Metadynamics Simulations

Well-tempered metadynamics simulations (WTMtD) were carried out using the Desmond program^68^ (Schrödinger Release 2021-03 ^69,70^ ) for a cubic simulation box containing one molecule of *N*-phenyl glycosylamine and approximately 300 methanol molecules. The initial structure of the solute was optimized prior to simulation. Each simulation lasted 100 ns with a time step of 1 fs in the NPT ensemble at a pressure of 1.01325 bar. The temperature (300 K) and pressure were maintained using the Nose–Hoover thermostat and Martyna–Tobias–Klein barrostat.^71^ The long-range electrostatic interactions were calculated using the particle mesh Ewald method.^72^ The cutoff radius of Coulomb interactions was 9.0 Å. Periodic boundary conditions were applied, and trajectories were saved every 10 ps, resulting in 10,000 frames. The system was relaxed using a standard procedure in Desmond (containing partial optimizations, annealing, and equilibration) before the actual simulation. The OPLS4 force field was used to describe both the solute and methanol solvent. Metadynamics parameters included a hill height of 0.03 kcal/mol, a deposition interval of 0.09 ps, and a bias factor (kTemp) of 2.4, assuming an energy barrier of approximately 4 kcal/mol. The bias was applied to the dihedral angle ϕ to promote conformational sampling.

#### DFT Calculations

All DFT calculations were performed in Gaussian 16.^61^ Geometry optimizations were performed using the density functional theory (DFT) at the B3LYP/6-31G* level. The Polarizable Continuum Model (PCM)^73^ was applied to mimic the methanol solvent. Frequency calculations were carried out at the same level of theory to confirm that optimized structures correspond to true energy minima. NMR parameters, including *J*-coupling constants and ^13^C shielding constants, were calculated at the B3LYP/6-311++G** level of theory using the two-step “mixed” approach,^74^ in which the basis set is uncontracted and tight polarization functions for the core are added to treat better the Fermi contact contribution.

To assess the sensitivity of the *J*-coupling constants to the aglycone conformation, a systematic scan of a selected dihedral angle was conducted. The ϕ dihedral angle was rotated in 20° increments, followed by optimization with the fixed nitrogen configuration and calculation of coupling constants.

To evaluate the influence of hydroxyl group orientation to *J*-couplings and ^13^C shielding constants, a set of geometries was generated for (*R*)-(−*gauche*) **5b** with *gt* conformation of the hydroxymethyl group. Dihedral angles of the 2-OH, 3-OH, 5-OH, and 6-OH hydroxyl groups were manually adjusted to reflect dominant orientations observed in WTMtD simulations. These geometries were not optimized, and NMR parameters were calculated directly.

For the analysis of aglycone conformational populations, a comprehensive set of geometries was constructed encompassing all three major aglycone conformers (−*gauche*, +*gauche*, *trans*), both nitrogen configurations (*R* and *S*), and two orientations (clockwise and counterclockwise) for hydroxyl groups. In the case of compound **5a**, an additional orientation was included for the 2-OH group. All geometries in this set were optimized, and their relative energies were calculated. Conformers within 5 kcal/mol of the lowest-energy structure were selected for further NMR calculations.

The influence of hyperconjugation, lone pair delocalization, and electrostatic repulsion on the anomeric preferences was assessed by DFT calculations performed for the local minima of **5** and **6**, described in Supporting Information, Figure S33. First, Gibbs free energies were calculated for all conformers at the B3LYP/aug-cc-pVTZ level with the GD3BJ empirical dispersion correction and the PCM solvent model to mimic the methanol solution. Boltzmann populations were estimated based on the obtained free energies. To calculate the total populations of individual anomers (Table S13, av. p), the conformers of both anomers were grouped together, their populations were calculated, and then the populations of conformers corresponding to one anomer were added together. For the calculation of other average values in Table S13, populations of conformers within a single anomer were calculated and used for Boltzmann averaging. The hyperconjugation effect was estimated at the B3LYP level with the GD3BJ empirical dispersion and the PCM model using NBO 3.1 ^75^ distributed as a part of Gaussian 16. The energy of the perfectly localized system with all doubly occupied Lewis natural bond orbitals (NBO) was subtracted from the conformer energy obtained using the 6-311++G** basis set, similarly to ref 76. The delocalization of the lone pairs at O-5 and N-1 was estimated at the B3LYP/6-311++G**/PCM level with the GD3BJ empirical dispersion for all conformers. The same level of theory was used to calculate dipole moments. Hyperconjugation, lone pair delocalization, and dipole moments were Boltzmann averaged for each anomer, and average values were obtained for compounds **5a**, **5b**, **6a**, and **6b**.

## Supplementary Material



## Data Availability

The data underlying this study are available in the published article and its Supporting Information. Primary data, including raw NMR data (FIDs), input files for well-tempered metadynamics simulations, and input and log files for DFT calculations, are available via the Open Science Framework repository:10.17605/OSF.IO/JBDWU.

## Notes

Additional experimental and theoretical results, including kinetics monitoring, complete NMR assignment, copies of NMR spectra; analysis of ring conformation; calculations of energy contributions related to the reverse anomeric effect; torsion profiles obtained by well-tempered metadynamics simulations; DFT geometry optimizations accompanied by Cartesian coordinates; *J*-coupling calculations; fitting of calculated *J*-couplings to experimental ones resulting in conformer populations; and crystallographic data (PDF)
