# Supplementary material for: Redundant 15N‑Mediated J‑Couplings Reveal an Aglycone Conformation in N‑Phenyl Glycosylamines
Source: J Org Chem. 2025 Oct 31;90(45):16047–59. doi: 10.1021/acs.joc.5c01892 (PMC12624841; doi:10.1021/acs.joc.5c01892)
Supplement: Supplementary file 1 [file jo5c01892_si_001.pdf]

Supporting Information

Redundant ^{15}N-mediated J-couplings reveal an aglycone conformation in N-phenyl glycosylamines

Nina Habanová[†, ‡], Jakub Kaminský[†], Kamil Parkan[§, †], Jakub Zýka[§, †], Vít Prouza[§, †], Blanka Klepetářová[†], and Radek Pohl[†,*]

[†]Institute of Organic Chemistry and Biochemistry, Czech Academy of Sciences, Flemingovo náměstí 542/2, Prague 6, 166 10, Czech Republic
[‡]Department of Analytical Chemistry, University of Chemistry and Technology, Prague, Technická 5, 166 28 Prague, Czech Republic
[§]Department of Chemistry of Natural Compounds, University of Chemistry and Technology, Prague, Technická 5, 166 28 Prague, Czech Republic

Contents

1 *N*-phenyl^{15}N-glycosylamines *in situ* preparation **S2**
1.1 Reaction kinetics . . . S2
1.2 Mutarotation of starting monosaccharides in CD_3OD . . . S4

2 NMR data **S5**
2.1 NMR assignment of N-phenyl^{15}N-glycosylamines . . . S5
2.2 NMR spectra of N-phenyl^{15}N-glycosylamines . . . S6
2.3 NMR spectra of unlabeled N-phenyl-glycosylamines . . . S19

3 Determination of the pyranose ring conformation **S22**

4 Determination of the anomeric configuration **S24**

5 Determination of the aglycone conformation **S25**
5.1 Well-tempered metadynamics simmulations . . . S25
5.2 DFT calculations . . . S28

6 Crystallographic data **S56**

1 *N*-phenyl^{15}N-glycosylamines *in situ* preparation

1.1 Reaction kinetics

The formation of the products was observed by ^{1}H-NMR (Figures S1 to S4). The reaction rate constants were calculated by integration of *ortho*-hydrogen ^{1}H signals of both *N*-phenyl^{15}N-glycosylamine anomers over time.

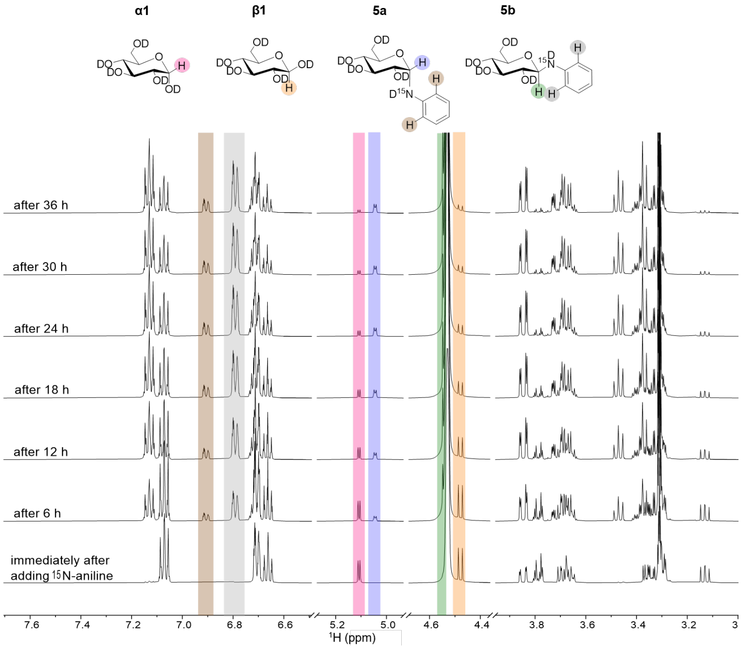

Figure S1: ^{1}H NMR spectra (500 MHz, 333 K) showing the reaction of **1** with ^{15}N-aniline in methanol-d_4, catalyzed by acetic acid-d_4, and formation of products **5a** and **5b**.

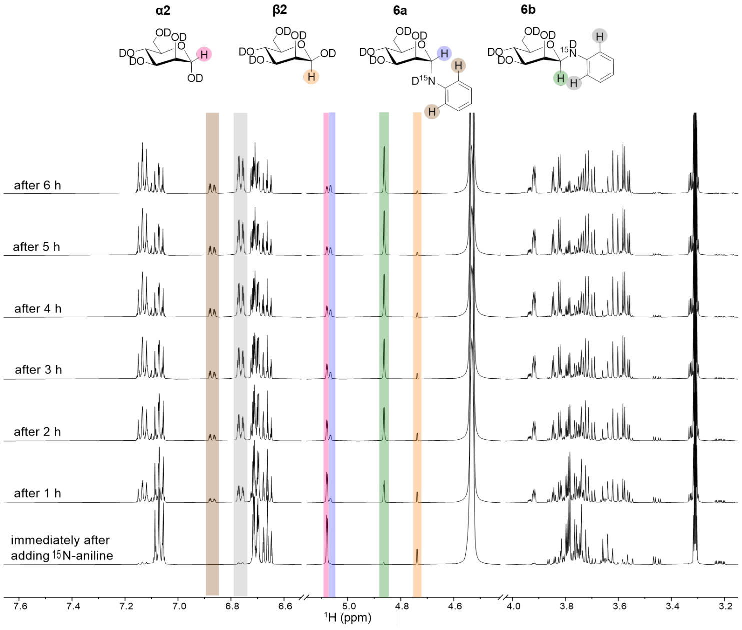

Figure S2: ^{1}H NMR spectra (500 MHz, 333 K) showing the reaction of **2** with ^{15}N-aniline in methanol-d_4, catalyzed by acetic acid-d_4, and formation of products **6a** and **6b**.

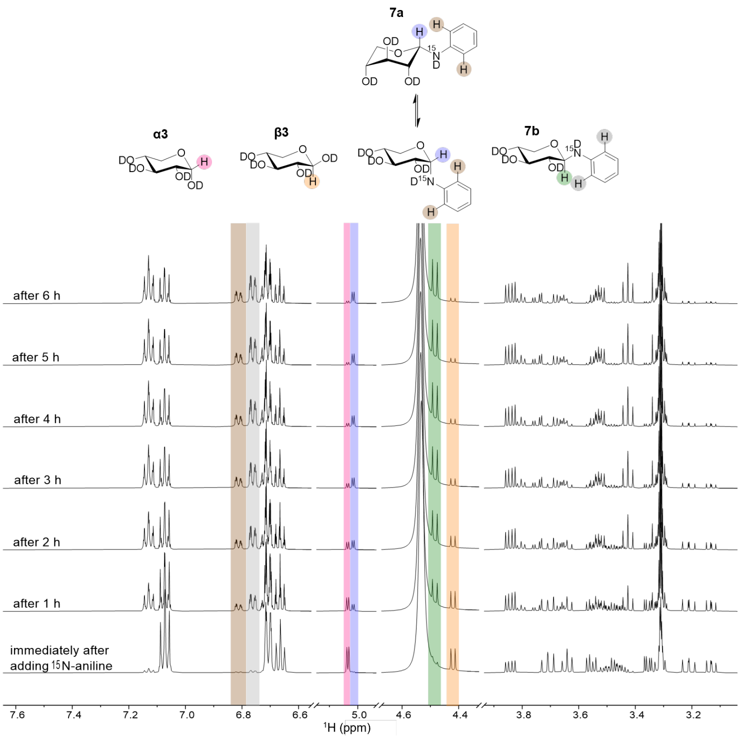

Figure S3: ^{1}H NMR spectra (500 MHz, 333 K) showing the reaction of **3** with ^{15}N-aniline in methanol-d_4, catalyzed by acetic acid-d_4, and formation of products **7a** and **7b**.

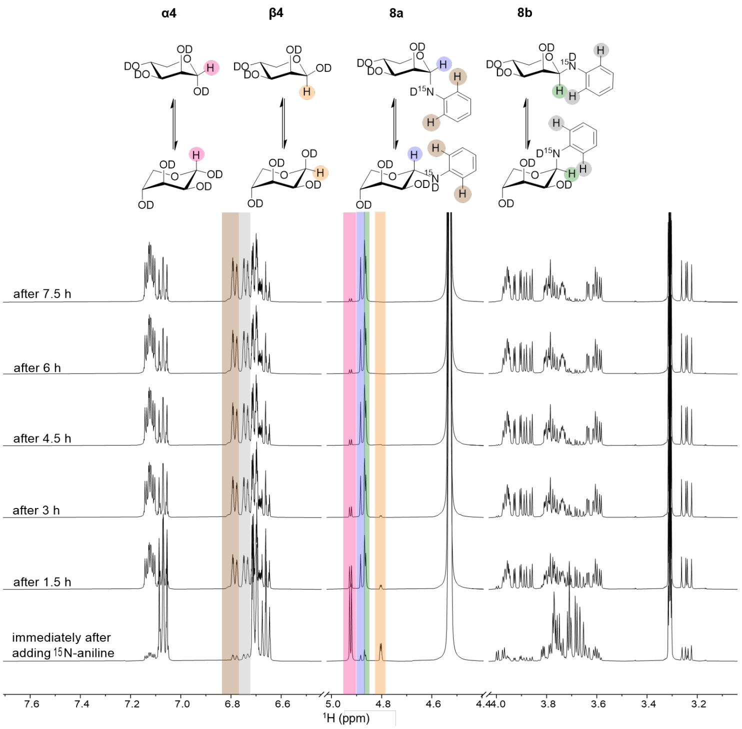

Figure S4: ^{1}H NMR spectra (500 MHz, 333 K) showing the reaction of **4** with ^{15}N-aniline in methanol-d_4, catalyzed by acetic acid-d_4, and formation of products **8a** and **8b**.

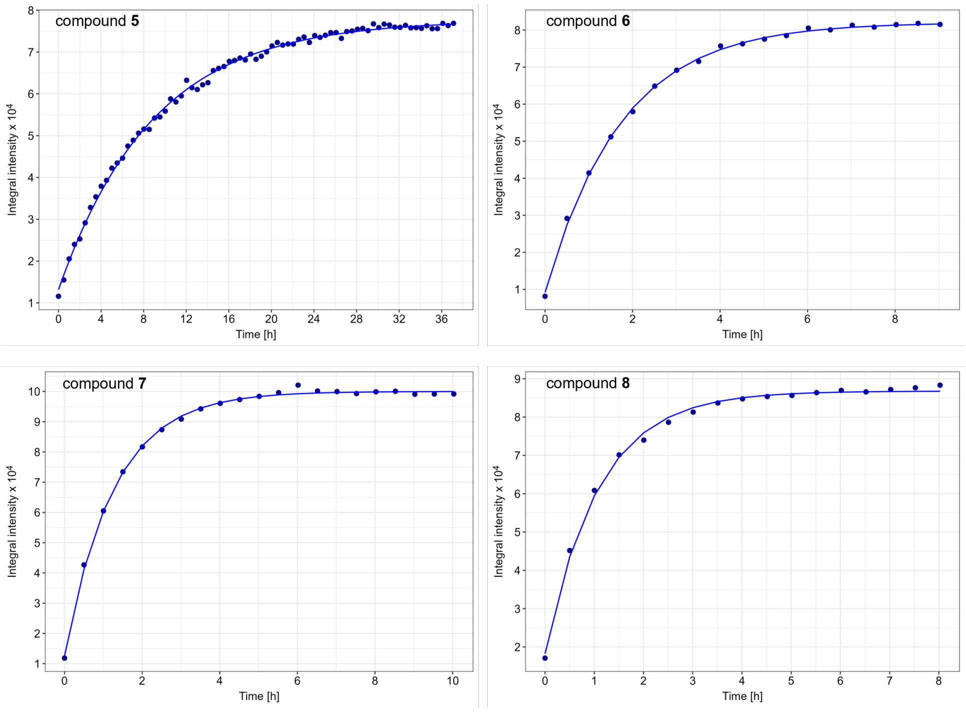

Figure S5: Time dependence of the integral intensity of *ortho*-hydrogen ^{1}H signals of both N-phenyl^{15}N-glycosylamine anomers.

Table S1: Rate constants of N-phenyl^{15}N-glycosylamine formations.

	Rate constant [s^{-1}]	
Starting sugar	Catalyzed reaction	Uncatalyzed reaction
**1**	3.1×10^{-5}	n.d.
**2**	1.6×10^{-4}	n.d.
**3**	2.2×10^{-4}	2.1×10^{-5}
**4**	2.5×10^{-4}	8.8×10^{-5}

1.2 Mutarotation of starting monosaccharides in CD_3OD

Table S2: Anomeric ratio of monosaccharides in CD_3OD.

Monosaccharide anomeric ratio [%]	Immediately after dissolution		Equilibrium in CD_3OD		Equilibrium in CD_3OD + AcOD	
	α	β	α	β	α	β
**1**	98	2	47	53	49	51
**2**	95	5	83	17	82	18
**3**	94	6	50	50	60	40
**4**	4	96	81	19	78	22

2 NMR data

2.1 NMR assignment of *N*-phenyl^{15}N-glycosylamines

Table S3: Experimental NMR ^{1}H, ^{13}C and ^{15}N chemical shifts of *N*-phenyl^{15}N-glycosylamines in methanol-d_4.

δ [ppm]	**5a**	**5b**	**6a**	**6b**	**7a**	**7b**	**8a**	**8b**
H1	5.03	4.54	5.03	4.88	5.01	4.49	4.89	4.88
H2	3.68 – 3.71	3.31	3.93	3.91	3.64	3.30	3.80	3.95
H3	3.68 – 3.71	3.48	3.83	3.57	3.78	3.41	3.96	3.59
H4	3.62 – 3.67	3.35	3.71	3.61	3.51	3.52	3.74	3.78
H5	3.62 – 3.67	3.38	3.57	3.31	3.71	3.83	3.92	3.87
H5'	-	-	-	-	3.65	3.32	3.62	3.25
H6	3.73	3.85	3.75	3.84	-	-	-	-
H6'	3.71	3.67	3.72	3.70	-	-	-	-
H*o*	6.89 – 6.93	6.80 – 6.76	6.85 – 6.88	6.75 – 6.78	6.80 – 6.83	6.73 – 6.77	6.76 – 6.79	6.73 – 6.76
H*m*	7.11 – 7.15	7.11 – 7.15	7.11 – 7.16	7.11 – 7.16	7.10 – 7.15	7.10 – 7.15	7.10 – 7.15	7.10 – 7.15
H*p*	6.72	6.72	6.70	6.70	6.71	6.71	6.71	6.69
C1	84.77	86.96	85.93	83.58	83.19	87.66	84.25	84.16
C2	72.57	74.70	72.66	73.11	72.56	74.54	69.99	71.37
C3	75.41	79.14	72.91	76.22	73.34	78.94	72.53	75.49
C4	71.93	71.80	69.16	68.66	71.01	71.42	70.97	68.85
C5	72.04	78.45	73.15	78.77	64.03	67.51	65.73	66.22
C6	62.62	62.77	62.81	62.86	-	-	-	-
C*i*	148.71	148.11	147.91	147.11	147.93	147.93	148.10	147.10
C*o*	115.13	115.27	115.15	114.88	115.13	115.04	115.07	114.99
C*m*	130.00	129.90	129.89	130.05	129.97	129.99	129.94	130.02
C*p*	119.59	119.55	119.55	119.24	119.59	119.60	119.51	119.38
^{15}N	68.6	78.3	70.0	75.9	70.8	77.6	76.2	74.2

Table S4: Experimental ^{1}H-^{1}H, ^{15}N-^{1}H, and ^{15}N-^{13}C *J*-couplings of *N*-phenyl^{15}N-glycosylamines in methanol-d_4

J-coupling [Hz]	**5a**	**5b**	**6a**	**6b**	**7a**	**7b**	**8a**	**8b**
^{3}J(H1,H2)	4.2	8.7	1.8	1.0	3.6	8.5	7.6	2.0
^{3}J(H2,H3)	nd	8.7	3.4	3.2	6.8	8.7	3.2	3.4
^{3}J(H3,H4)	nd	8.7	9.3	9.3	6.8	8.9	4.8	8.3
^{3}J(H4,H5)	nd	9.6	nd	9.3	4.0	5.3	2.2	4.7
^{3}J(H4,H5')	-	-	-	-	7.1	10.4	3.3	8.7
^{3}J(H5,H6)	3.2	2.3	4.7	2.5	-	-	-	-
^{3}J(H5,H6')	4.2	5.2	3.0	5.5	-	-	-	-
^{2}J(H5,H5')	-	-	-	-	11.7	11.3	12.1	11.5
^{2}J(H6,H6')	11.9	12.0	11.7	11.9	-	-	-	-
^{4}J(H3,H5)	0	0	0	0	0	0	1.0	0
^{3}J(NH,H1)	3.4	8.5	4.6	9.8	5.0	8.6	7.7	nd
^{3}J(^{15}N,H2)	nd	2.1	0.5	1.0	1.8	2.0	1.7	1.3
^{1}J(^{15}N,C1)	10.9	13.1	11.2	12.7	11.8	13.1	12.5	12.1
^{2}J(^{15}N,C2)	1.3	1.3	3.5	0.9	1.2	1.2	1.7	b
^{3}J(^{15}N,C3)	0	2.3	0	2.2	b	2.3	1.3	b
^{3}J(^{15}N,C5)	1.1	1.4	0.8	1.2	b	1.6	1.0	b
^{1}J(^{15}N,C*i*)	13.0	13.7	13.6	13.9	13.7	13.7	13.7	14.0
^{2}J(^{15}N,C*o*)	2.3	2.3	2.3	2.1	2.3	2.3	2.3	2.3
^{3}J(^{15}N,C*m*)	1.4	1.4	1.4	1.3	1.3	1.3	1.4	1.4

nd: not determined due to the signal overlap

2.2 NMR spectra of *N*-phenyl^{15}N-glycosylamines

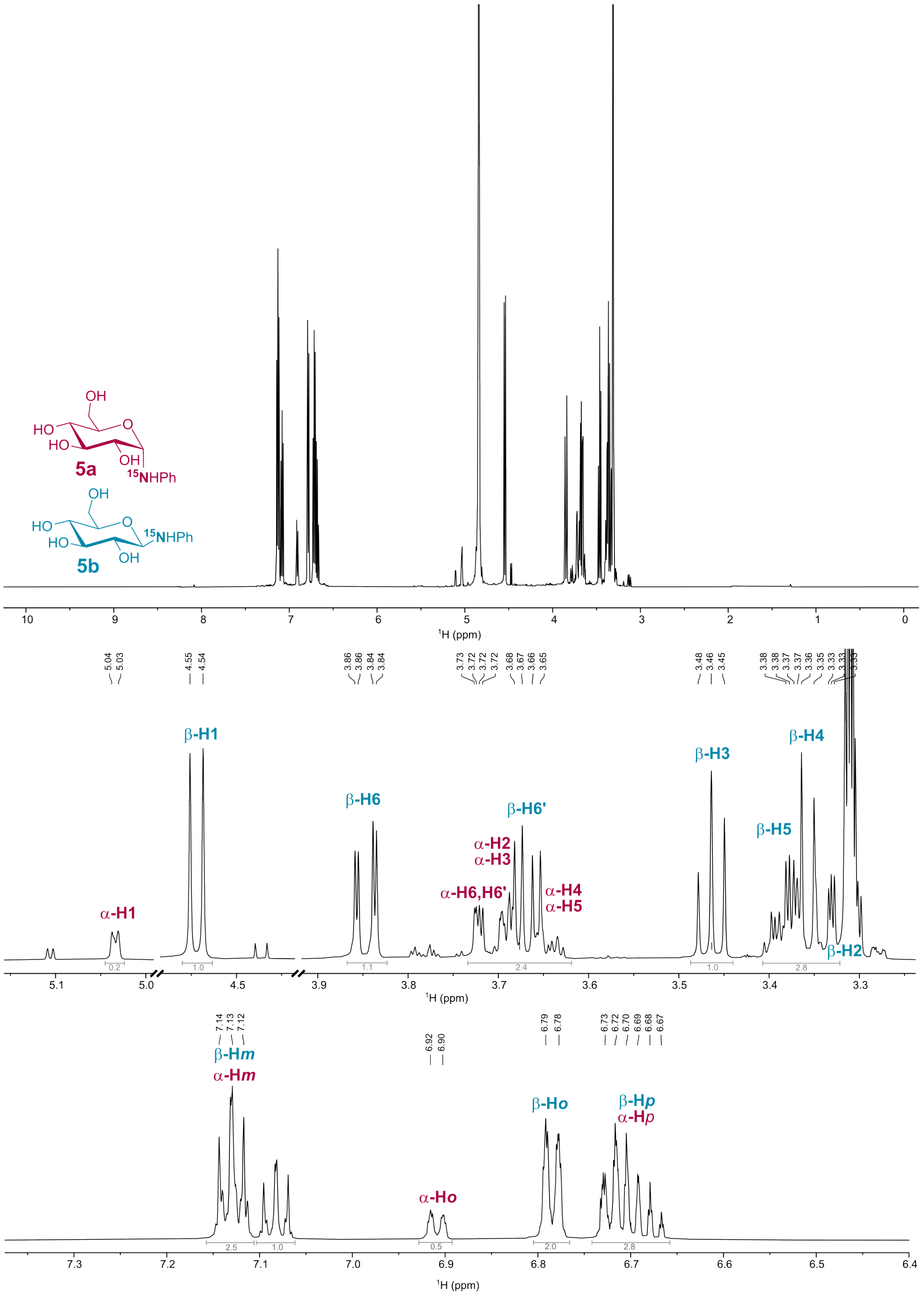

Figure S6: ^{1}H NMR spectrum (600 MHz, 298 K) of **5a** and **5b** as products of the *in situ* reaction of **1** with ^{15}N-aniline in methanol-d_4. The spectrum also contains signals of unreacted starting substances.

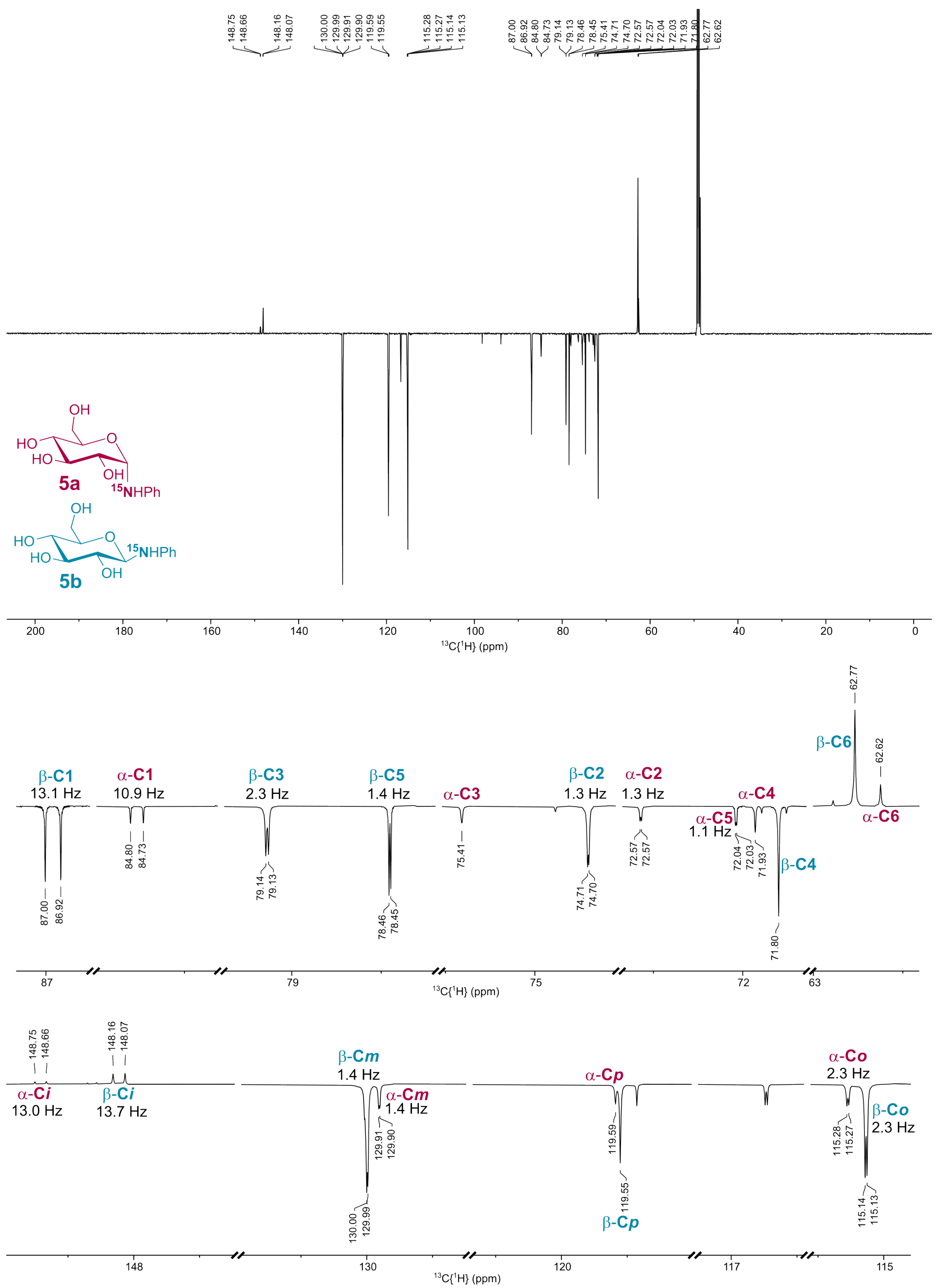

Figure S7: ^{13}C-APT NMR spectrum (151 MHz, 298 K) of **5a** and **5b** as products of the *in situ* reaction of **1** with ^{15}N-aniline in methanol-d_4. The spectrum also contains signals of unreacted starting substances.

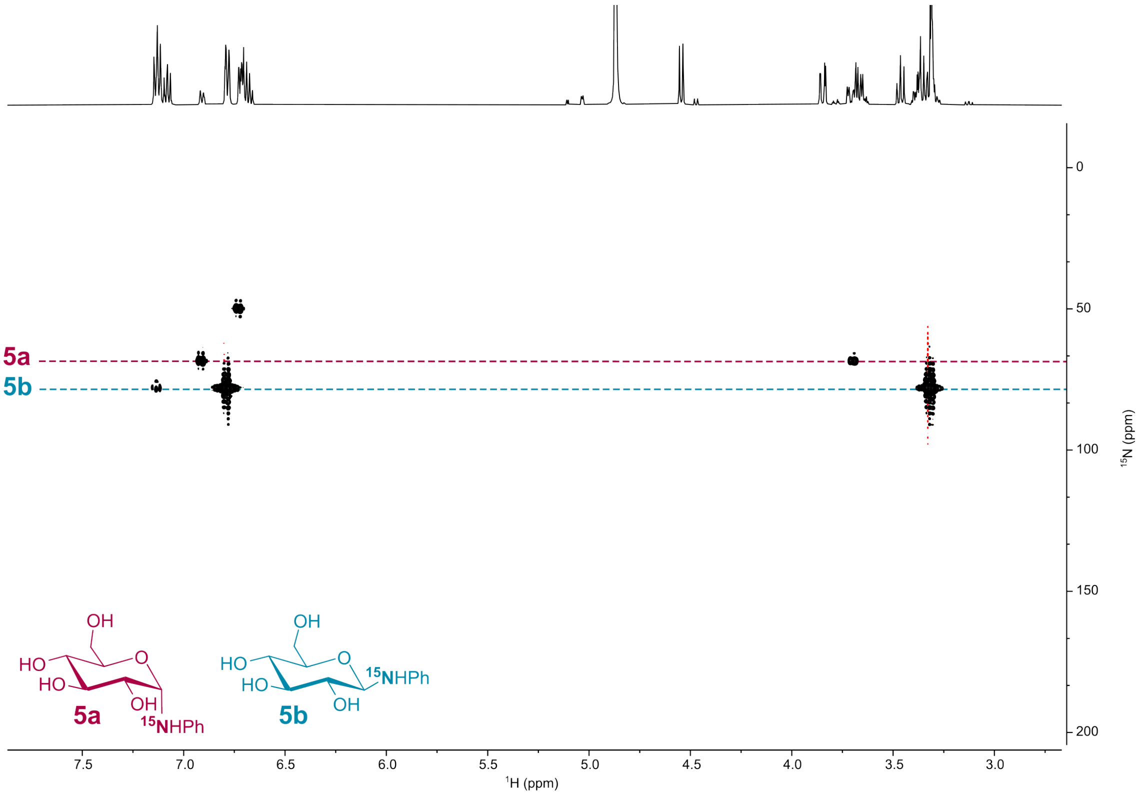

Figure S8: ^{1}H-^{15}N HMBC NMR spectrum (298 K) of **5a** and **5b** as products of the *in situ* reaction of **1** with ^{15}N-aniline in methanol-d_4. The spectrum also contains signals of unreacted starting substances.

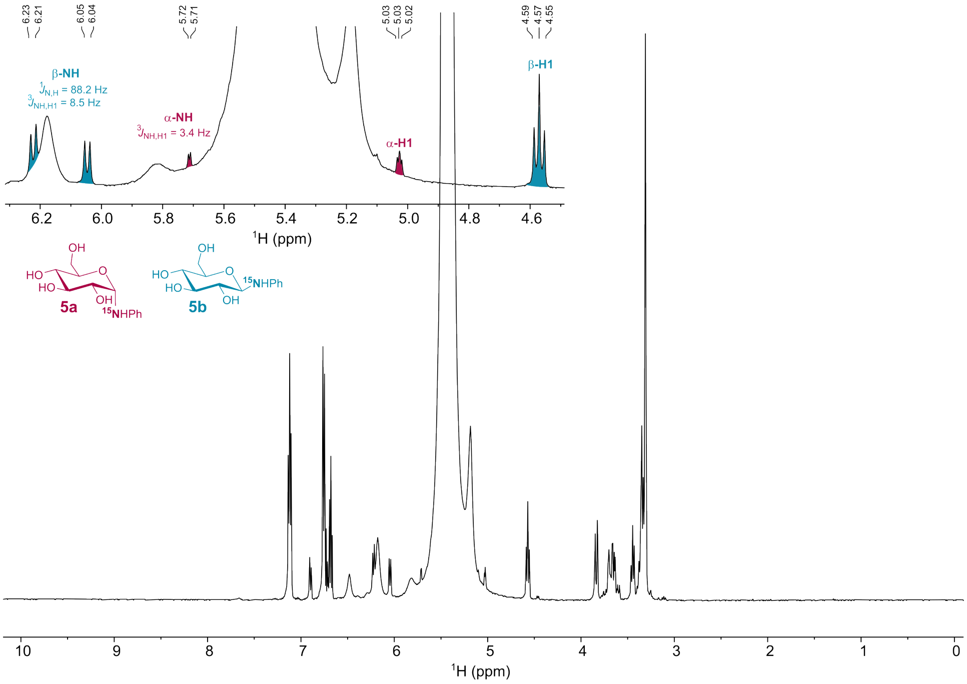

Figure S9: ^{1}H NMR spectrum (500 MHz, 233 K) of **5a** and **5b** as products of the *in situ* reaction of **1** with ^{15}N-aniline in CD_3OH.

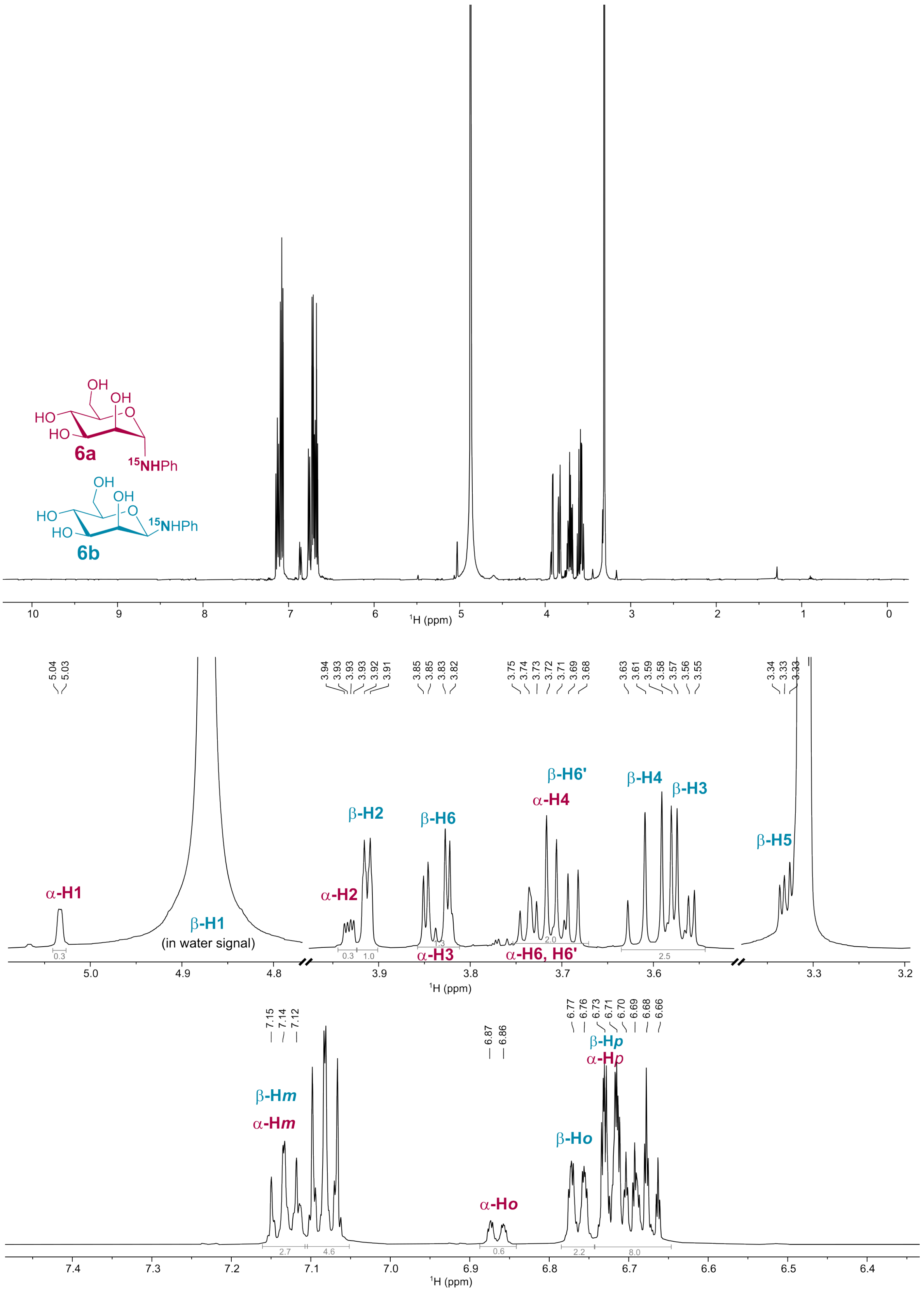

Figure S10: ^{1}H NMR spectrum (600 MHz, 298 K) of **6a** and **6b** as products of the *in situ* reaction of **2** with ^{15}N-aniline in methanol-d_4. The spectrum also contains signals of unreacted starting substances.

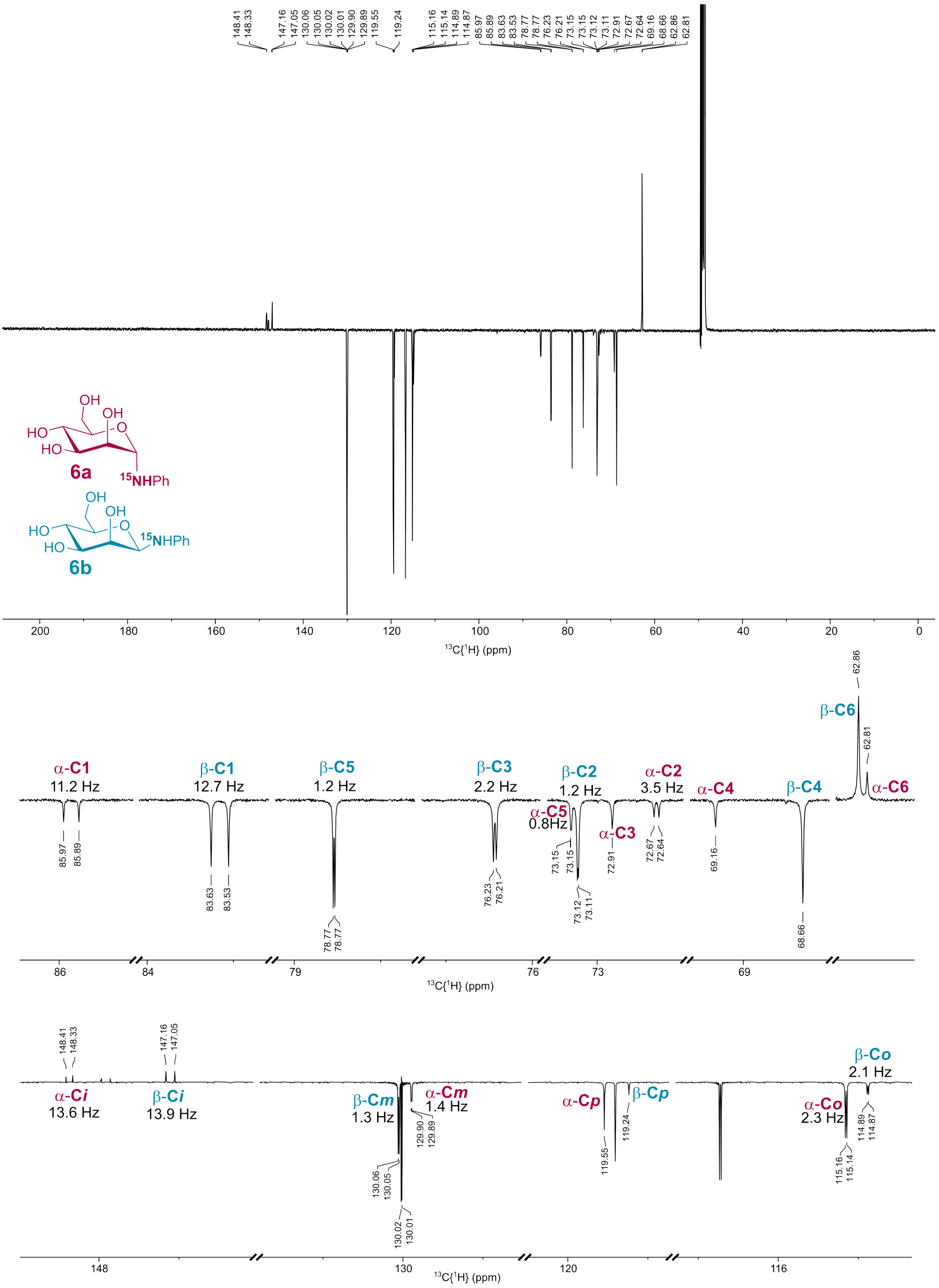

Figure S11: ^{13}C-APT NMR spectrum (151 MHz, 298 K) of **6a** and **6b** as products of the *in situ* reaction of **2** with ^{15}N-aniline in methanol-d_4. The spectrum also contains signals of unreacted starting substances.

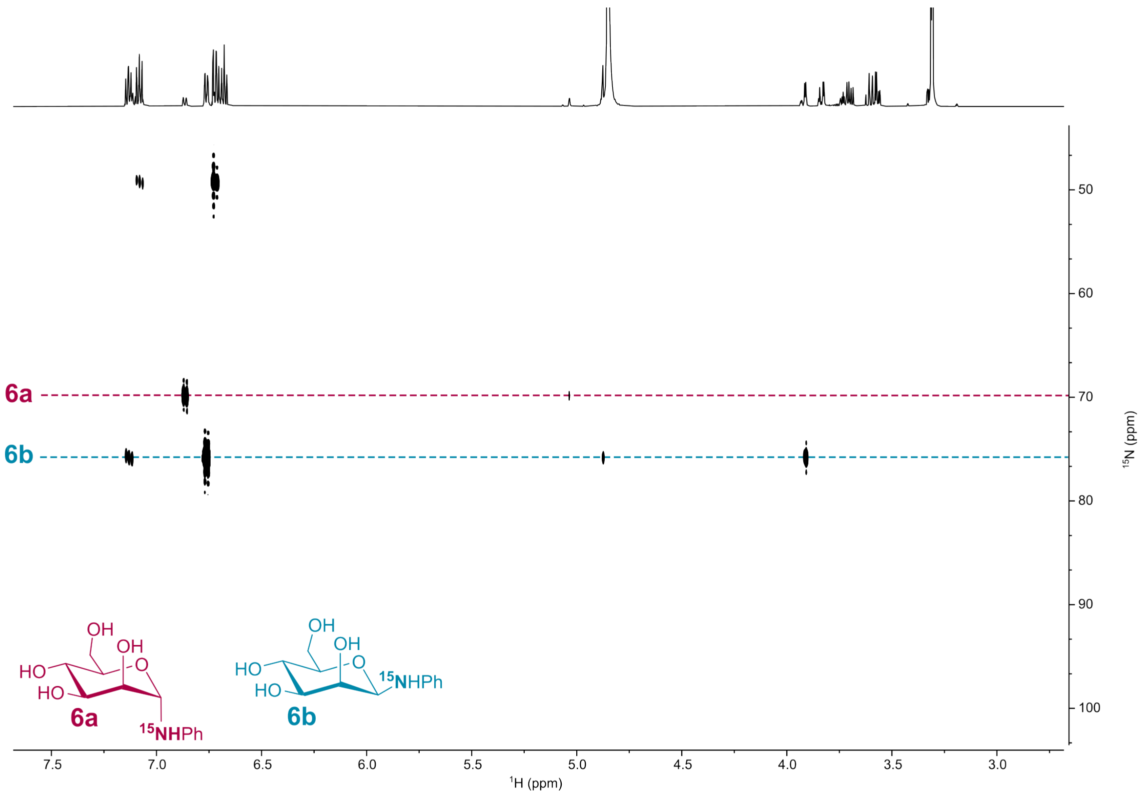

Figure S12: ^{1}H-^{15}N HMBC NMR spectrum (298 K) of **6a** and **6b** as products of the *in situ* reaction of **2** with ^{15}N-aniline in methanol-d_4. The spectrum also contains signals of unreacted starting substances.

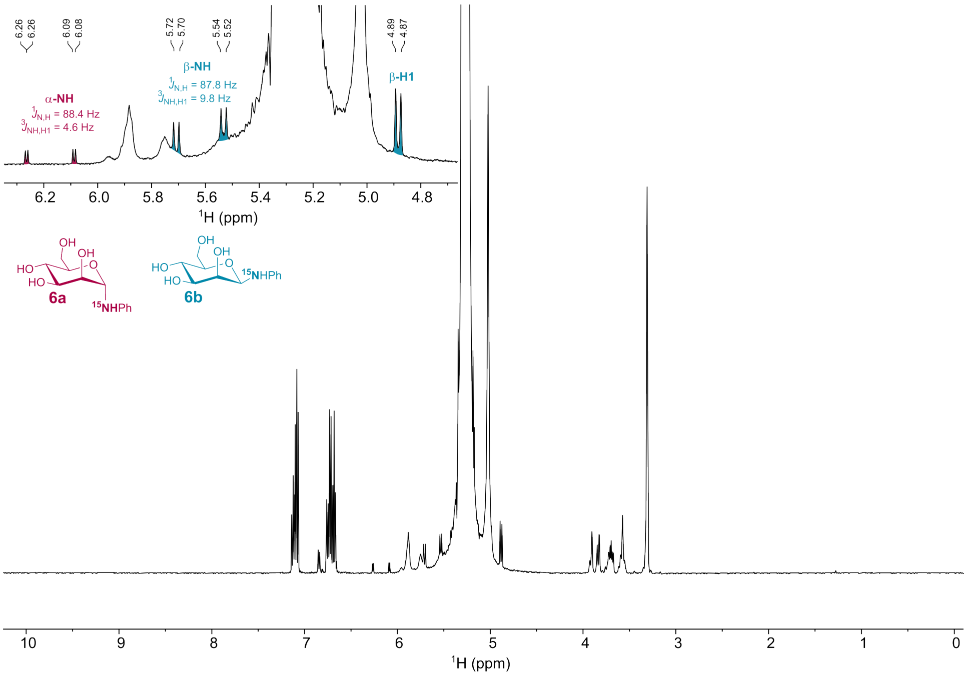

Figure S13: ^{1}H NMR spectrum (500 MHz, 253 K) of **6a** and **6b** as products of the *in situ* reaction of **2** with ^{15}N-aniline in CD_3OH.

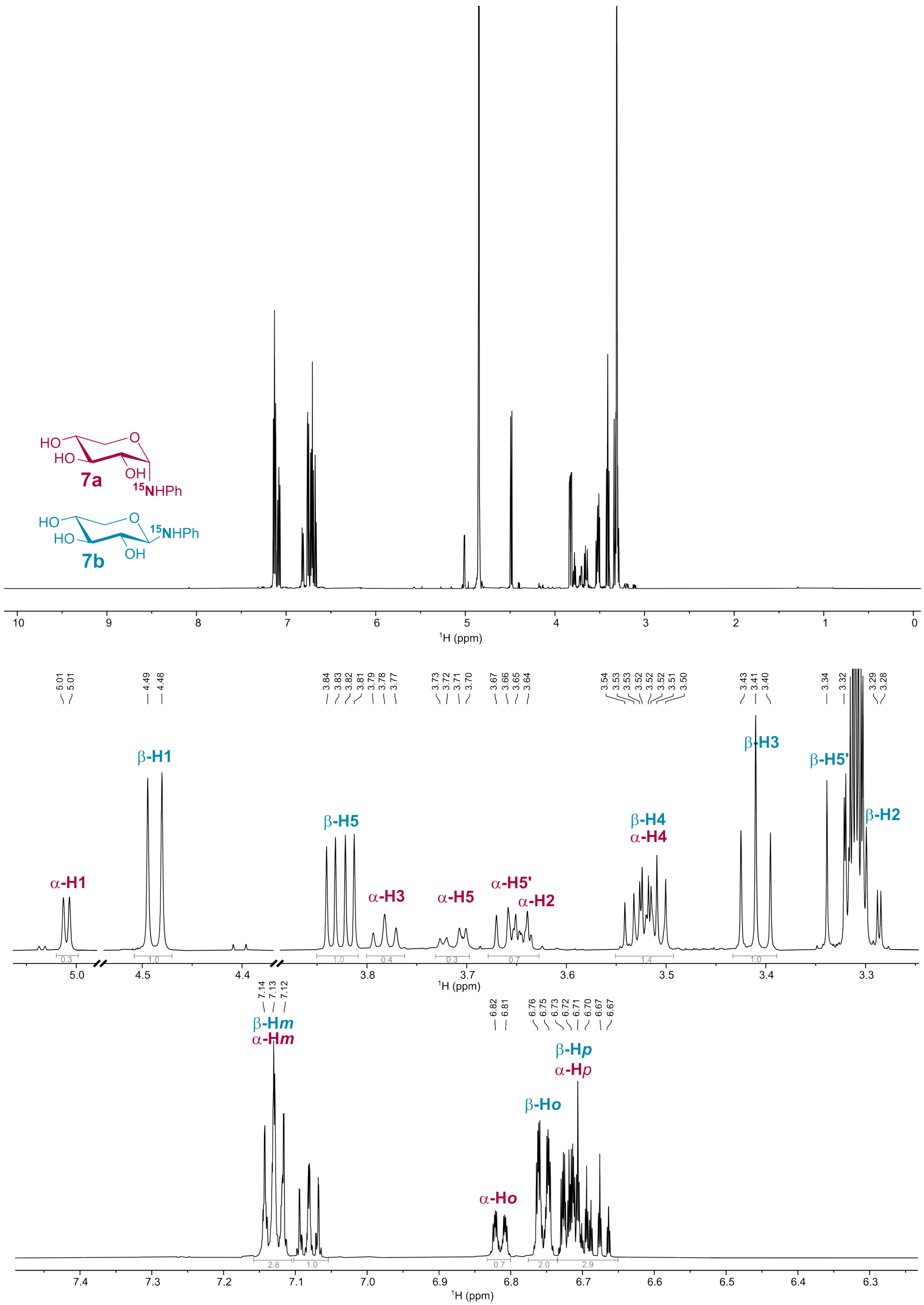

Figure S14: ^{1}H NMR spectrum (600 MHz, 298 K) of **7a** and **7b** as products of the *in situ* reaction of **3** with ^{15}N-aniline in methanol-d_4. The spectrum also contains signals of unreacted starting substances.

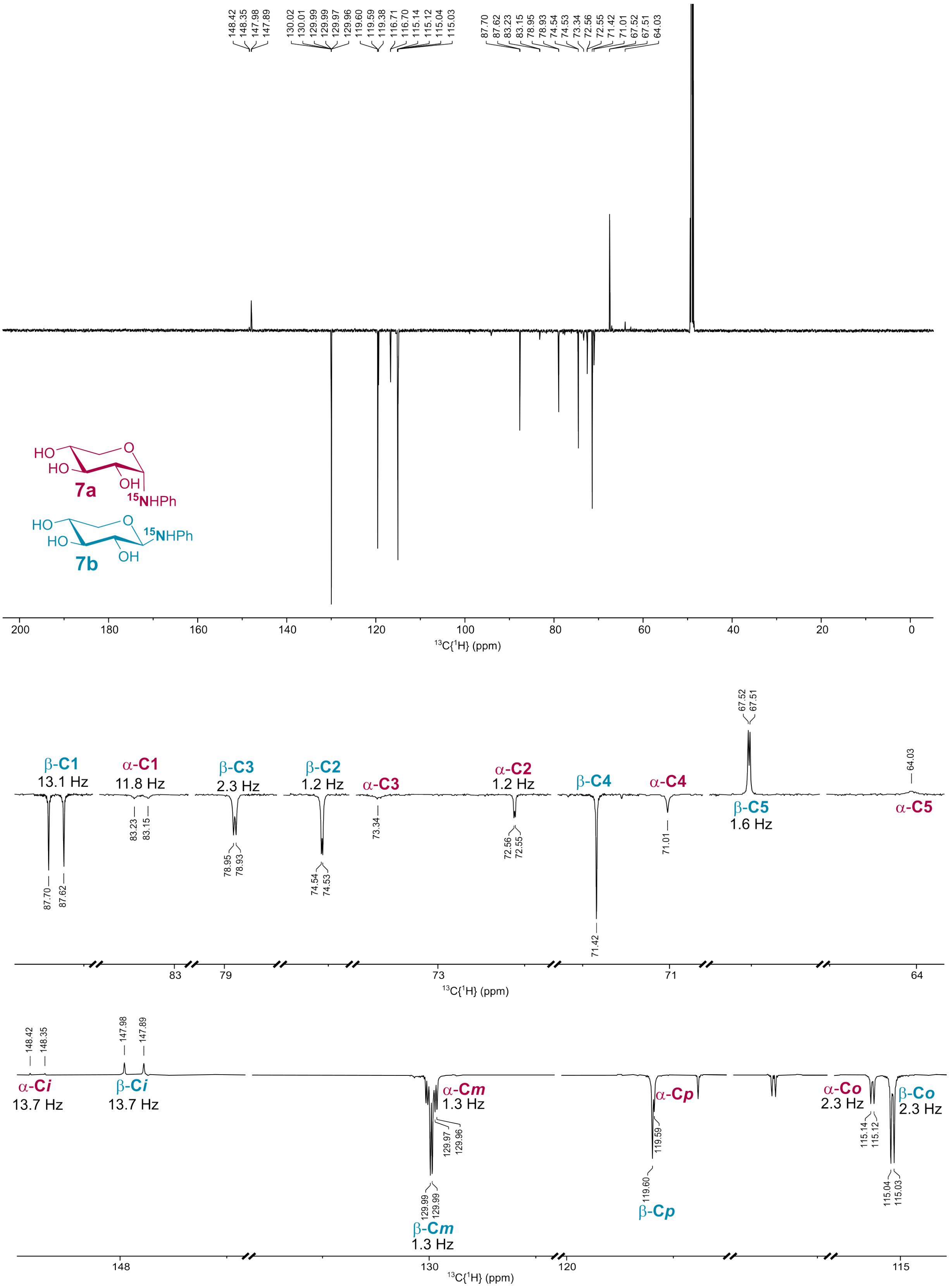

Figure S15: ^{13}C-APT NMR spectrum (151 MHz, 298 K) of **7a** and **7b** as products of the *in situ* reaction of **3** with ^{15}N-aniline in methanol-d_4. The spectrum also contains signals of unreacted starting substances.

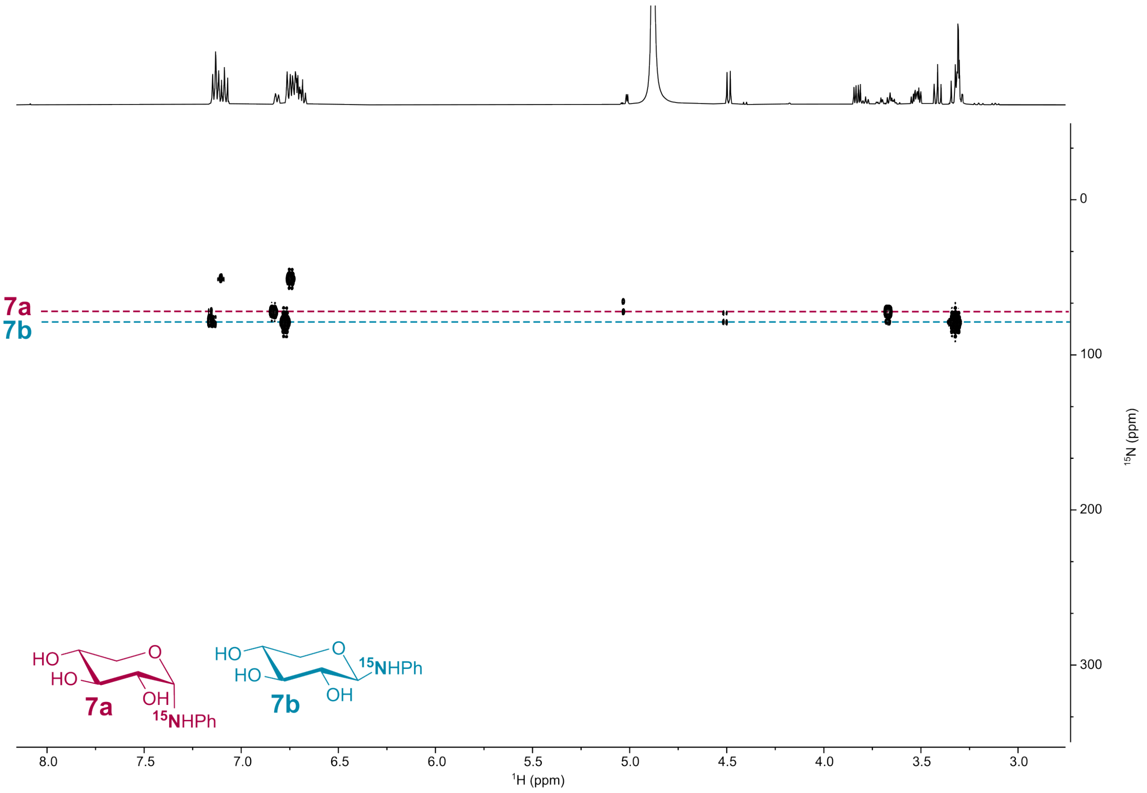

Figure S16: ^{1}H-^{15}N HMBC NMR spectrum (600 MHz, 298 K) of **7a** and **7b** as products of the *in situ* reaction of **3** with ^{15}N-aniline in methanol-d_4. The spectrum also contains signals of unreacted starting substances.

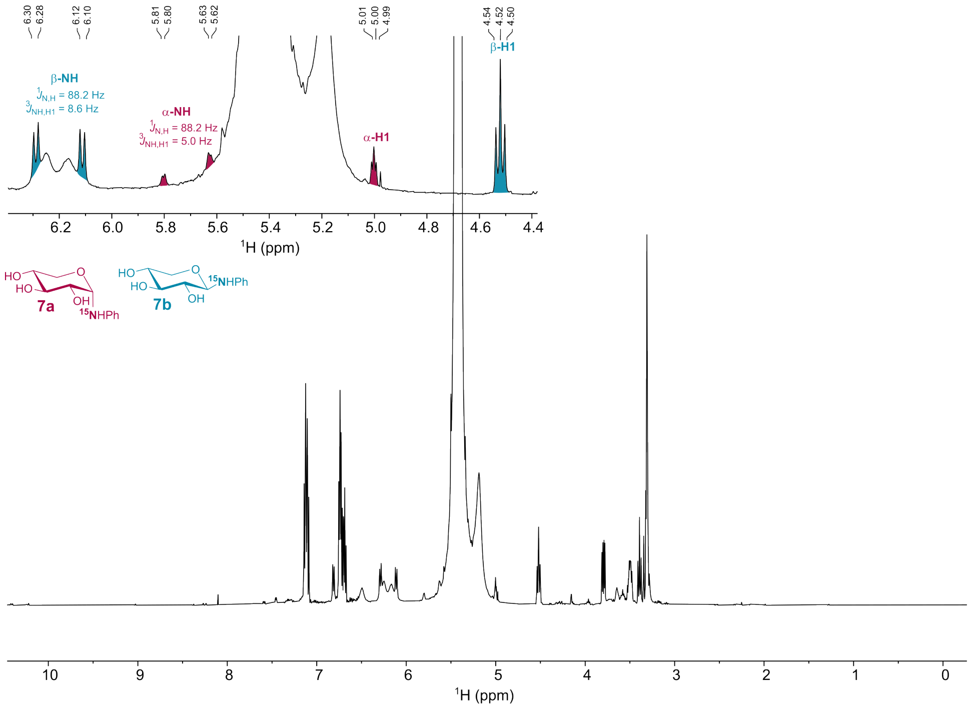

Figure S17: ^{1}H NMR spectrum (500 MHz, 233 K) of **7a** and **7b** as products of the *in situ* reaction of **3** with ^{15}N-aniline in CD_3OH.

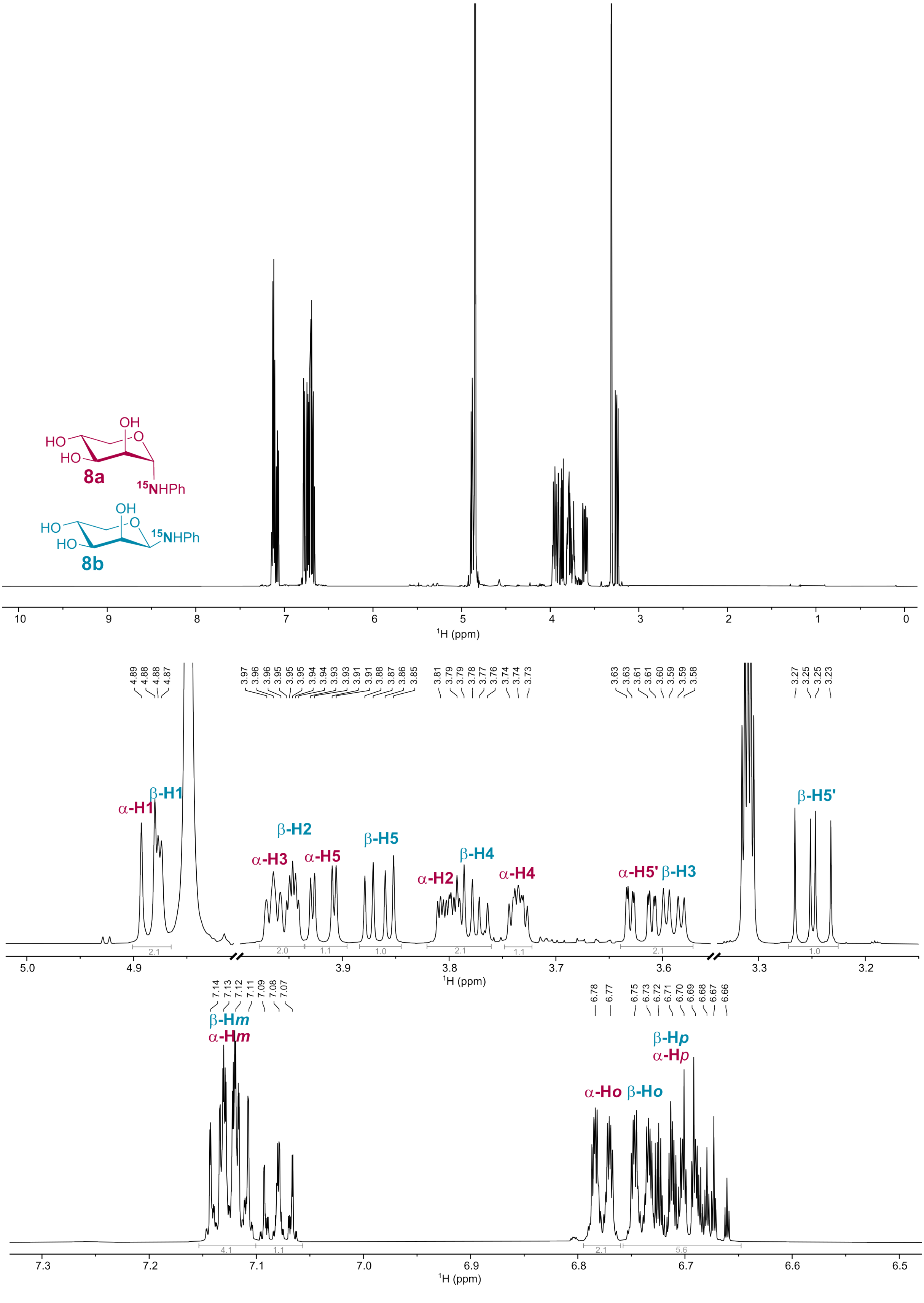

Figure S18: ¹H NMR spectrum (600 MHz, 298 K) of **8a** and **8b** as products of the *in situ* reaction of **4** with ¹⁵N-aniline in methanol-d_4. The spectrum also contains signals of unreacted starting substances.

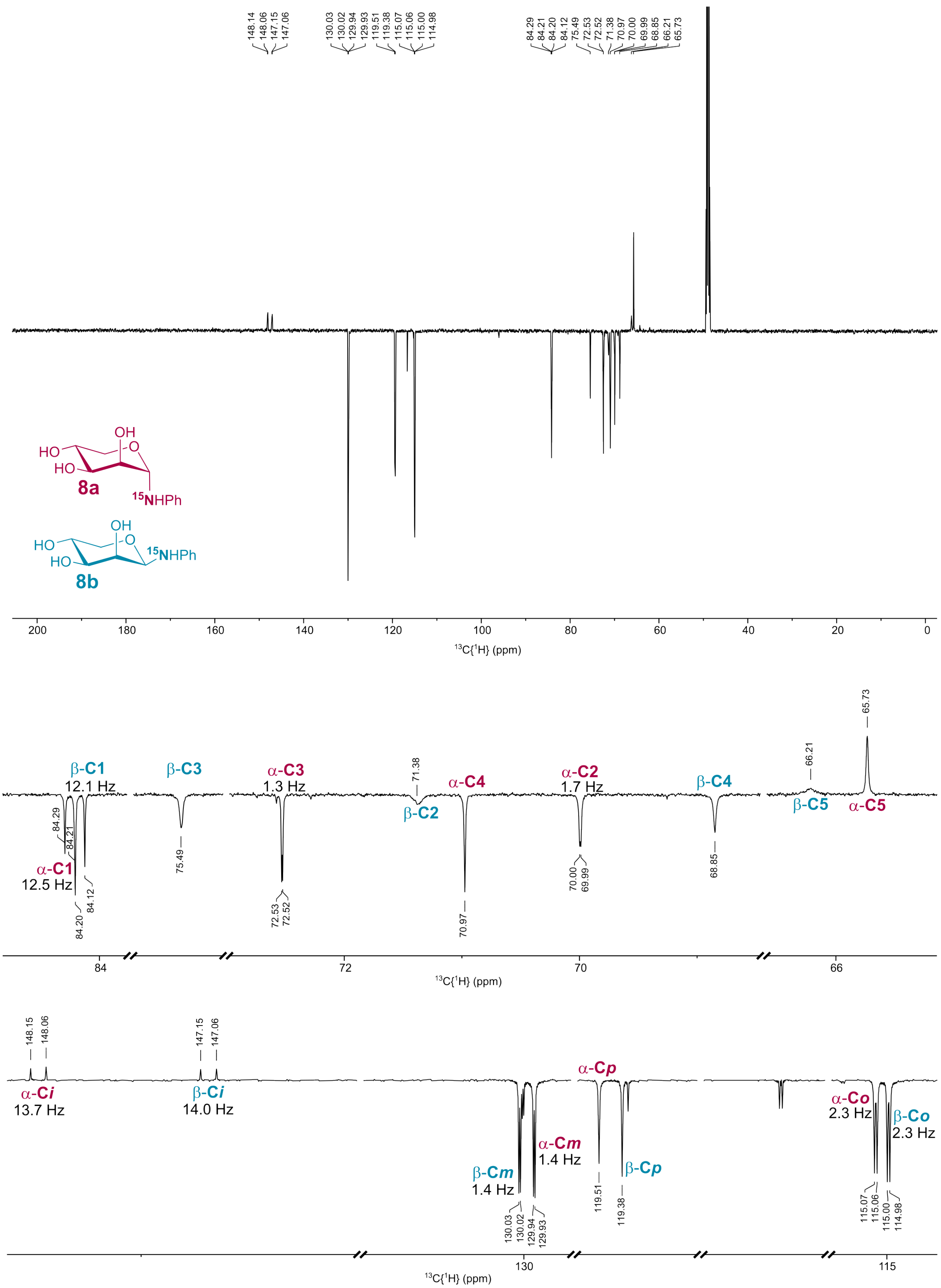

Figure S19: ^{13}C-APT NMR spectrum (151 MHz, 298 K) of **8a** and **8b** as products of the *in situ* reaction of **4** with ^{15}N-aniline in methanol-d_4. The spectrum also contains signals of unreacted starting substances.

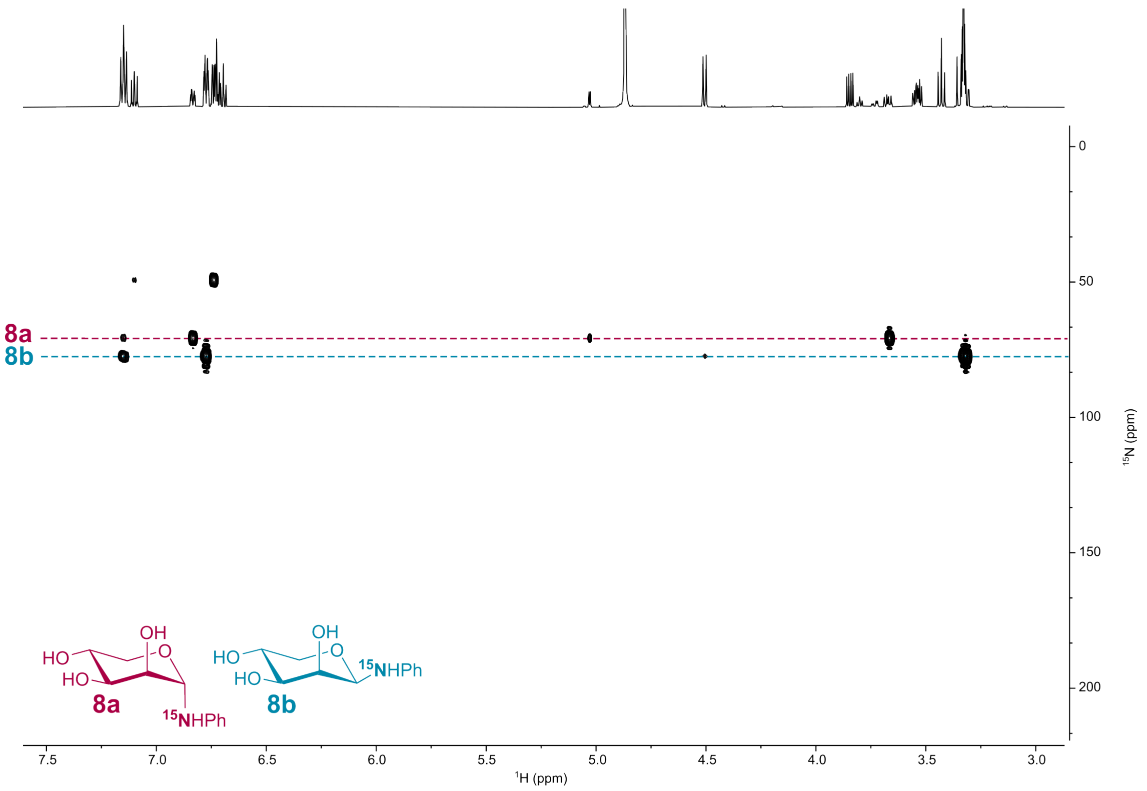

Figure S20: ^{1}H-^{15}N HMBC NMR spectrum (298 K) of **8a** and **8b** as products of the *in situ* reaction of **4** with ^{15}N-aniline in methanol-d_4. The spectrum also contains signals of unreacted starting substances.

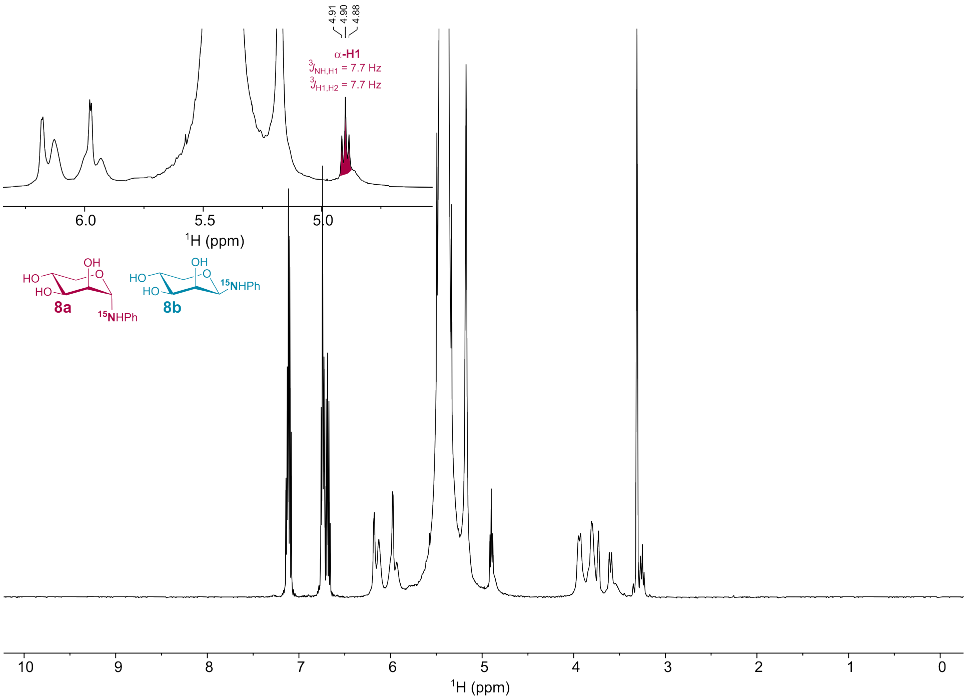

Figure S21: ^{1}H NMR spectrum (500 MHz, 233 K) of **8a** and **8b** as products of the *in situ* reaction of **4** with ^{15}N-aniline in CD_3OH.

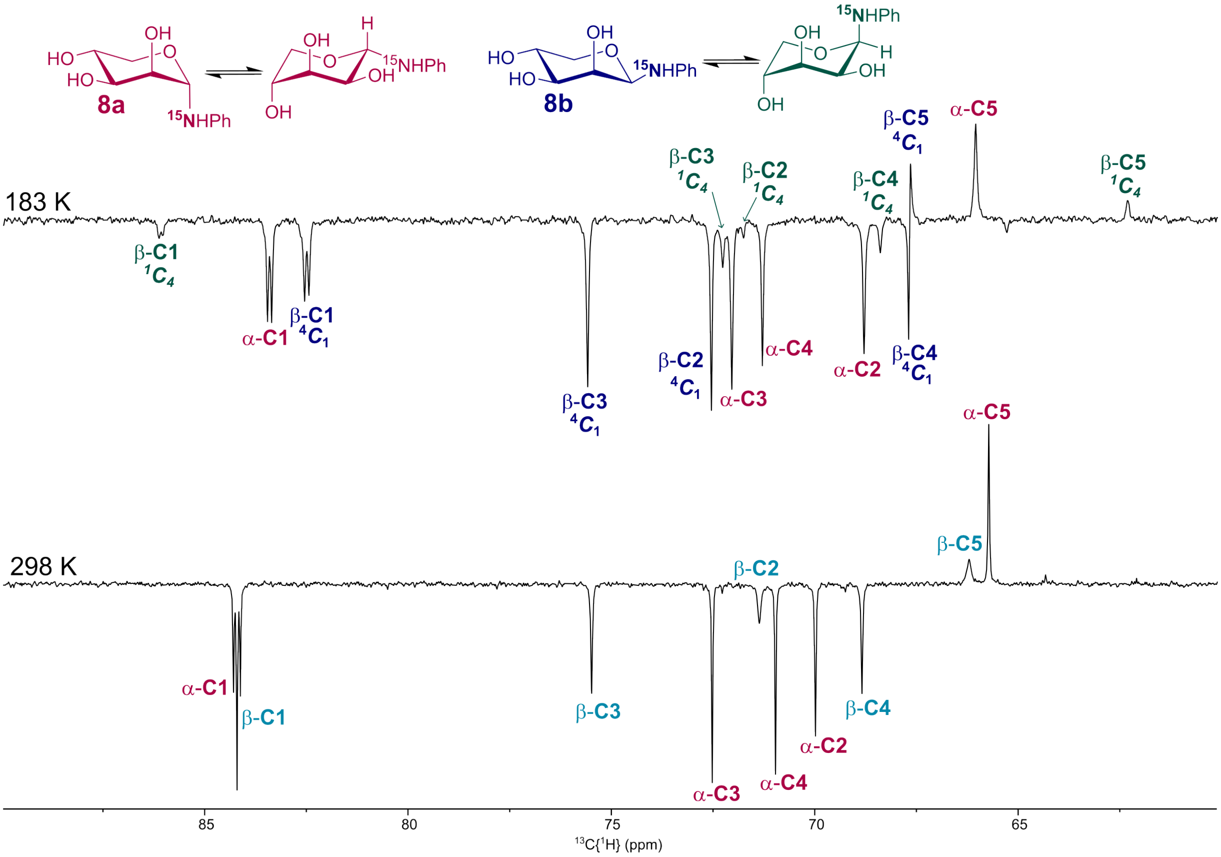

Figure S22: ^{13}C-APT NMR spectrum of **8a** and **8b** at 298 K and 183 K in methanol-d_4.

2.3 NMR spectra of unlabeled *N*-phenyl-glycosylamines

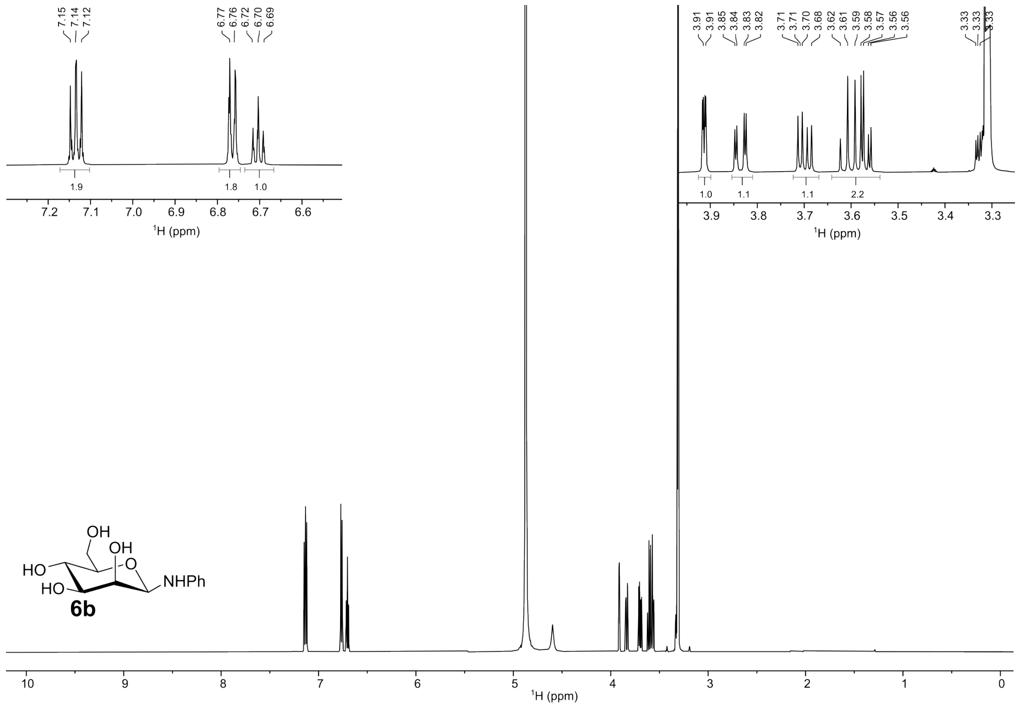

Figure S23: ^{1}H NMR spectrum (600 MHz, 298 K) of unlabeled *N*-phenyl β-D-mannopyranosylamine (**6b**) in methanol-d_4.

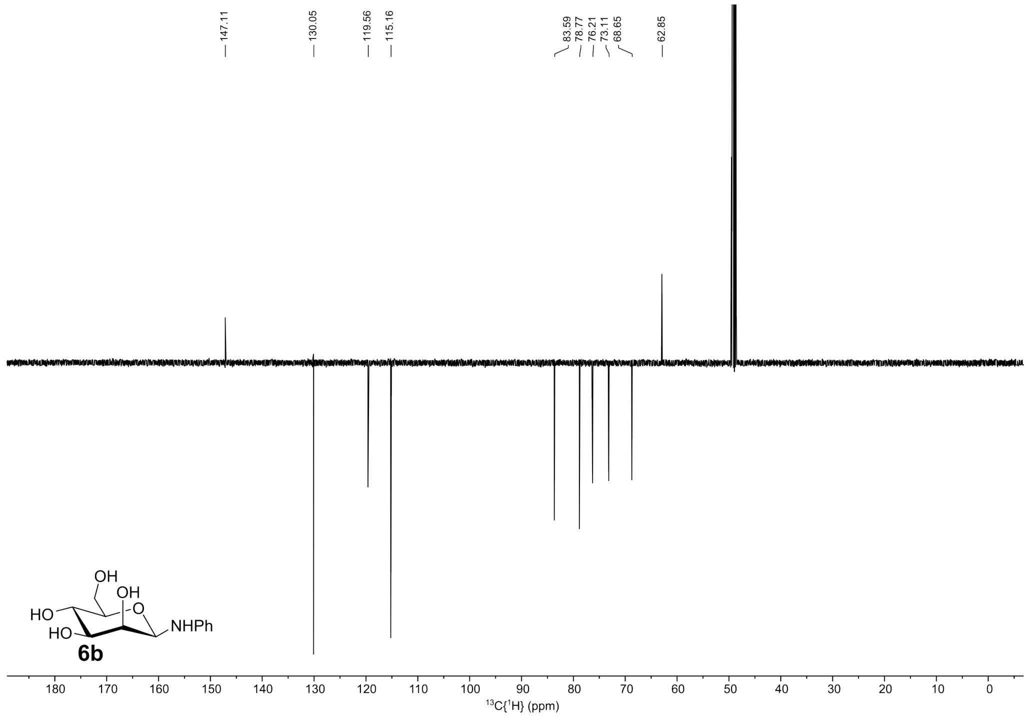

Figure S24: ^{13}C-APT NMR spectrum (151 MHz, 298 K) of unlabeled *N*-phenyl β-D-mannopyranosylamine (**6b**) in methanol-d_4.

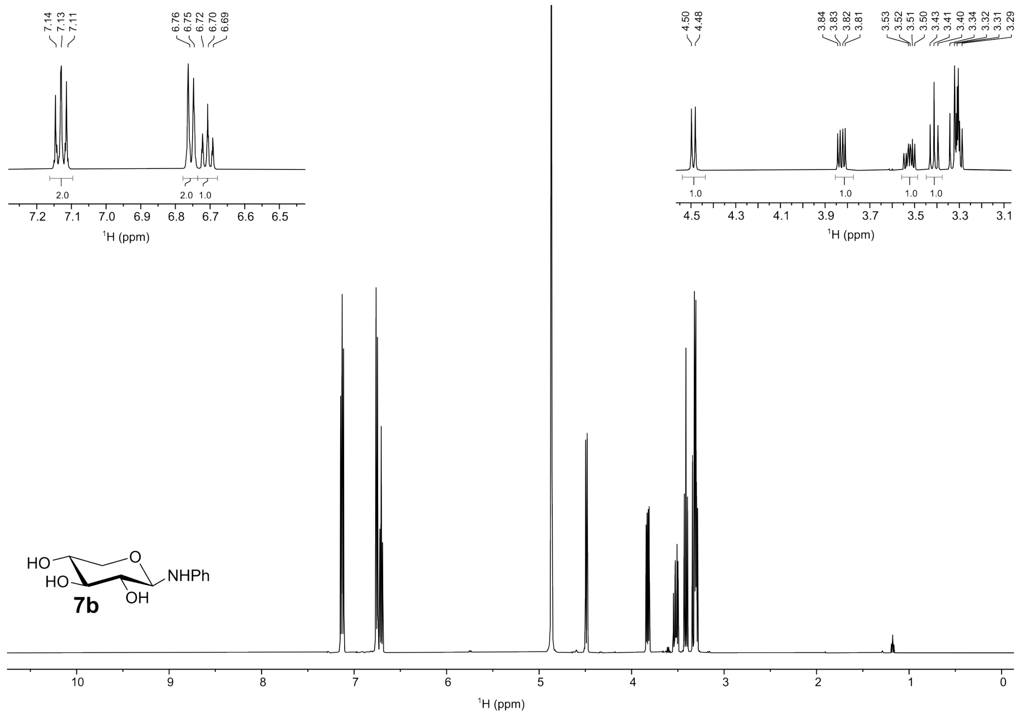

Figure S25: ^{1}H NMR spectrum (500 MHz, 298 K) of unlabeled *N*-phenyl β-D-xylopyranosylamine (**7b**) in methanol-d_4.

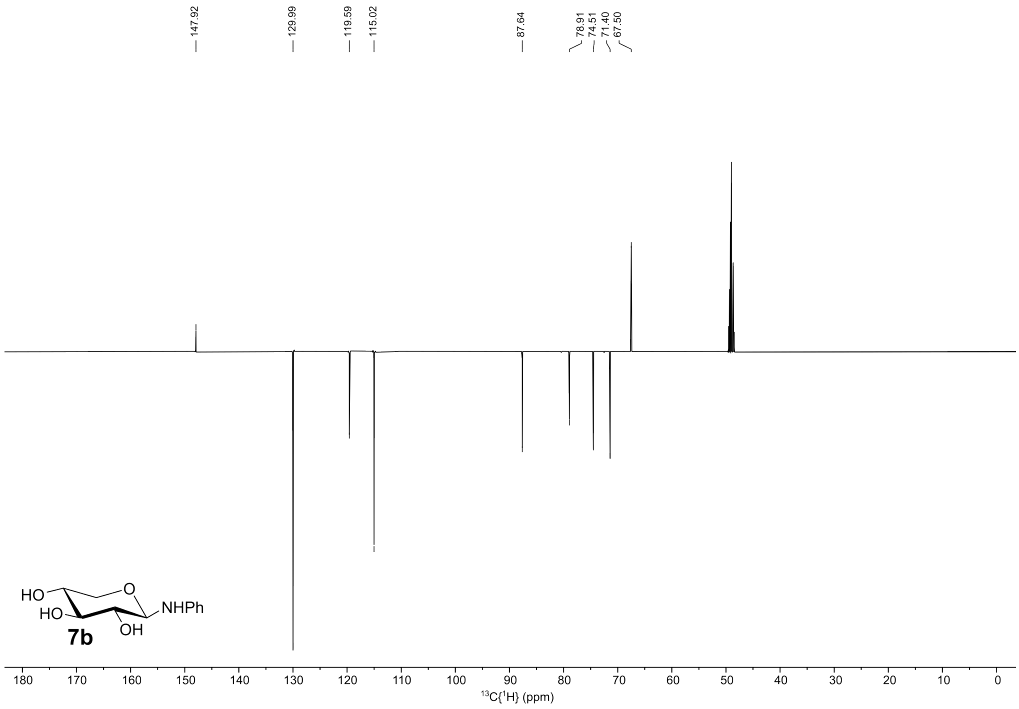

Figure S26: ^{13}C-APT NMR spectrum (125.7 MHz, 298 K) of unlabeled *N*-phenyl β-D-xylopyranosylamine (**7b**) in methanol-d_4.

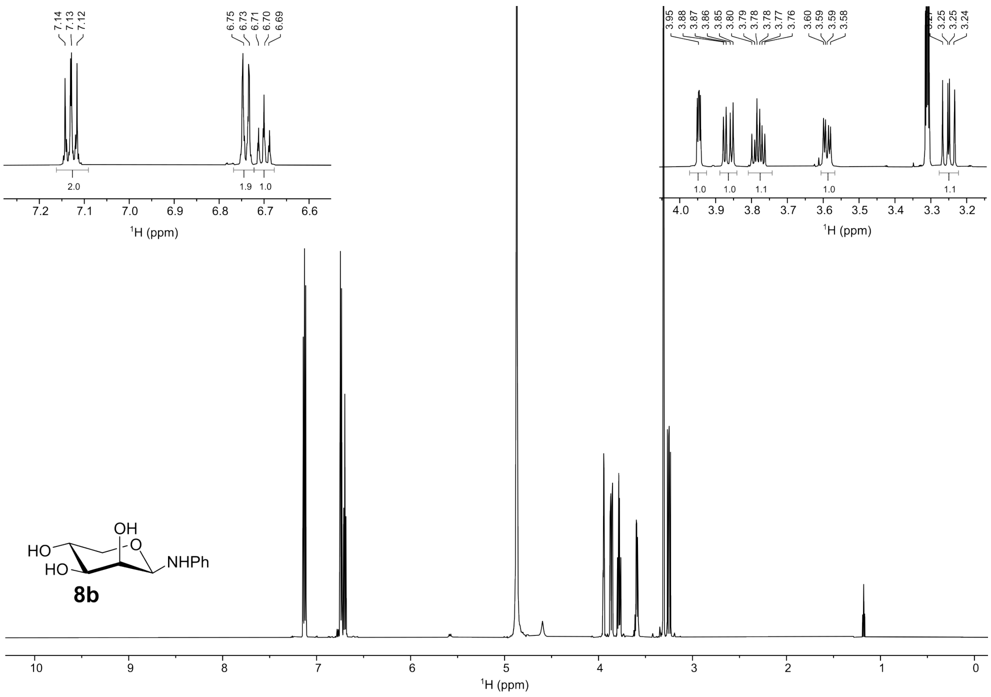

Figure S27: ^{1}H NMR spectrum (600 MHz, 298 K) of unlabeled *N*-phenyl β-D-lyxopyranosylamine (**8b**) in methanol-d_4.

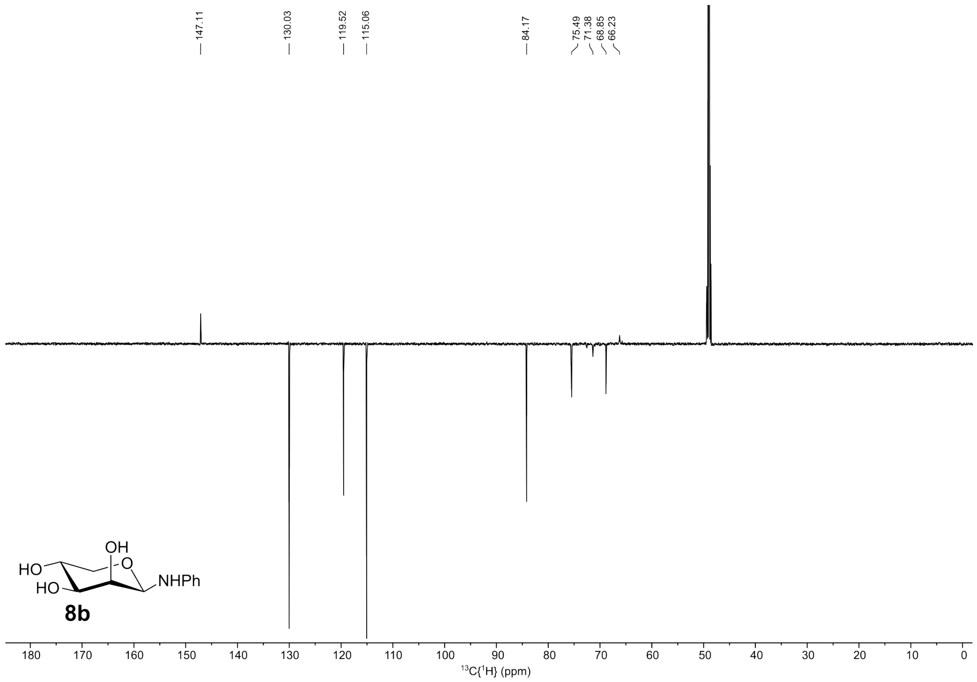

Figure S28: ^{13}C-APT NMR spectrum (151 MHz, 298 K) of unlabeled *N*-phenyl β-D-lyxopyranosylamine (**8b**) in methanol-d_4.

3 Determination of the pyranose ring conformation

Table S5: Experimental, calculated and fitted ring J-coupling constants of **7a** with mean absolute error (MAE) and resulting conformer populations.

J [Hz]	Expt. J	DFT calculation approach $^4C_1$	$^1C_4$	Fitted J	Altona-equation approach $^4C_1$	$^1C_4$	Fitted J
^3J(H1,H2)	3.6	6.65	2.44	4.8	4.72	1.52	3.5
^3J(H2,H3)	6.8	10.04	2.66	6.8	9.68	1.90	6.8
^3J(H3,H4)	6.8	8.41	2.51	5.8	9.24	1.91	6.5
^3J(H4,H5S)	7.1	10.57	1.62	6.6	10.64	1.88	7.4
^3J(H4,H5R)	4.0	6.05	2.30	4.4	5.57	2.08	4.3
Population		56 %	44 %		63 %	37 %	
MAE [Hz]				0.6			0.2

Table S6: Experimental, calculated and fitted ring J-coupling constants of **7b** with mean absolute error (MAE) and resulting conformer populations.

J [Hz]	Expt. J	DFT calculation approach $^4C_1$	$^1C_4$	Fitted J	Altona-equation approach $^4C_1$	$^1C_4$	Fitted J
^3J(H1,H2)	8.5	8.32	1.71	8.3	8.50	1.62	8.2
^3J(H2,H3)	8.7	8.48	3.36	8.5	9.54	2.62	9.2
^3J(H3,H4)	8.9	8.76	3.58	8.8	9.31	2.57	9.0
^3J(H4,H5S)	10.4	10.25	1.97	10.2	10.66	2.15	10.3
^3J(H4,H5R)	5.3	5.97	1.49	6.0	5.46	1.67	5.3
Population		100 %	0 %		96 %	4 %	
MAE [Hz]				0.3			0.2

Table S7: Experimental, calculated and fitted ring J-coupling constants of **8a** with mean absolute error (MAE) and resulting conformer populations.

J [Hz]	Expt. J	DFT calculation approach $^4C_1$	$^1C_4$	Fitted J	Altona-equation approach $^4C_1$	$^1C_4$	Fitted J
^3J(H1,H2)	7.6	1.83	9.18	8.1	1.38	8.54	7.6
^3J(H2,H3)	3.2	3.97	4.38	4.3	3.61	3.30	3.3
^3J(H3,H4)	4.8	8.83	4.07	4.8	9.11	2.96	3.8
^3J(H4,H5S)	3.3	10.58	2.09	3.3	10.64	2.42	3.5
^3J(H4,H5R)	2.2	6.32	1.56	2.2	5.46	1.10	1.7
Population		14 %	86 %		13 %	87 %	
MAE [Hz]				0.3			0.4

Table S8: Experimental, calculated and fitted ring J-coupling constants of **8b** with mean absolute error (MAE) and resulting conformer populations.

J [Hz]	Expt. J	DFT calculation approach $^4C_1$	$^1C_4$	Fitted J	Altona-equation approach $^4C_1$	$^1C_4$	Fitted J
^3J(H1,H2)	2.0	2.01	5.56	2.7	1.51	4.95	2.0
^3J(H2,H3)	3.4	4.27	4.63	4.3	3.79	3.17	3.7
^3J(H3,H4)	8.3	8.68	3.81	7.7	9.05	2.56	8.1
^3J(H4,H5S)	8.7	10.30	2.46	8.7	10.68	1.86	9.4
^3J(H4,H5R)	4.7	5.97	2.34	5.2	5.12	1.98	4.7
Population		80 %	20 %		86 %	14 %	
MAE [Hz]				0.6			0.2

Table S9: Experimental, calculated and fitted ring J-coupling constants of α-**3** with mean absolute error (MAE) and resulting conformer populations.

J [Hz]	Expt. J	DFT calculation approach $^4C_1$	$^1C_4$	Fitted J	Altona-equation approach $^4C_1$	$^1C_4$	Fitted J
^3J(H1,H2)	3.6	4.25	1.77	4.1	3.52	1.31	3.3
^3J(H2,H3)	9.2	9.47	3.64	9.1	9.92	2.66	3.3
^3J(H3,H4)	8.6	8.69	3.31	8.3	9.27	2.71	8.7
^3J(H4,H5S)	10.2	10.78	1.89	10.2	10.64	2.11	10.0
^3J(H4,H5R)	5.4	6.30	1.81	6.0	5.74	1.53	5.4
Population		94 %	6 %		92 %	8 %	
MAE [Hz]				0.3			0.2

Table S10: Experimental, calculated and fitted ring J-coupling constants of β-**3** with mean absolute error (MAE) and resulting conformer populations.

J [Hz]	Expt. J	DFT calculation approach $^4C_1$	$^1C_4$	Fitted J	Altona-equation approach $^4C_1$	$^1C_4$	Fitted J
^3J(H1,H2)	7.6	7.39	1.80	7.4	7.76	2.15	7.5
^3J(H2,H3)	9.1	9.13	3.44	9.1	9.78	2.60	9.4
^3J(H3,H4)	8.7	8.65	3.72	8.7	9.24	2.67	8.9
^3J(H4,H5S)	10.4	10.32	1.82	10.3	10.65	1.93	10.2
^3J(H4,H5R)	5.4	6.10	2.09	6.1	5.57	1.89	5.4
Population		100 %	0 %		95 %	5 %	
MAE [Hz]				0.3			0.2

Table S11: Experimental, calculated and fitted ring J-coupling constants of α-**4** with mean absolute error (MAE) and resulting conformer populations.

J [Hz]	Expt. J	DFT calculation approach $^4C_1$	$^1C_4$	Fitted J	Altona-equation approach $^4C_1$	$^1C_4$	Fitted J
^3J(H1,H2)	3.7	1.63	7.71	3.7	1.76	7.70	3.7
^3J(H2,H3)	2.8	4.28	3.99	4.2	3.79	3.50	3.8
Population		66 %	34 %		67 %	33 %	
MAE [Hz]				0.7			0.4

Table S12: Experimental, calculated and fitted ring J-coupling constants of β-**4** with mean absolute error (MAE) and resulting conformer populations.

J [Hz]	Expt. J	DFT calculation approach $^4C_1$	$^1C_4$	Fitted J	Altona-equation approach $^4C_1$	$^1C_4$	Fitted J
^3J(H1,H2)	2.1	1.67	3.82	2.5	1.61	3.09	2.2
^3J(H2,H3)	3.3	4.02	3.96	4.0	3.73	2.59	3.3
^3J(H3,H4)	7.2	8.60	4.17	6.8	9.01	2.57	6.3
^3J(H4,H5S)	6.9	10.39	1.59	6.9	10.67	1.83	7.0
^3J(H4,H5R)	4.0	6.25	2.46	4.0	5.39	2.03	4.0
Population		60 %	40 %		59 %	41 %	
MAE [Hz]				0.4			0.2

4 Determination of the anomeric configuration

Table S13: Energy contributions related to the reverse anomeric effect, study of the preferred anomeric configuration

	conformer	E_r	av. p	hc	av. hc	$LP_{O5} - \sigma^*_{(C1-N1)}$	av. $LP_{O5} - \sigma^*_{(C1-N1)}$	$LP_{N1} - \sigma^*_{(O5-C1)}$	av. $LP_{N1} - \sigma^*_{(O5-C1)}$	μ	av.μ
**5a**	(*S*)-(*trans*)	6.5	0.12	-910.9	-916.8	9.3	8.4	3.4	11.3	4.6	5.9
	(*R*)-(−*gauche*)	8.0		-918.3		11.6		14.3		3.6	
	(*R*)-(+*gauche*)	1.3		-914.4		8.1		9.1		6.8	
	(*S*)-(+*gauche*)	1.9		-923.3		9.2		17.1		3.6	
	(*R*)-(−*gauche*)	11.9		-919.8		10.9		4.8		4.9	
**5b**	(*R*)-(+*gauche*)	2.4	0.88	-920.4	-917.3	2.4	2.4	10.9	15.5	5.5	4.7
	(*R*)-(−*gauche*)	0.0		-917.4		2.4		16.0		4.7	
	(*S*)-(+*gauche*)	2.4		-918.9		2.3		15.7		4.4	
	(*S*)-(−*gauche*)	1.4		-916.2		2.4		11.1		4.4	
	conformer	E_r	av. p	hc	av. hc	$LP_{O5} - \sigma^*_{(C1-N1)}$	av. $LP_{O5} - \sigma^*_{(C1-N1)}$	$LP_{N1} - \sigma^*_{(O5-C1)}$	av. $LP_{N1} - \sigma^*_{(O5-C1)}$	μ	av.μ
**6a**	(*S*)-(*trans*)	4.8	0.14	-905.2	-921.1	9.5	8.7	0.0	14.8	2.8	4.0
	(*R*)-(−*gauche*)	7.9		-913.0		10.1		15.8		2.2	
	(*R*)-(+*gauche*)	1.6		-917.6		8.4		11.4		6.8	
	(*S*)-(+*gauche*)	1.4		-923.5		8.8		17.2		4.1	
**6b**	(*R*)-(*trans*)	4.2	0.86	-907.7	-919.6	3.0	2.5	1.1	16.7	4.7	7.0
	(*R*)-(−*gauche*)	0.0		-919.6		2.5		16.7		7.0	
	(*S*)-(+*gauche*)	6.8		-924.1		2.5		8.3		7.3	
	(*S*)-(+*gauche*)	6.5		-922.4		2.4		12.8		3.9	

E_r - Relative energy (in kcal/mol) calculated within α- and β-anomers; av. p - Boltzmann populations for all considered α- and β-conformers estimated from the B3LYP/aug-cc-pVTZ Gibbs energies; hc - total energy of hyperconjugation (in kcal/mol) estimated as the energy difference between the B3LYP/6-311++G** energy and the energy of a perfectly localized system with all doubly occupied Lewis natural bond orbitals (the more negative, the more stabilized; see Methods for details); av. hc - Boltzmann averaged total energy hyperconjugation for individual anomers; $LP_{O5} - \sigma^*_{(C1-N1)}$ and $LP_{N1} - \sigma^*_{(O5-C1)}$ - individual hyperconjugative interactions (in kcal/mol); av. $LP_{O5} - \sigma^*_{(C1-N1)}$ and av. $LP_{N1} - \sigma^*_{(O5-C1)}$ - Boltzmann averaged hyperconjugative interactions for individual anomers; μ - dipole moment (in Debye); av.μ - Boltzmann averaged dipole moment for individual anomers.

5 Determination of the aglycone conformation

5.1 Well-tempered metadynamics simmulations

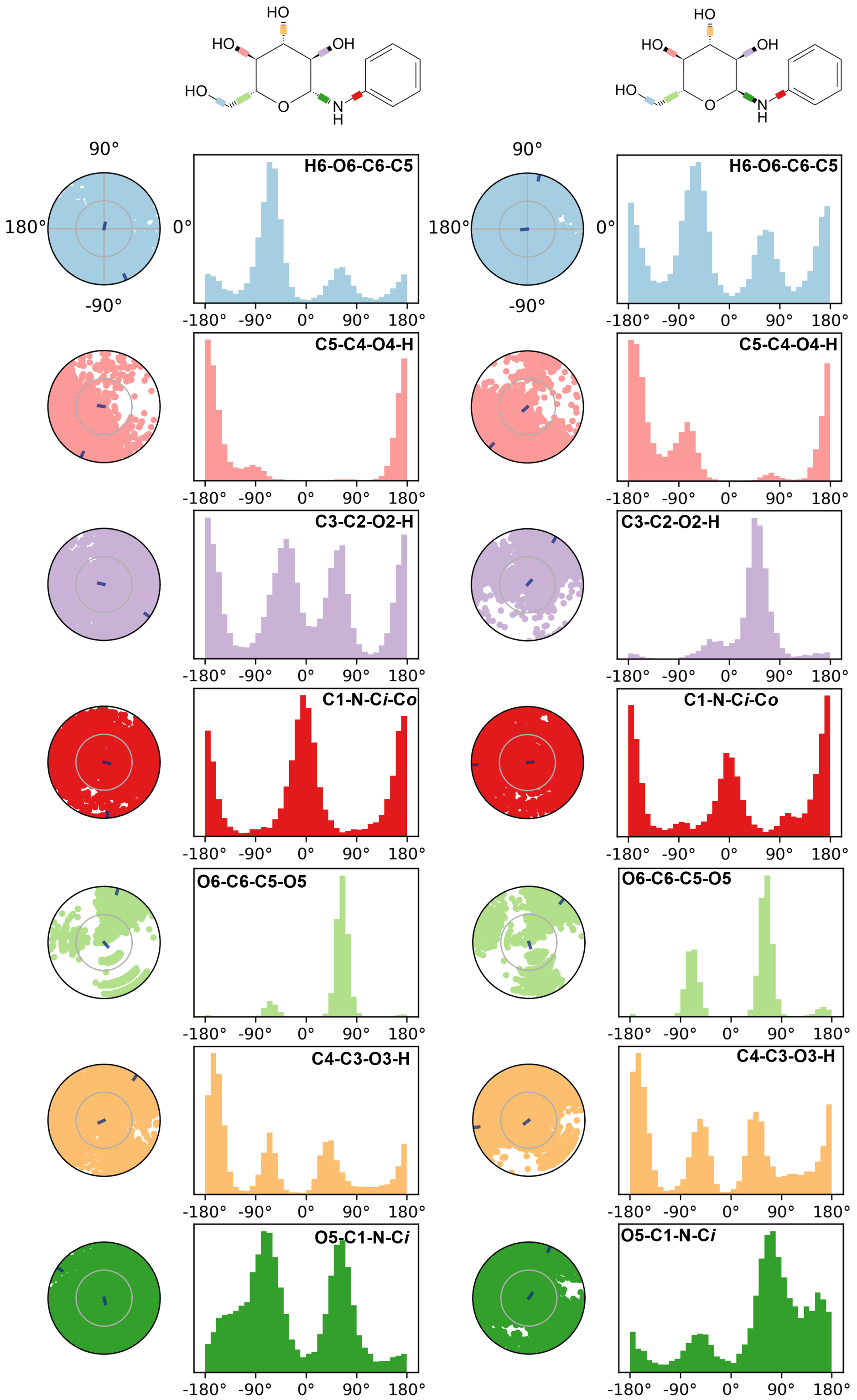

Figure S29: Torsion profile of compound **5b** (left) and **5a** (right) extracted from WTMtD simulations. The radial plots present the ligand's conformational evolution during the simulation, with the start of the simulation depicted at the center and the end represented at the outer edge of the plot. The bar plots illustrate the population distribution of each torsion angle.

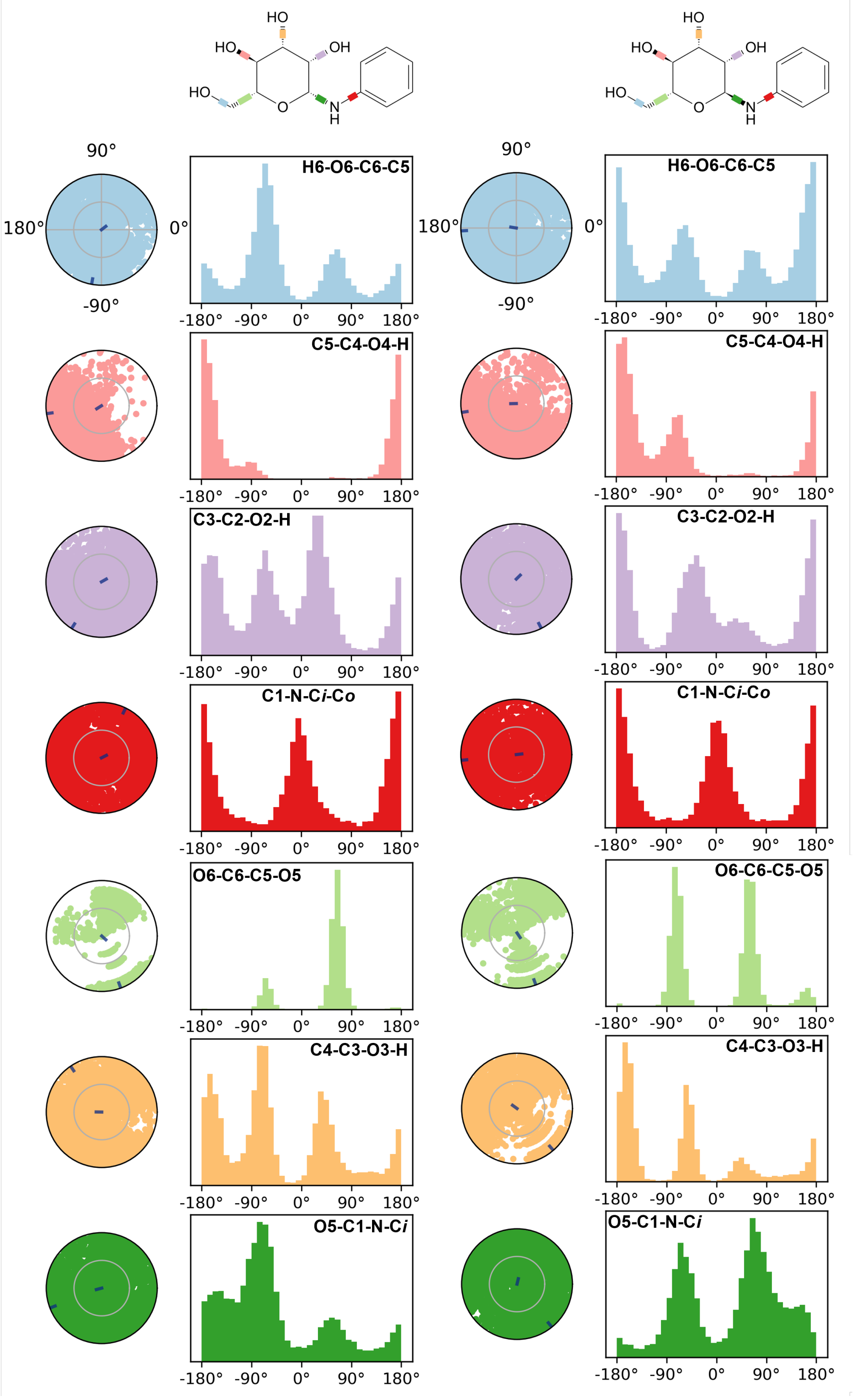

Figure S30: Torsion profile of compound **6b** (left) and **6a** (right) extracted from WTMtD simulations. The radial plots present the ligand's conformational evolution during the simulation, with the start of the simulation depicted at the center and the end represented at the outer edge of the plot. The bar plots illustrate the population distribution of each torsion angle.

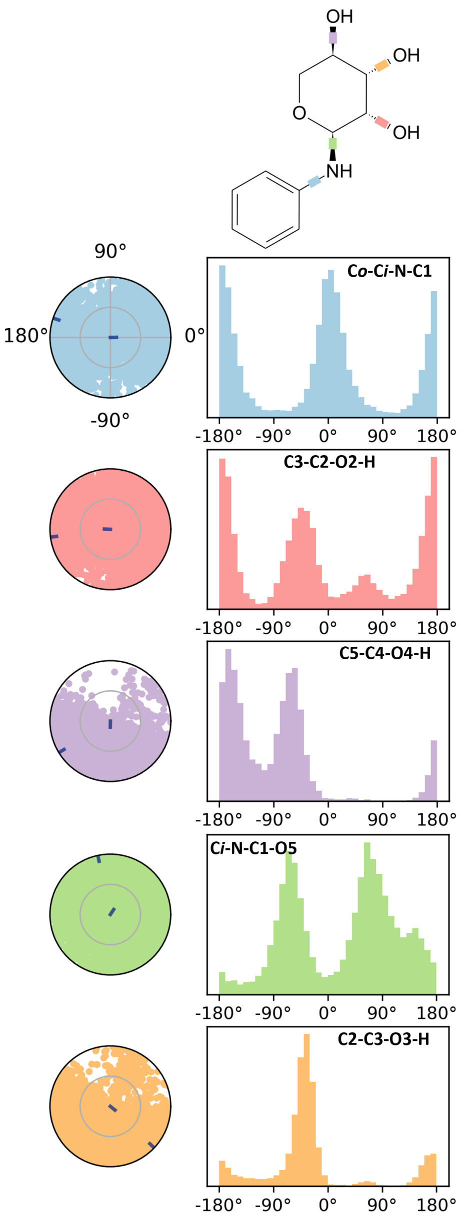

Figure S31: Torsion profile of compound **7b** extracted from WTMtD simulations. The radial plots present the ligand's conformational evolution during the simulation, with the start of the simulation depicted at the center and the end represented at the outer edge of the plot. The bar plots illustrate the population distribution of each torsion angle.

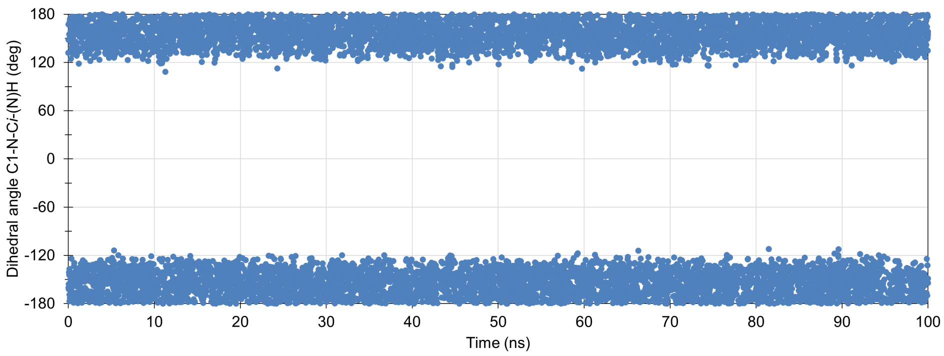

Figure S32: Time evolution of the dihedral angle C1–N–Ci–(N)H for compound **5b** during 100 ns of WTMtD simulation.

5.2 DFT calculations

Table S14: Calculated coupling constants (in Hz) during the gradual rotation of the aglycone (dihedral angle O5-C1-N-C*i* in deg) of compound (*R*)-**5b**. During the calculation, the configuration at the nitrogen atom was fixed.

	Coupling constant [Hz]										
Dih. [deg]	^3J(NH,H1)	^2J(^{15}N,H1)	^3J(^{15}N,H2)	^4J(^{15}N,H3)	^5J(^{15}N,H4)	^4J(^{15}N,H5)	^1J(^{15}N,C1)	^2J(^{15}N,C2)	^3J(^{15}N,C3)	^4J(^{15}N,C4)	^3J(^{15}N,C5)
−180	1.80	0.61	−1.52	0.14	−0.04	0.28	−8.00	−4.67	−1.92	0.14	−2.14
−160	−0.42	0.71	−1.35	0.17	−0.03	0.21	−6.69	−5.14	−2.38	0.12	−1.56
−140	0.17	0.54	−1.46	0.17	−0.03	0.13	−5.63	−5.13	−2.77	0.16	−1.01
−120	3.46	0.27	−1.73	0.15	−0.03	0.08	−4.58	−4.73	−2.99	0.18	−0.59
−100	8.24	−0.28	−2.05	0.12	−0.02	0.07	−3.80	−4.04	−2.96	0.19	−0.37
−80	12.41	−1.28	−2.31	0.11	−0.01	0.08	−3.52	−3.14	−2.78	0.19	−0.31
−60	14.07	−2.50	−2.38	0.09	0.00	0.08	−3.93	−2.17	−2.48	0.19	−0.36
−40	12.76	−4.13	−2.25	0.09	0.02	0.06	−3.56	−1.11	−2.06	0.17	−0.50
−20	8.62	−6.07	−1.69	0.09	0.03	0.03	−2.97	−0.04	−1.47	0.10	−0.67
0	3.01	−7.17	−0.86	0.03	0.04	0.04	−2.47	0.38	−0.69	0.06	−0.95
20	0.10	−7.40	−0.77	0.02	0.04	0.08	−3.25	0.38	−0.21	0.05	−1.32
40	0.07	−6.42	−0.95	0.04	0.04	0.14	−4.58	0.35	−0.01	0.05	−1.77
60	2.32	−4.47	−1.28	0.06	0.04	0.16	−6.49	0.55	−0.13	0.05	−2.06
80	5.72	−2.59	−1.37	0.08	0.03	0.22	−8.33	0.65	−0.27	0.09	−2.46
100	9.16	−1.26	−1.36	0.09	0.02	0.28	−9.46	0.62	−0.38	0.14	−2.73
120	11.15	−0.38	−1.32	0.10	0.00	0.32	−9.86	0.31	−0.50	0.16	−2.82
140	10.52	0.19	−1.20	0.11	−0.01	0.33	−9.66	−0.45	−0.73	0.15	−2.77
160	7.62	0.52	−1.25	0.13	−0.03	0.33	−9.05	−1.91	−1.12	0.16	−2.66
180	3.67	0.59	−1.53	0.14	−0.05	0.29	−8.16	−3.89	−1.73	0.18	−2.31
$max - min$	14.50	8.11	1.61	0.15	0.09	0.30	7.39	5.79	2.98	0.15	2.52

Table S15: Calculated coupling constants (in Hz) during the gradual rotation of the aglycone (dihedral angle O5-C1-N-C*i* in deg) of compound (*S*)-**5b**. During the calculation, the configuration at the nitrogen atom was fixed.

	Coupling constant [Hz]										
Dih. [deg]	^3J(NH,H1)	^2J(^{15}N,H1)	^3J(^{15}N,H2)	^4J(^{15}N,H3)	^5J(^{15}N,H4)	^4J(^{15}N,H5)	^1J(^{15}N,C1)	^2J(^{15}N,C2)	^3J(^{15}N,C3)	^4J(^{15}N,C4)	^3J(^{15}N,C5)
−180	12.66	−3.90	−1.23	0.05	0.03	0.24	−6.77	1.15	0.05	0.04	−2.63
−160	12.27	−1.89	−1.55	0.04	0.02	0.29	−7.84	1.33	0.01	0.11	−2.87
−140	9.09	−0.36	−1.84	0.02	0.01	0.32	−8.65	1.05	−0.18	0.15	−2.87
−120	4.53	0.50	−1.96	0.01	0.00	0.32	−9.27	0.11	−0.51	0.18	−2.67
−100	0.91	0.76	−1.94	0.03	−0.02	0.30	−9.46	−1.12	−0.98	0.22	−2.38
−80	−0.24	0.80	−1.87	0.03	−0.04	0.26	−9.13	−2.29	−1.33	0.24	−1.95
−60	0.71	0.78	−1.92	0.04	−0.04	0.21	−8.42	−3.64	−1.78	0.22	−1.47
−40	3.36	0.50	−1.82	0.05	−0.04	0.16	−6.82	−4.96	−2.28	0.21	−1.10
−20	8.04	0.02	−1.76	0.11	−0.04	0.14	−4.76	−5.15	−2.61	0.24	−0.85
0	10.08	−0.74	−1.65	0.13	−0.02	0.12	−3.57	−4.48	−2.66	0.22	−0.64
20	10.05	−1.48	−1.42	0.15	−0.01	0.11	−3.07	−3.32	−2.54	0.19	−0.51
40	8.42	−2.13	−1.33	0.14	0.00	0.10	−3.05	−2.21	−2.40	0.17	−0.41
60	5.63	−3.03	−1.42	0.14	0.02	0.06	−2.54	−1.43	−2.27	0.15	−0.26
80	1.46	−5.33	−1.52	0.13	0.03	0.04	−1.68	−0.66	−2.26	0.06	−0.15
100	−0.38	−7.15	−1.38	0.10	0.04	0.02	−1.94	0.20	−1.68	−0.02	−0.36
120	1.16	−7.75	−1.24	0.08	0.05	0.05	−2.79	0.65	−1.01	−0.09	−0.85
140	5.43	−7.17	−1.11	0.07	0.04	0.12	−4.15	0.82	−0.46	−0.08	−1.53
160	9.86	−5.73	−1.10	0.06	0.04	0.19	−5.63	0.96	−0.11	−0.02	−2.20
180	12.66	−3.90	−1.23	0.05	0.03	0.24	−6.77	1.15	0.04	0.04	−2.63
$max - min$	13.04	8.55	0.86	0.13	0.09	0.30	7.78	6.48	2.71	0.33	2.72

Table S16: Set of geometries with most populated dihedral angles of hydroxyl groups to examine the impact of hydroxyl group orientations on calculated couplings. The initial structure was **5b** with an (*R*)-(−*gauche*) conformation for aglycone and *gt* for hydroxymethyl group.

	Dihedral angle [deg]			
no.	C3-C2-O2-H	C4-C3-O3-H	C5-C4-O4-H	H6-C6-O6-H
1	−180	−180	−180	−180
2	−180	−180	−180	−60
3	−180	−180	−180	60
4	−180	−180	90	−180
5	−180	−180	90	−60
6	−180	−180	90	−60
7	−180	−60	−180	−180
8	−180	−60	−180	−60
9	−180	−60	−180	60
10	−180	−60	90	−180
11	−180	−60	90	−60
12	−180	−60	90	−60
13	−180	60	−180	−180
14	−180	60	−180	−60
15	−180	60	−180	60
16	−180	60	90	−180
17	−180	60	90	−60
18	−180	60	90	−60
19	−60	−180	−180	−180
20	−60	−180	−180	−60
21	−60	−180	−180	60
22	−60	−180	90	−180
23	−60	−180	90	−60
24	−60	−180	90	−60
25	−60	−60	−180	−180
26	−60	−60	−180	−60
27	−60	−60	−180	60
28	−60	−60	90	−180
29	−60	−60	90	−60
30	−60	−60	90	−60
31	−60	60	−180	−180
32	−60	60	−180	−60
33	−60	60	−180	60
34	−60	60	90	−180
35	−60	60	90	−60
36	−60	60	90	−60
37	60	−180	−180	−180
38	60	−180	−180	−60
39	60	−180	−180	60
40	60	−180	90	−180
41	60	−180	90	−60
42	60	−180	90	−60
43	60	−60	−180	−180
44	60	−60	−180	−60
45	60	−60	−180	60
46	60	−60	90	−180
47	60	−60	90	−60
48	60	−60	90	−60
49	60	60	−180	−180
50	60	60	−180	−60
51	60	60	−180	60
52	60	60	90	−180
53	60	60	90	−60
54	60	60	90	−60

Table S17: Calculated shielding constants for the set of geometries listed in the Table S16.

no.	C1	C2	C3	C4	C5	C6
1	94.4	102.6	99.9	106.4	101.1	114.3
2	94.3	102.6	99.7	106.0	98.9	114.6
3	94.5	102.5	99.8	106.0	103.1	114.9
4	94.4	102.4	96.9	107.1	98.3	114.5
5	94.3	102.4	96.7	106.7	95.8	114.7
6	94.5	102.3	96.8	106.8	100.1	115.0
7	93.9	100.4	100.2	101.6	100.9	114.1
8	93.8	100.3	100.0	101.3	98.8	114.5
9	94.0	100.3	100.1	101.2	102.9	114.8
10	93.8	100.0	97.2	102.5	98.1	114.4
11	93.7	100.0	97.0	102.2	95.6	114.7
12	93.9	99.9	97.1	102.1	100.0	114.9
13	94.0	105.2	100.6	104.8	101.6	114.2
14	93.9	105.1	100.4	104.4	99.4	114.5
15	94.1	105.1	100.5	104.5	103.5	114.8
16	93.9	104.8	96.9	105.2	98.8	114.5
17	93.8	104.7	96.7	104.8	96.2	114.8
18	94.0	104.7	96.8	104.9	100.6	115.0
19	96.2	103.4	97.4	107.0	101.6	114.3
20	96.2	103.4	97.2	106.6	99.4	114.6
21	96.4	103.3	97.3	106.7	103.4	114.9
22	96.2	103.3	94.3	107.7	98.8	114.5
23	96.2	103.3	94.1	107.3	96.2	114.8
24	96.4	103.2	94.2	107.4	100.5	115.0
25	95.5	101.0	97.4	102.1	101.3	114.2
26	95.6	100.9	97.3	101.7	99.2	114.5
27	95.7	100.8	97.4	101.7	103.2	114.8
28	95.5	100.6	94.4	103.0	98.5	114.4
29	95.5	100.5	94.2	102.7	96.0	114.7
30	95.7	100.4	94.3	102.7	100.3	115.0
31	95.6	105.9	97.9	105.1	101.9	114.2
32	95.7	105.8	97.7	104.7	99.7	114.6
33	95.8	105.8	97.8	104.8	103.8	114.9
34	95.6	105.5	94.2	105.5	99.1	114.5
35	95.6	105.4	93.9	105.1	96.6	114.8
36	95.8	105.4	94.1	105.2	100.9	115.1
37	92.7	103.1	94.2	106.5	101.2	114.3
38	92.6	103.0	94.0	106.1	98.9	114.6
39	92.8	103.0	94.1	106.2	103.1	115.0
40	92.6	102.9	91.1	107.2	98.3	114.5
41	92.5	102.9	90.9	106.8	95.7	114.8
42	92.7	102.8	91.1	106.9	100.1	115.1
43	92.0	101.0	94.7	101.7	101.0	114.2
44	91.9	100.9	94.6	101.4	98.8	114.5
45	92.1	100.8	94.7	101.3	103.0	114.8
46	91.9	100.5	91.6	102.6	98.1	114.4
47	91.8	100.5	91.4	102.3	95.6	114.7
48	92.0	100.4	91.6	102.3	100.0	115.0
49	92.1	105.6	95.0	104.9	101.6	114.3
50	92.0	105.6	94.9	104.6	99.4	114.6
51	92.2	105.6	95.0	104.6	103.6	114.9
52	92.0	105.2	91.3	105.3	98.8	114.6
53	91.8	105.2	91.1	104.9	96.2	114.9
54	92.1	105.1	91.3	105.0	100.6	115.1
$max - min$	4.6	6.0	9.6	6.5	8.2	1.0

Table S18: Calculated coupling constants for the set of geometries listed in the Table S16.

no.	$^{1}J(^{15}\mathrm{N,C1})$	$^{2}J(^{15}\mathrm{N,C2})$	$^{3}J(^{15}\mathrm{N,C3})$	$^{3}J(^{15}\mathrm{N,C5})$	$^{2}J(^{15}\mathrm{N,H1})$	$^{3}J(^{15}\mathrm{N,H2})$	
1	−9.13	−2.00	−2.60	−0.70	−1.71	−2.55	13.22
2	−9.42	−2.08	−2.61	−0.76	−1.70	−2.52	13.16
3	−9.44	−2.07	−2.63	−0.82	−1.73	−2.53	13.21
4	−9.18	−2.04	−2.62	−0.64	−1.68	−2.51	13.18
5	−9.47	−2.12	−2.62	−0.69	−1.67	−2.47	13.12
6	−9.49	−2.11	−2.64	−0.75	−1.69	−2.48	13.17
7	−9.11	−1.91	−2.68	−0.73	−1.65	−2.47	13.14
8	−9.39	−1.99	−2.68	−0.78	−1.64	−2.44	13.08
9	−9.41	−1.98	−2.71	−0.85	−1.67	−2.45	13.12
10	−9.14	−1.93	−2.69	−0.67	−1.62	−2.43	13.09
11	−9.42	−2.01	−2.70	−0.72	−1.62	−2.40	13.04
12	−9.45	−2.00	−2.72	−0.78	−1.64	−2.41	13.08
13	−9.44	−2.32	−2.98	−0.71	−1.67	−2.60	13.18
14	−9.72	−2.40	−2.98	−0.77	−1.66	−2.57	13.12
15	−9.74	−2.39	−3.00	−0.83	−1.68	−2.58	13.16
16	−9.48	−2.35	−2.99	−0.66	−1.64	−2.56	13.14
17	−9.77	−2.44	−2.98	−0.71	−1.64	−2.53	13.08
18	−9.79	−2.43	−3.01	−0.77	−1.66	−2.54	13.12
19	−8.87	−1.81	−3.09	−0.59	−1.34	−2.41	12.77
20	−9.17	−1.90	−3.09	−0.64	−1.34	−2.38	12.72
21	−9.19	−1.89	−3.11	−0.70	−1.36	−2.39	12.76
22	−8.91	−1.85	−3.10	−0.54	−1.31	−2.36	12.73
23	−9.21	−1.94	−3.10	−0.59	−1.30	−2.33	12.67
24	−9.24	−1.93	−3.13	−0.64	−1.32	−2.34	12.72
25	−8.78	−1.72	−3.15	−0.62	−1.29	−2.33	12.69
26	−9.07	−1.80	−3.16	−0.66	−1.29	−2.30	12.64
27	−9.10	−1.80	−3.18	−0.73	−1.31	−2.31	12.68
28	−8.81	−1.74	−3.17	−0.57	−1.26	−2.28	12.65
29	−9.11	−1.83	−3.17	−0.61	−1.26	−2.26	12.59
30	−9.13	−1.82	−3.20	−0.68	−1.28	−2.27	12.63
31	−9.04	−2.11	−3.46	−0.61	−1.31	−2.44	12.73
32	−9.34	−2.20	−3.46	−0.66	−1.31	−2.41	12.67
33	−9.36	−2.19	−3.48	−0.71	−1.33	−2.42	12.72
34	−9.09	−2.14	−3.46	−0.56	−1.29	−2.40	12.69
35	−9.38	−2.23	−3.46	−0.61	−1.29	−2.37	12.63
36	−9.41	−2.22	−3.48	−0.67	−1.31	−2.38	12.68
37	−9.06	−1.90	−2.91	−0.59	−1.48	−2.39	12.52
38	−9.35	−1.98	−2.92	−0.64	−1.47	−2.37	12.45
39	−9.37	−1.97	−2.94	−0.70	−1.49	−2.37	12.49
40	−9.10	−1.94	−2.92	−0.53	−1.45	−2.35	12.48
41	−9.39	−2.02	−2.93	−0.58	−1.44	−2.32	12.40
42	−9.41	−2.01	−2.95	−0.64	−1.46	−2.33	12.45
43	−9.03	−1.81	−2.98	−0.61	−1.42	−2.33	12.45
44	−9.32	−1.88	−2.98	−0.66	−1.42	−2.31	12.38
45	−9.35	−1.87	−3.01	−0.72	−1.44	−2.32	12.42
46	−9.06	−1.83	−2.98	−0.56	−1.40	−2.29	12.40
47	−9.35	−1.91	−2.99	−0.61	−1.39	−2.27	12.33
48	−9.37	−1.90	−3.02	−0.67	−1.41	−2.27	12.37
49	−9.36	−2.20	−3.27	−0.60	−1.44	−2.45	12.49
50	−9.64	−2.28	−3.28	−0.65	−1.44	−2.42	12.42
51	−9.66	−2.27	−3.30	−0.71	−1.46	−2.43	12.46
52	−9.40	−2.23	−3.27	−0.55	−1.42	−2.41	12.45
53	−9.68	−2.31	−3.28	−0.60	−1.41	−2.38	12.38
54	−9.70	−2.30	−3.30	−0.66	−1.43	−2.39	12.42
$max - min$	1.01	0.72	0.88	0.31	0.47	0.34	0.89

Table S19: Calculated coupling constants for the set of geometries listed in the Table S16.

no.	^3J(H1,H2)	^3J(H2,H3)	^3J(H3,H4)	^3J(H4,H5)	^3J(NH,H1)
1	8.32	8.59	8.68	9.49	13.22
2	8.26	8.58	8.67	9.14	13.16
3	8.27	8.58	8.66	9.53	13.21
4	8.29	8.21	8.19	9.38	13.18
5	8.23	8.19	8.16	9.01	13.12
6	8.25	8.20	8.17	9.41	13.17
7	8.10	8.17	7.80	9.03	13.14
8	8.04	8.16	7.81	8.71	13.08
9	8.05	8.15	7.79	9.07	13.12
10	8.07	7.86	7.57	9.00	13.09
11	8.02	7.84	7.55	8.65	13.04
12	8.03	7.84	7.54	9.03	13.08
13	8.41	9.10	8.41	9.43	13.18
14	8.36	9.08	8.39	9.09	13.12
15	8.37	9.08	8.38	9.47	13.16
16	8.38	8.65	7.72	9.29	13.14
17	8.32	8.62	7.68	8.92	13.08
18	8.34	8.62	7.68	9.32	13.12
19	8.62	8.22	8.59	9.52	12.77
20	8.56	8.20	8.58	9.18	12.72
21	8.57	8.19	8.58	9.57	12.73
22	8.59	7.84	8.12	9.41	12.73
23	8.53	7.81	8.09	9.04	12.67
24	8.54	7.81	8.09	9.45	12.72
25	8.37	7.68	7.67	9.03	12.69
26	8.30	7.66	7.67	8.72	12.64
27	8.31	7.64	7.66	9.07	12.68
28	8.35	7.37	7.45	9.00	12.65
29	8.28	7.34	7.43	8.66	12.59
30	8.29	7.33	7.43	9.04	12.63
31	8.71	8.63	8.31	9.44	12.73
32	8.65	8.61	8.30	9.11	12.67
33	8.66	8.60	8.29	9.49	12.72
34	8.67	8.18	7.63	9.31	12.69
35	8.60	8.14	7.60	8.94	12.63
36	8.62	8.14	7.60	9.34	12.68
37	7.99	7.67	8.32	9.45	12.52
38	7.91	7.65	8.31	9.10	12.45
39	7.93	7.66	8.31	9.49	12.49
40	7.95	7.31	7.84	9.32	12.48
41	7.88	7.28	7.81	8.95	12.40
42	7.89	7.29	7.82	9.36	12.45
43	7.86	7.52	7.55	8.99	12.45
44	7.78	7.50	7.55	8.67	12.38
45	7.80	7.49	7.54	9.03	12.42
46	7.82	7.21	7.30	8.94	12.40
47	7.75	7.18	7.29	8.60	12.33
48	7.76	7.18	7.28	8.97	12.37
49	8.12	8.22	8.08	9.38	12.49
50	8.04	8.20	8.07	9.04	12.42
51	8.06	8.20	8.06	9.43	12.46
52	8.08	7.79	7.40	9.23	12.45
53	8.00	7.76	7.37	8.87	12.38
54	8.01	7.77	7.37	9.27	12.42
$max - min$	0.97	1.91	1.40	0.97	

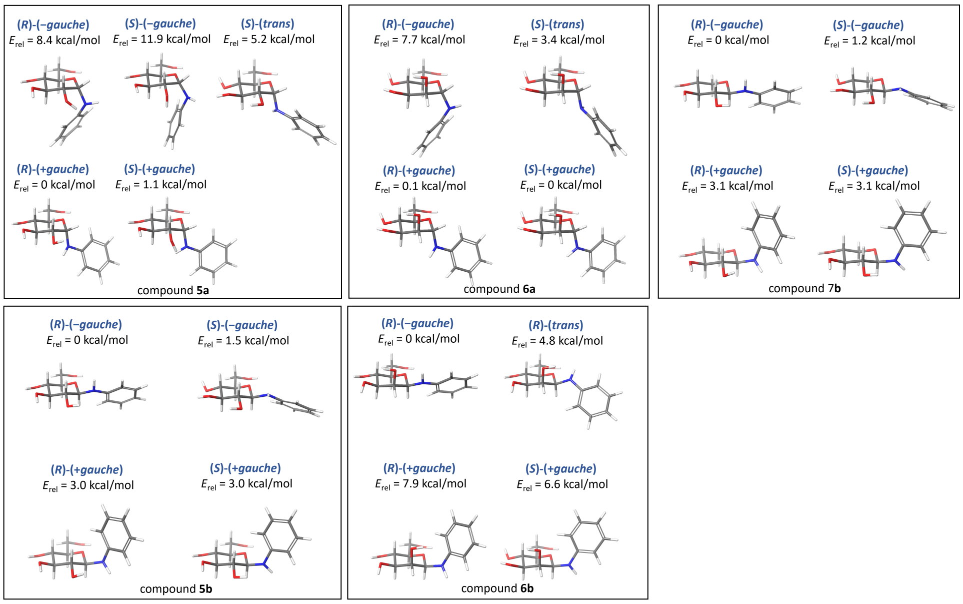

Figure S33: DFT optimized geometries with their relative energies.

Table S20: Cartesian coordinates of optimized geometry **5a** (R)-($-$*gauche*).

Compound: 5a (R)-($-$*gauche*)
Number of Imaginary Frequencies: 0
Sum of Electronic and Thermal Free Energies = −898.091117 Hartree

Center Number	Atomic Number	Atomic Type	Coordinates (Angstroms) X	Y	Z
1	8	0	−0.890196	−0.976773	−1.286556
2	6	0	−0.993898	−1.276187	0.120046
3	6	0	−2.017833	−0.352602	0.779350
4	6	0	−1.573470	1.089565	0.575344
5	6	0	−1.432514	1.397812	−0.917146
6	6	0	−0.580463	0.357210	−1.689892
7	1	0	−0.020007	−1.143159	0.606406
8	1	0	−2.997756	−0.496193	0.294773
9	1	0	−0.610549	1.226243	1.083876
10	1	0	−2.445385	1.354889	−1.339277
11	7	0	0.839823	0.718564	−1.746636
12	6	0	−1.374971	−2.751078	0.175626
13	8	0	−2.094417	−0.672771	2.163367
14	8	0	−2.561237	1.923803	1.180981
15	8	0	−0.931561	2.713744	−1.123901
16	1	0	−0.897633	0.392584	−2.734679
17	1	0	−1.429865	−3.076569	1.217339
18	8	0	−0.398630	−3.558115	−0.470168
19	1	0	−2.368378	−2.883395	−0.281982
20	1	0	−2.558689	0.073985	2.579829
21	1	0	−2.236875	2.837381	1.115938
22	1	0	−0.033648	2.586907	−1.493161
23	1	0	−0.269524	−3.155362	−1.345820
24	6	0	1.746136	0.475584	−0.656402
25	6	0	1.898103	1.424580	0.364046
26	6	0	2.550505	−0.672940	−0.645209
27	6	0	2.811444	1.208081	1.397127
28	6	0	3.604721	0.058292	1.409068
29	6	0	3.476969	−0.876559	0.378932
30	1	0	1.308006	2.335150	0.337670
31	1	0	2.441919	−1.408560	−1.438102
32	1	0	2.915460	1.951120	2.183011
33	1	0	4.322156	−0.103138	2.208489
34	1	0	4.094082	−1.770855	0.373558
35	1	0	1.227879	0.319365	−2.596538

Table S21: Cartesian coordinates of optimized geometry **5a** (S)-($-$*gauche*).

Compound: 5a (S)-($-$*gauche*)
Number of Imaginary Frequencies: 0
Sum of Electronic and Thermal Free Energies = −898.085508 Hartree

Center Number	Atomic Number	Atomic Type	Coordinates (Angstroms) X	Y	Z
1	8	0	−1.271601	−0.594878	−1.304866
2	6	0	−1.618944	−0.841774	0.072601
3	6	0	−2.116873	0.430239	0.752245
4	6	0	−1.012799	1.474153	0.656135
5	6	0	−0.686792	1.747795	−0.810607
6	6	0	−0.393674	0.467301	−1.657825
7	1	0	−0.745940	−1.201621	0.627598
8	1	0	−3.010774	0.809164	0.230688
9	1	0	−0.122178	1.084998	1.176962
10	1	0	−1.573882	2.218681	−1.247897
11	7	0	1.010769	0.062832	−1.778920
12	6	0	−2.643409	−1.967306	0.007766
13	8	0	−2.421255	0.118822	2.106037
14	8	0	−1.477642	2.654537	1.303281
15	8	0	0.342223	2.726013	−0.936610
16	1	0	−0.669057	0.711648	−2.687677
17	1	0	−2.932252	−2.259710	1.020037
18	8	0	−2.088242	−3.116055	−0.622999
19	1	0	−3.541256	−1.614437	−0.523385
20	1	0	−2.527399	0.971811	2.560854
21	1	0	−0.822302	3.349295	1.119614
22	1	0	1.134753	2.380866	−0.490182
23	1	0	−1.739094	−2.799568	−1.473756
24	6	0	1.855773	−0.270897	−0.686451
25	6	0	1.680227	−1.478855	0.010843
26	6	0	2.951623	0.544542	−0.351921
27	6	0	2.540655	−1.823598	1.053036
28	6	0	3.614841	−0.996704	1.394949
29	6	0	3.824834	0.180712	0.675419
30	1	0	0.890425	−2.157790	−0.292392
31	1	0	3.134639	1.457650	−0.915974
32	1	0	2.384723	−2.759089	1.583647
33	1	0	4.288197	−1.276151	2.199930
34	1	0	4.666168	0.825652	0.914133
35	1	0	1.469219	0.717636	−2.405631

Table S22: Cartesian coordinates of optimized geometry **5a** (*S*)-(*trans*).

Compound: 5a (*S*)-(*trans*)
Number of Imaginary Frequencies: 0
Sum of Electronic and Thermal Free Energies = −898.096094 Hartree

Center Number	Atomic Number	Atomic Type	Coordinates (Angstroms) X	Y	Z
1	8	0	−0.975394	−0.912454	−0.947004
2	6	0	−2.025749	−1.024381	0.035483
3	6	0	−2.810615	0.291038	0.143738
4	6	0	−1.839544	1.436912	0.410185
5	6	0	−0.766968	1.475005	−0.670868
6	6	0	−0.021610	0.128622	−0.751784
7	1	0	−1.603193	−1.261206	1.023547
8	1	0	−3.335104	0.488330	−0.801160
9	1	0	−1.358233	1.272351	1.386652
10	1	0	−1.267398	1.622643	−1.641274
11	7	0	0.837346	−0.143590	0.398492
12	6	0	−2.860631	−2.215130	−0.421254
13	8	0	−3.732269	0.261408	1.236169
14	8	0	−2.496578	2.700109	0.394794
15	8	0	0.170258	2.513550	−0.440198
16	1	0	0.593508	0.126871	−1.657088
17	1	0	−3.665576	−2.394591	0.297013
18	8	0	−2.082033	−3.401219	−0.479433
19	1	0	−3.311799	−1.983328	−1.399050
20	1	0	−4.591145	−0.046312	0.908859
21	1	0	−3.198833	2.649601	1.065234
22	1	0	−0.355417	3.306628	−0.238308
23	1	0	−1.306491	−3.178378	−1.022159
24	6	0	2.232791	−0.175850	0.273215
25	6	0	2.921236	0.712305	−0.574260
26	6	0	2.980621	−1.086408	1.044943
27	6	0	4.313698	0.662648	−0.660604
28	6	0	5.050985	−0.245376	0.101952
29	6	0	4.370649	−1.113424	0.961852
30	1	0	2.368841	1.461538	−1.130727
31	1	0	2.462355	−1.777513	1.706524
32	1	0	4.824910	1.356095	−1.323922
33	1	0	6.134625	−0.272801	0.033919
34	1	0	4.924162	−1.827314	1.566742
35	1	0	0.482310	−0.904410	0.965549

Table S23: Cartesian coordinates of optimized geometry **5a** (*R*)-(+*gauche*).

Compound: 5a (*R*)-(+*gauche*)
Number of Imaginary Frequencies: 0
Sum of Electronic and Thermal Free Energies = −898.104446 Hartree

Center Number	Atomic Number	Atomic Type	Coordinates (Angstroms) X	Y	Z
1	8	0	0.589172	0.829377	−0.934558
2	6	0	1.400597	1.236808	0.180086
3	6	0	2.685446	0.406592	0.197507
4	6	0	2.328985	−1.074589	0.260843
5	6	0	1.377083	−1.465296	−0.869091
6	6	0	0.142773	−0.532567	−0.906749
7	1	0	0.849522	1.076600	1.115823
8	1	0	3.243187	0.591148	−0.735081
9	1	0	1.834612	−1.266785	1.225347
10	1	0	1.906205	−1.359494	−1.824053
11	7	0	−0.726201	−0.825904	0.205442
12	6	0	1.646415	2.728466	−0.010570
13	8	0	3.461800	0.788856	1.326824
14	8	0	3.549636	−1.805332	0.185891
15	8	0	1.025282	−2.828404	−0.632905
16	1	0	−0.383105	−0.639491	−1.865161
17	1	0	2.255204	3.104734	0.815252
18	8	0	0.429762	3.463802	−0.003883
19	1	0	2.202758	2.885727	−0.948336
20	1	0	4.173633	0.130006	1.397528
21	1	0	3.312328	−2.746946	0.226816
22	1	0	0.660849	−3.202277	−1.451676
23	1	0	−0.137854	3.040801	−0.669491
24	6	0	−2.085252	−0.483040	0.177996
25	6	0	−2.988859	−1.187444	0.996485
26	6	0	−2.574916	0.566721	−0.619913
27	6	0	−4.340636	−0.854251	1.009961
28	6	0	−4.827600	0.183757	0.208488
29	6	0	−3.934287	0.885898	−0.602157
30	1	0	−2.620998	−1.999425	1.620103
31	1	0	−1.892760	1.138602	−1.238960
32	1	0	−5.018927	−1.414648	1.648430
33	1	0	−5.882712	0.441032	0.219922
34	1	0	−4.292261	1.699275	−1.228435
35	1	0	−0.549987	−1.750241	0.585607

Table S24: Cartesian coordinates of optimized geometry **5a** (*S*)-(+*gauche*).

Compound: 5a (*S*)-(+*gauche*)<br>Number of Imaginary Frequencies: 0<br>Sum of Electronic and Thermal Free Energies = −898.102649 Hartree					
Center Number	Atomic Number	Atomic Type	Coordinates (Angstroms) X	Y	Z
1	8	0	0.556772	0.788093	−0.962932
2	6	0	1.346650	1.260787	0.140792
3	6	0	2.664990	0.483385	0.209474
4	6	0	2.366112	−1.010306	0.310532
5	6	0	1.463134	−1.458423	−0.844512
6	6	0	0.184306	−0.599942	−0.910620
7	1	0	0.795536	1.124766	1.082930
8	1	0	3.232061	0.659937	−0.718796
9	1	0	1.861817	−1.193253	1.274720
10	1	0	2.014134	−1.304506	−1.777973
11	7	0	−0.723746	−0.964813	0.142826
12	6	0	1.525649	2.754911	−0.102327
13	8	0	3.397749	0.941607	1.339176
14	8	0	3.607679	−1.704163	0.288156
15	8	0	1.183822	−2.850713	−0.770921
16	1	0	−0.316176	−0.778416	−1.863636
17	1	0	2.094857	3.195555	0.719821
18	8	0	0.270143	3.418658	−0.153379
19	1	0	2.094162	2.901062	−1.034518
20	1	0	4.146793	0.330346	1.442487
21	1	0	3.389728	−2.652202	0.275988
22	1	0	0.399768	−2.946536	−0.199494
23	1	0	−0.268311	2.908960	−0.782685
24	6	0	−2.073171	−0.552629	0.158034
25	6	0	−2.683807	0.103966	−0.922726
26	6	0	−2.846737	−0.848147	1.295049
27	6	0	−4.040739	0.431597	−0.866455
28	6	0	−4.806848	0.132745	0.260545
29	6	0	−4.195180	−0.505226	1.344269
30	1	0	−2.108142	0.381523	−1.798518
31	1	0	−2.382448	−1.356523	2.137119
32	1	0	−4.494064	0.939003	−1.714050
33	1	0	−5.859447	0.397232	0.298416
34	1	0	−4.771346	−0.743320	2.234539
35	1	0	−0.318180	−1.014102	1.070747

Table S25: Cartesian coordinates of optimized geometry **5b** (R)-($-$*gauche*).

Compound: 5b (R)-($-$*gauche*)
Number of Imaginary Frequencies: 0
Sum of Electronic and Thermal Free Energies = −898.106420 Hartree

Center Number	Atomic Number	Atomic Type	Coordinates (Angstroms) X	Y	Z
1	8	0	0.454578	0.669200	−0.471254
2	6	0	1.632328	1.262011	0.094069
3	6	0	2.840542	0.370341	−0.205995
4	6	0	2.586472	−1.031360	0.344167
5	6	0	1.268858	−1.601913	−0.173129
6	6	0	0.123157	−0.611453	0.097485
7	1	0	1.512631	1.354808	1.185312
8	1	0	2.965518	0.299391	−1.298546
9	1	0	2.523595	−0.964574	1.441999
10	1	0	1.351077	−1.750263	−1.262236
11	7	0	−1.090741	−1.108880	−0.461298
12	6	0	1.723789	2.656942	−0.512432
13	8	0	3.994375	0.945354	0.393350
14	8	0	3.702089	−1.833974	−0.031643
15	8	0	1.060928	−2.838233	0.495197
16	1	0	−0.000374	−0.503563	1.183295
17	1	0	2.579696	3.188706	−0.089521
18	8	0	0.560392	3.417397	−0.214727
19	1	0	1.875519	2.567227	−1.599883
20	1	0	4.688225	0.265562	0.340733
21	1	0	3.571734	−2.708366	0.371075
22	1	0	0.188278	−3.167087	0.219100
23	1	0	−0.190232	2.849744	−0.460547
24	6	0	−2.349582	−0.547313	−0.165097
25	6	0	−2.547904	0.381672	0.869600
26	6	0	−3.461953	−0.971468	−0.914476
27	6	0	−3.831859	0.853591	1.151439
28	6	0	−4.933899	0.428330	0.408486
29	6	0	−4.735749	−0.485324	−0.631277
30	1	0	−1.707864	0.755038	1.444761
31	1	0	−3.319776	−1.691750	−1.717049
32	1	0	−3.962507	1.572481	1.956191
33	1	0	−5.927984	0.805249	0.630158
34	1	0	−5.578661	−0.827220	−1.226249
35	1	0	−0.997795	−1.385513	−1.433933

Table S26: Cartesian coordinates of optimized geometry **5b** (*S*)-(−*gauche*).

Compound: 5b (*S*)-(−*gauche*)
Number of Imaginary Frequencies: 0
Sum of Electronic and Thermal Free Energies = −898.104024 Hartree

Center Number	Atomic Number	Atomic Type	Coordinates (Angstroms) X	Y	Z
1	8	0	0.540853	0.698755	−0.577767
2	6	0	1.686626	1.225548	0.107335
3	6	0	2.870869	0.263873	−0.082250
4	6	0	2.473133	−1.132175	0.396565
5	6	0	1.176143	−1.598434	−0.257892
6	6	0	0.083254	−0.549179	−0.028002
7	1	0	1.466438	1.317685	1.182868
8	1	0	3.126602	0.209410	−1.149194
9	1	0	2.314238	−1.086794	1.486425
10	1	0	1.338610	−1.684358	−1.343837
11	7	0	−1.129254	−0.930154	−0.683916
12	6	0	1.906510	2.618530	−0.470726
13	8	0	4.010706	0.646706	0.691528
14	8	0	3.469807	−2.100313	0.090580
15	8	0	0.736424	−2.837434	0.281612
16	1	0	−0.074462	−0.406245	1.054955
17	1	0	2.729672	3.111043	0.054481
18	8	0	0.754940	3.433451	−0.297212
19	1	0	2.176942	2.532795	−1.535028
20	1	0	4.571531	1.223684	0.151165
21	1	0	4.293970	−1.775521	0.491700
22	1	0	1.497555	−3.440656	0.229438
23	1	0	0.021391	2.935279	−0.695827
24	6	0	−2.373394	−0.449604	−0.257138
25	6	0	−2.501650	0.777667	0.418973
26	6	0	−3.536006	−1.191458	−0.538626
27	6	0	−3.761398	1.230987	0.814491
28	6	0	−4.912319	0.490707	0.538032
29	6	0	−4.787257	−0.722439	−0.146568
30	1	0	−1.623277	1.383573	0.613694
31	1	0	−3.449789	−2.140587	−1.063031
32	1	0	−3.838425	2.180533	1.338247
33	1	0	−5.889082	0.852763	0.845246
34	1	0	−5.670198	−1.314888	−0.372705
35	1	0	−1.135845	−1.900099	−0.976260

Table S27: Cartesian coordinates of optimized geometry **5b** (*R*)-(+*gauche*).

Compound: 5b (*R*)-(+*gauche*)					
Number of Imaginary Frequencies: 0					
Sum of Electronic and Thermal Free Energies = −898.101571 Hartree					
Center Number	Atomic Number	Atomic Type	Coordinates (Angstroms) X	Y	Z
1	8	0	−0.770345	1.018982	−0.450747
2	6	0	−2.184411	0.879499	−0.253474
3	6	0	−2.469804	−0.326130	0.646884
4	6	0	−1.847961	−1.575396	0.030500
5	6	0	−0.363483	−1.368665	−0.249977
6	6	0	−0.162533	−0.102707	−1.112772
7	1	0	−2.674568	0.725196	−1.227963
8	1	0	−2.005605	−0.153893	1.631414
9	1	0	−2.356479	−1.776216	−0.926865
10	1	0	0.164827	−1.254186	0.704166
11	7	0	1.203685	0.217073	−1.420326
12	6	0	−2.650211	2.207501	0.331407
13	8	0	−3.878690	−0.469495	0.777239
14	8	0	−2.074121	−2.652580	0.934469
15	8	0	0.085753	−2.533749	−0.938395
16	1	0	−0.663462	−0.268645	−2.076647
17	1	0	−3.735059	2.191852	0.461404
18	8	0	−2.341332	3.286804	−0.539774
19	1	0	−2.185939	2.346376	1.320857
20	1	0	−4.022234	−1.334429	1.198189
21	1	0	−1.657205	−3.436197	0.538181
22	1	0	1.054882	−2.554435	−0.892134
23	1	0	−1.399815	3.180717	−0.759814
24	6	0	2.258258	0.252610	−0.497008
25	6	0	2.071168	0.655285	0.839229
26	6	0	3.559451	−0.069565	−0.927777
27	6	0	3.156513	0.705464	1.714714
28	6	0	4.445560	0.383946	1.283720
29	6	0	4.637159	0.003809	−0.047654
30	1	0	1.083240	0.948958	1.174827
31	1	0	3.719694	−0.379345	−1.958237
32	1	0	2.988560	1.015039	2.743207
33	1	0	5.285946	0.433484	1.969850
34	1	0	5.632036	−0.248241	−0.405847
35	1	0	1.477040	−0.029154	−2.361385

Table S28: Cartesian coordinates of optimized geometry **5b** (*S*)-(+*gauche*).

Compound: 5b (*S*)-(+*gauche*)<br>Number of Imaginary Frequencies: 0<br>Sum of Electronic and Thermal Free Energies = −898.101680 Hartree					
Center Number	Atomic Number	Atomic Type	Coordinates (Angstroms) X	Y	Z
1	8	0	−0.593093	0.938542	−0.474209
2	6	0	−2.014289	1.037369	−0.304718
3	6	0	−2.504071	−0.072897	0.630313
4	6	0	−2.089373	−1.432033	0.072388
5	6	0	−0.587352	−1.486930	−0.184787
6	6	0	−0.169612	−0.298121	−1.076635
7	1	0	−2.507562	0.928196	−1.283812
8	1	0	−2.031271	0.059131	1.617058
9	1	0	−2.608521	−1.583506	−0.887643
10	1	0	−0.059814	−1.437733	0.776929
11	7	0	1.231529	−0.293680	−1.365888
12	6	0	−2.270042	2.443092	0.225730
13	8	0	−3.918888	0.016930	0.739476
14	8	0	−2.514304	−2.413754	1.014480
15	8	0	−0.319533	−2.719817	−0.841104
16	1	0	−0.679764	−0.417811	−2.042531
17	1	0	−3.345382	2.606878	0.330024
18	8	0	−1.774939	3.427146	−0.672043
19	1	0	−1.807878	2.541028	1.220985
20	1	0	−4.204080	−0.806547	1.171993
21	1	0	−2.311263	−3.282919	0.631181
22	1	0	0.623681	−2.708811	−1.078905
23	1	0	−0.858171	3.165142	−0.864008
24	6	0	2.275260	−0.035057	−0.456315
25	6	0	2.075267	0.297371	0.894736
26	6	0	3.595813	−0.135281	−0.935516
27	6	0	3.171411	0.494055	1.738240
28	6	0	4.477882	0.389853	1.260067
29	6	0	4.679395	0.081825	−0.089126
30	1	0	1.072078	0.438902	1.275808
31	1	0	3.763580	−0.393402	−1.978687
32	1	0	2.991953	0.749127	2.779649
33	1	0	5.323657	0.552071	1.921779
34	1	0	5.687860	0.001073	−0.486626
35	1	0	1.454513	−0.046898	−2.321051

Table S29: Cartesian coordinates of optimized geometry **6a** (*R*)-(−*gauche*).

Compound: 6a (*R*)-(−*gauche*)
Number of Imaginary Frequencies: 0
Sum of Electronic and Thermal Free Energies = −898.090261 Hartree

Center Number	Atomic Number	Atomic Type	Coordinates (Angstroms) X	Y	Z
1	8	0	1.110478	0.997575	−1.116621
2	6	0	1.080117	1.146915	0.312440
3	6	0	1.854158	−0.013841	0.943768
4	6	0	1.177120	−1.324625	0.556894
5	6	0	1.078149	−1.465264	−0.968329
6	6	0	0.538067	−0.202524	−1.671405
7	1	0	0.043737	1.141294	0.676194
8	1	0	2.880049	−0.015456	0.554306
9	1	0	0.173870	−1.335383	1.002915
10	1	0	0.430070	−2.316072	−1.218867
11	7	0	−0.903846	−0.146329	−1.863475
12	6	0	1.682735	2.522166	0.577290
13	8	0	1.854347	0.027004	2.374123
14	8	0	1.931583	−2.442924	1.023925
15	8	0	2.385032	−1.683046	−1.497150
16	1	0	0.956380	−0.223913	−2.680420
17	1	0	1.647514	2.744152	1.647750
18	8	0	0.942473	3.537878	−0.086348
19	1	0	2.736925	2.523016	0.256105
20	1	0	2.615520	0.550738	2.667537
21	1	0	2.226178	−2.203277	1.920548
22	1	0	2.821614	−2.264988	−0.847647
23	1	0	0.851959	3.227098	−1.003855
24	6	0	−1.867181	−0.176529	−0.812629
25	6	0	−2.174160	−1.373719	−0.144866
26	6	0	−2.610360	0.974857	−0.502203
27	6	0	−3.163764	−1.401948	0.838958
28	6	0	−3.891587	−0.250436	1.148926
29	6	0	−3.616794	0.935210	0.463438
30	1	0	−1.657127	−2.288577	−0.416168
31	1	0	−2.388128	1.905924	−1.018449
32	1	0	−3.381524	−2.337422	1.347418
33	1	0	−4.669517	−0.279834	1.906372
34	1	0	−4.179380	1.838006	0.686088
35	1	0	−1.091540	0.645527	−2.472638

Table S30: Cartesian coordinates of optimized geometry **6a** (*S*)-(*trans*).

Compound: 6a (*S*)-(*trans*)
Number of Imaginary Frequencies: 0
Sum of Electronic and Thermal Free Energies = −898.097088 Hartree

Center Number	Atomic Number	Atomic Type	Coordinates (Angstroms) X	Y	Z
1	8	0	1.080673	0.897126	−0.943809
2	6	0	2.056113	0.947653	0.118312
3	6	0	2.640675	−0.451325	0.334827
4	6	0	1.509044	−1.413130	0.682497
5	6	0	0.442824	−1.416444	−0.416353
6	6	0	−0.012458	0.012758	−0.766808
7	1	0	1.585696	1.292544	1.050217
8	1	0	3.119844	−0.791259	−0.591612
9	1	0	1.054399	−1.088777	1.629808
10	1	0	−0.427013	−1.983950	−0.055440
11	7	0	−0.911220	0.549170	0.257057
12	6	0	3.070738	1.991923	−0.331362
13	8	0	3.560617	−0.494864	1.429135
14	8	0	1.995259	−2.748507	0.794195
15	8	0	0.961918	−2.005389	−1.602314
16	1	0	−0.503132	−0.019008	−1.749045
17	1	0	3.833508	2.118580	0.441950
18	8	0	2.457625	3.259123	−0.522028
19	1	0	3.563685	1.642813	−1.252467
20	1	0	4.451487	−0.313968	1.092384
21	1	0	2.836654	−2.682127	1.279731
22	1	0	1.484941	−2.769231	−1.297844
23	1	0	1.712095	3.101489	−1.126011
24	6	0	−2.298092	0.350531	0.187547
25	6	0	−3.138882	1.191513	0.945103
26	6	0	−2.889082	−0.670219	−0.579760
27	6	0	−4.518530	1.015513	0.934250
28	6	0	−5.103013	−0.002051	0.171302
29	6	0	−4.276934	−0.836438	−0.580699
30	1	0	−2.695099	1.983960	1.543702
31	1	0	−2.280299	−1.338008	−1.179647
32	1	0	−5.142354	1.680603	1.526057
33	1	0	−6.180332	−0.138823	0.165589
34	1	0	−4.709210	−1.631386	−1.183138
35	1	0	−0.669477	1.510743	0.471803

Table S31: Cartesian coordinates of optimized geometry **6a** (R)-(+*gauche*).

Compound: 6a (R)-(+*gauche*)
Number of Imaginary Frequencies: 0
Sum of Electronic and Thermal Free Energies = −898.102292 Hartree

Center Number	Atomic Number	Atomic Type	Coordinates (Angstroms) X	Y	Z
1	8	0	−0.534596	0.698162	0.846130
2	6	0	−1.329868	1.162792	−0.256593
3	6	0	−2.620411	0.335766	−0.311024
4	6	0	−2.267526	−1.139917	−0.488842
5	6	0	−1.315539	−1.613496	0.616765
6	6	0	−0.107521	−0.667343	0.749039
7	1	0	−0.772403	1.038223	−1.195256
8	1	0	−3.162158	0.454034	0.635679
9	1	0	−1.787065	−1.260908	−1.471973
10	1	0	−0.952951	−2.624793	0.374309
11	7	0	0.818796	−0.875086	−0.334811
12	6	0	−1.551358	2.648908	−0.004810
13	8	0	−3.457728	0.680686	−1.419197
14	8	0	−3.433950	−1.954290	−0.408602
15	8	0	−1.990379	−1.624468	1.865797
16	1	0	0.369684	−0.849273	1.719041
17	1	0	−2.134108	3.077971	−0.825072
18	8	0	−0.319031	3.355014	0.042285
19	1	0	−2.121391	2.778216	0.929322
20	1	0	−4.061735	1.386407	−1.141660
21	1	0	−4.120230	−1.481223	−0.911990
22	1	0	−2.880620	−1.968851	1.668210
23	1	0	0.237326	2.866135	0.672066
24	6	0	2.175619	−0.554102	−0.227987
25	6	0	3.095773	−1.143879	−1.116082
26	6	0	2.652412	0.362772	0.727104
27	6	0	4.450511	−0.831190	−1.043643
28	6	0	4.924965	0.071123	−0.085987
29	6	0	4.014944	0.660408	0.793220
30	1	0	2.739486	−1.852538	−1.860659
31	1	0	1.960049	0.852219	1.403012
32	1	0	5.140836	−1.302359	−1.738754
33	1	0	5.982535	0.311754	−0.030390
34	1	0	4.362149	1.369534	1.540553
35	1	0	0.622490	−1.660818	−0.941024

Table S32: Cartesian coordinates of optimized geometry **6a** (*S*)-(+*gauche*).

Compound: 6a (*S*)-(+*gauche*)<br>Number of Imaginary Frequencies: 0<br>Sum of Electronic and Thermal Free Energies = −898.102489 Hartree					
Center Number	Atomic Number	Atomic Type	Coordinates (Angstroms) X	Y	Z
1	8	0	−0.470977	0.578516	0.917786
2	6	0	−1.161339	1.180554	−0.186727
3	6	0	−2.536565	0.516215	−0.322831
4	6	0	−2.348439	−0.979891	−0.569482
5	6	0	−1.498546	−1.618352	0.538325
6	6	0	−0.197735	−0.828055	0.773454
7	1	0	−0.588283	1.036719	−1.114876
8	1	0	−3.090973	0.648526	0.614468
9	1	0	−1.852977	−1.105143	−1.545775
10	1	0	−1.236928	−2.646714	0.248578
11	7	0	0.773211	−1.144362	−0.232275
12	6	0	−1.205972	2.670557	0.128858
13	8	0	−3.290334	1.011452	−1.432975
14	8	0	−3.601286	−1.657097	−0.572283
15	8	0	−2.224693	−1.617721	1.759374
16	1	0	0.193783	−1.107350	1.755377
17	1	0	−1.691616	3.209536	−0.689815
18	8	0	0.105053	3.202436	0.260887
19	1	0	−1.795714	2.828568	1.046248
20	1	0	−3.812927	1.772102	−1.135928
21	1	0	−4.209912	−1.082852	−1.070414
22	1	0	−3.137499	−1.851712	1.510230
23	1	0	0.571402	2.594511	0.860370
24	6	0	2.099813	−0.708941	−0.192139
25	6	0	2.651174	−0.060756	0.928974
26	6	0	2.931690	−0.959309	−1.301205
27	6	0	3.999177	0.303489	0.935503
28	6	0	4.821280	0.049207	−0.163390
29	6	0	4.271716	−0.582891	−1.283456
30	1	0	2.031821	0.172753	1.787646
31	1	0	2.517838	−1.457886	−2.175070
32	1	0	4.403288	0.801893	1.813120
33	1	0	5.867003	0.341965	−0.151489
34	1	0	4.890824	−0.788181	−2.153125
35	1	0	0.428779	−1.387856	−1.151237

Table S33: Cartesian coordinates of optimized geometry **6b** (*R*)-(−*gauche*).

Compound: 6b (*R*)-(−*gauche*)<br>Number of Imaginary Frequencies: 0<br>Sum of Electronic and Thermal Free Energies = −898.105952 Hartree					
Center Number	Atomic Number	Atomic Type	Coordinates (Angstroms) X	Y	Z
1	8	0	−0.389556	0.536287	0.343805
2	6	0	−1.553204	1.246207	−0.091362
3	6	0	−2.781330	0.364406	0.140070
4	6	0	−2.620604	−0.938023	−0.642909
5	6	0	−1.287783	−1.639877	−0.332668
6	6	0	−0.115575	−0.651943	−0.427625
7	1	0	−1.467053	1.482386	−1.164969
8	1	0	−2.837644	0.121826	1.211784
9	1	0	−2.675467	−0.704074	−1.714216
10	1	0	−1.118888	−2.439867	−1.070920
11	7	0	1.076634	−1.272024	0.039372
12	6	0	−1.561245	2.549757	0.698923
13	8	0	−3.943360	1.066460	−0.281850
14	8	0	−3.717347	−1.779197	−0.258428
15	8	0	−1.315601	−2.178818	0.982417
16	1	0	0.004778	−0.357600	−1.480554
17	1	0	−2.395128	3.177096	0.374802
18	8	0	−0.363541	3.282013	0.474261
19	1	0	−1.695629	2.321477	1.768262
20	1	0	−4.670629	0.421617	−0.246648
21	1	0	−3.880891	−2.419766	−0.969909
22	1	0	−2.208940	−2.554865	1.086336
23	1	0	0.357768	2.643967	0.613013
24	6	0	2.335241	−0.663598	−0.042770
25	6	0	3.408698	−1.235269	0.666872
26	6	0	2.584667	0.467021	−0.841738
27	6	0	4.689885	−0.698302	0.572088
28	6	0	4.937972	0.417934	−0.232908
29	6	0	3.875572	0.990745	−0.934324
30	1	0	3.228482	−2.108645	1.289757
31	1	0	1.774751	0.949915	−1.377736
32	1	0	5.500147	−1.158650	1.131869
33	1	0	5.937918	0.835313	−0.306235
34	1	0	4.044123	1.865302	−1.557746
35	1	0	0.927342	−1.839561	0.865977

Table S34: Cartesian coordinates of optimized geometry **6b** (*R*)-(*trans*).

Compound: 6b (*R*)-(*trans*)
Number of Imaginary Frequencies: 0
Sum of Electronic and Thermal Free Energies = −898.098370 Hartree

Center Number	Atomic Number	Atomic Type	Coordinates (Angstroms) X	Y	Z
1	8	0	−0.868442	1.029022	0.259971
2	6	0	−2.136231	0.965640	−0.409741
3	6	0	−2.865726	−0.321499	−0.018205
4	6	0	−1.984602	−1.521006	−0.361062
5	6	0	−0.612470	−1.404651	0.309158
6	6	0	0.022768	−0.036548	−0.047558
7	1	0	−1.974299	0.974218	−1.499163
8	1	0	−3.046230	−0.317037	1.066577
9	1	0	−1.830826	−1.521775	−1.451119
10	1	0	0.048227	−2.198093	−0.079992
11	7	0	1.194656	0.210122	0.783227
12	6	0	−2.875201	2.236448	−0.008809
13	8	0	−4.091656	−0.381159	−0.736270
14	8	0	−2.717930	−2.681277	0.027447
15	8	0	−0.756967	−1.523042	1.712623
16	1	0	0.286823	−0.012758	−1.117076
17	1	0	−3.844099	2.267731	−0.512876
18	8	0	−2.156917	3.398854	−0.399442
19	1	0	−3.051213	2.220673	1.078359
20	1	0	−4.395183	−1.301728	−0.646073
21	1	0	−2.346662	−3.447916	−0.437414
22	1	0	0.079217	−1.171724	2.076403
23	1	0	−1.258784	3.285176	−0.045044
24	6	0	2.498528	0.115489	0.264753
25	6	0	2.833681	−0.791699	−0.754983
26	6	0	3.514974	0.915667	0.818226
27	6	0	4.149843	−0.877293	−1.214231
28	6	0	5.154839	−0.080358	−0.664721
29	6	0	4.825419	0.814018	0.358760
30	1	0	2.077699	−1.441879	−1.183560
31	1	0	3.268059	1.620088	1.609360
32	1	0	4.386113	−1.583111	−2.006303
33	1	0	6.176963	−0.155315	−1.023834
34	1	0	5.592473	1.445036	0.800129
35	1	0	1.049484	1.043148	1.344875

Table S35: Cartesian coordinates of optimized geometry **6b** (*R*)-(+*gauche*).

Compound: 6b (*R*)-(+*gauche*)
Number of Imaginary Frequencies: 0
Sum of Electronic and Thermal Free Energies = −898.093375 Hartree

Center Number	Atomic Number	Atomic Type	Coordinates (Angstroms) X	Y	Z
1	8	0	0.659521	0.933249	0.444464
2	6	0	2.083402	0.959939	0.277434
3	6	0	2.556995	−0.284297	−0.479526
4	6	0	2.082263	−1.538381	0.254730
5	6	0	0.556206	−1.517974	0.431647
6	6	0	0.185511	−0.202492	1.172360
7	1	0	2.563059	0.977510	1.269604
8	1	0	2.118244	−0.282092	−1.487950
9	1	0	2.542931	−1.537399	1.253904
10	1	0	0.252281	−2.354265	1.082199
11	7	0	−1.210314	−0.007040	1.479837
12	6	0	2.387308	2.268650	−0.441669
13	8	0	3.977546	−0.255432	−0.546817
14	8	0	2.562703	−2.648150	−0.499779
15	8	0	−0.029176	−1.645880	−0.854223
16	1	0	0.692666	−0.239765	2.148796
17	1	0	3.468159	2.381490	−0.556300
18	8	0	1.919986	3.385045	0.303582
19	1	0	1.934666	2.239861	−1.445666
20	1	0	4.237517	−1.157338	−0.805353
21	1	0	2.577504	−3.427356	0.078493
22	1	0	−0.993917	−1.601679	−0.755050
23	1	0	0.994521	3.181895	0.523020
24	6	0	−2.285585	0.044254	0.582592
25	6	0	−3.561322	−0.347740	1.036787
26	6	0	−2.166550	0.543365	−0.730999
27	6	0	−4.675552	−0.247484	0.207320
28	6	0	−4.551410	0.232395	−1.099650
29	6	0	−3.290038	0.620874	−1.555662
30	1	0	−3.671395	−0.733183	2.048136
31	1	0	−1.198654	0.868959	−1.090816
32	1	0	−5.646438	−0.557138	0.585691
33	1	0	−5.420175	0.303427	−1.747330
34	1	0	−3.171732	1.003804	−2.566252
35	1	0	−1.459042	−0.354760	2.396012

Table S36: Cartesian coordinates of optimized geometry **6b** (*S*)-(+*gauche*).

Compound: 6b (*S*)-(+*gauche*)
Number of Imaginary Frequencies: 0
Sum of Electronic and Thermal Free Energies = −898.095464 Hartree

Center Number	Atomic Number	Atomic Type	Coordinates (Angstroms) X	Y	Z
1	8	0	0.439391	0.816639	0.403162
2	6	0	1.828389	1.152565	0.289313
3	6	0	2.569288	0.017010	−0.429425
4	6	0	2.382053	−1.283608	0.350032
5	6	0	0.891010	−1.600082	0.533509
6	6	0	0.192670	−0.376377	1.161161
7	1	0	2.257379	1.286038	1.296174
8	1	0	2.141272	−0.111614	−1.431550
9	1	0	2.857952	−1.167544	1.336334
10	1	0	0.786303	−2.429248	1.250431
11	7	0	−1.215693	−0.531764	1.420473
12	6	0	1.866414	2.488183	−0.444012
13	8	0	3.980156	0.238917	−0.505912
14	8	0	2.961688	−2.380545	−0.350100
15	8	0	0.326016	−1.960869	−0.716426
16	1	0	0.659546	−0.262703	2.151062
17	1	0	2.898795	2.842341	−0.514739
18	8	0	1.130961	3.479362	0.259032
19	1	0	1.476355	2.350375	−1.465257
20	1	0	4.171726	0.736773	−1.315499
21	1	0	3.805781	−2.050308	−0.706281
22	1	0	1.012527	−2.499461	−1.152829
23	1	0	0.266817	3.074149	0.447183
24	6	0	−2.272854	−0.280096	0.527543
25	6	0	−3.558946	−0.116815	1.080787
26	6	0	−2.133135	−0.205929	−0.869593
27	6	0	−4.667132	0.103617	0.266594
28	6	0	−4.525821	0.172308	−1.122072
29	6	0	−3.252427	0.015642	−1.673622
30	1	0	−3.684219	−0.167911	2.160736
31	1	0	−1.156998	−0.333247	−1.315645
32	1	0	−5.645780	0.224611	0.724486
33	1	0	−5.388615	0.345918	−1.758745
34	1	0	−3.118825	0.069140	−2.751656
35	1	0	−1.452920	−0.251485	2.363148

Table S37: Cartesian coordinates of optimized geometry **7b** (*R*)-(−*gauche*).

Compound: 7b (*R*)-(−*gauche*)					
Number of Imaginary Frequencies: 0					
Sum of Electronic and Thermal Free Energies = −783.607571 Hartree					
Center Number	Atomic Number	Atomic Type	Coordinates (Angstroms) X	Y	Z
1	6	0	1.670800	−1.757329	0.296032
2	1	0	1.728967	−2.651489	0.921391
3	1	0	1.564515	−2.074092	−0.753480
4	6	0	2.933709	−0.907031	0.447930
5	1	0	3.062894	−0.649662	1.510994
6	6	0	2.770548	0.386289	−0.344457
7	1	0	2.702624	0.130111	−1.414034
8	6	0	1.486968	1.108459	0.053845
9	1	0	1.575992	1.435389	1.102949
10	6	0	0.286574	0.151047	−0.057463
11	1	0	0.165147	−0.130510	−1.112692
12	8	0	0.522173	−1.029244	0.727215
13	7	0	−0.897478	0.816825	0.383656
14	8	0	1.345299	2.223429	−0.815893
15	1	0	0.476544	2.617952	−0.625700
16	8	0	3.930766	1.178365	−0.105325
17	1	0	3.846459	1.977869	−0.650533
18	8	0	4.043223	−1.660398	−0.023274
19	1	0	4.785255	−1.034324	−0.079928
20	6	0	−4.835667	−0.561757	−0.310962
21	1	0	−5.852905	−0.896996	−0.490653
22	6	0	−4.576302	0.466479	0.600263
23	1	0	−5.394143	0.940520	1.136801
24	6	0	−3.272364	0.898223	0.829335
25	1	0	−3.081933	1.706781	1.531605
26	6	0	−2.190364	0.304697	0.153780
27	6	0	−2.451496	−0.737297	−0.751170
28	1	0	−1.638993	−1.238042	−1.265861
29	6	0	−3.764218	−1.155001	−0.980119
30	1	0	−3.942931	−1.963665	−1.684198
31	1	0	−0.795959	1.234493	1.303874

Table S38: Cartesian coordinates of optimized geometry **7b** (*S*)-(−*gauche*).

Compound: 7b (*S*)-(−*gauche*)
Number of Imaginary Frequencies: 0
Sum of Electronic and Thermal Free Energies = −783.605725 Hartree

Center Number	Atomic Number	Atomic Type	Coordinates (Angstroms) X	Y	Z
1	6	0	−1.727612	−1.761704	−0.211200
2	1	0	−1.856034	−2.686517	−0.780038
3	1	0	−1.537132	−2.024545	0.841218
4	6	0	−2.980303	−0.879305	−0.298664
5	1	0	−3.218964	−0.690996	−1.354376
6	6	0	−2.704108	0.456409	0.384049
7	1	0	−2.556790	0.268216	1.460268
8	6	0	−1.435865	1.096077	−0.171359
9	1	0	−1.591718	1.319335	−1.238567
10	6	0	−0.266821	0.108482	−0.065229
11	1	0	−0.105331	−0.161151	0.993822
12	8	0	−0.604702	−1.088762	−0.782850
13	7	0	0.913630	0.679824	−0.643498
14	8	0	−1.094133	2.278760	0.540561
15	1	0	−1.897213	2.827202	0.554434
16	8	0	−3.762882	1.388718	0.192331
17	1	0	−4.570070	0.954702	0.516646
18	8	0	−4.096987	−1.458175	0.376221
19	1	0	−4.493049	−2.123479	−0.208292
20	6	0	4.799355	−0.566447	0.438791
21	1	0	5.802278	−0.882537	0.710587
22	6	0	3.720347	−1.444211	0.561751
23	1	0	3.881132	−2.455044	0.928422
24	6	0	2.428400	−1.049860	0.209814
25	1	0	1.606646	−1.754556	0.275102
26	6	0	2.192787	0.253417	−0.264374
27	6	0	3.282954	1.134706	−0.391629
28	1	0	3.112905	2.144464	−0.759127
29	6	0	4.568369	0.724965	−0.045869
30	1	0	5.394016	1.424443	−0.150054
31	1	0	0.838526	1.682185	−0.772153

Table S39: Cartesian coordinates of optimized geometry **7b** (*R*)-(+*gauche*).

Compound: 7b (*R*)-(+*gauche*)
Number of Imaginary Frequencies: 0
Sum of Electronic and Thermal Free Energies = −783.602692 Hartree

Center Number	Atomic Number	Atomic Type	Coordinates (Angstroms) X	Y	Z
1	6	0	2.225574	−1.543044	−0.734104
2	1	0	2.402897	−2.608266	−0.568166
3	1	0	2.760253	−1.239625	−1.647987
4	6	0	2.736053	−0.718797	0.449369
5	1	0	2.247441	−1.073273	1.370627
6	6	0	2.362134	0.743425	0.236865
7	1	0	2.897520	1.109651	−0.655115
8	6	0	0.864346	0.883616	−0.012148
9	1	0	0.325693	0.597122	0.899104
10	6	0	0.435278	−0.033048	−1.181364
11	1	0	0.957698	0.312789	−2.085332
12	8	0	0.817587	−1.386790	−0.900569
13	7	0	−0.968536	0.000722	−1.495047
14	8	0	0.630431	2.256210	−0.323520
15	1	0	−0.316952	2.431899	−0.208665
16	8	0	2.781754	1.470778	1.387244
17	1	0	2.497594	2.389996	1.248377
18	8	0	4.145429	−0.886836	0.534237
19	1	0	4.456202	−0.225915	1.176105
20	6	0	−4.181719	−0.353411	1.225315
21	1	0	−5.015763	−0.446586	1.914663
22	6	0	−4.300216	0.420370	0.067250
23	1	0	−5.230791	0.938383	−0.150484
24	6	0	−3.228880	0.549066	−0.814205
25	1	0	−3.329529	1.165060	−1.705298
26	6	0	−2.009712	−0.109882	−0.563627
27	6	0	−1.900277	−0.905954	0.592960
28	1	0	−0.983744	−1.454258	0.777649
29	6	0	−2.975037	−1.010491	1.476633
30	1	0	−2.866686	−1.626998	2.365533
31	1	0	−1.188939	0.566973	−2.302490

Table S40: Cartesian coordinates of optimized geometry **7b** (*S*)-(+*gauche*).

Compound: 7b (*S*)-(+*gauche*)					
Number of Imaginary Frequencies: 0					
Sum of Electronic and Thermal Free Energies = −783.602627 Hartree					
Center	Atomic	Atomic	Coordinates (Angstroms)		
Number	Number	Type	X	Y	Z
1	6	0	2.021051	−1.425422	−1.072469
2	1	0	2.080096	−2.514600	−1.136317
3	1	0	2.540848	−0.997193	−1.944147
4	6	0	2.682557	−0.922507	0.212454
5	1	0	2.196431	−1.404156	1.075646
6	6	0	2.485899	0.585877	0.325593
7	1	0	3.020031	1.066937	−0.509846
8	6	0	1.008421	0.946634	0.217349
9	1	0	0.482455	0.556924	1.099036
10	6	0	0.412136	0.329241	−1.067351
11	1	0	0.930491	0.792318	−1.919786
12	8	0	0.635141	−1.088922	−1.097242
13	7	0	−0.975351	0.649848	−1.227765
14	8	0	0.922946	2.366067	0.171279
15	1	0	−0.010546	2.579826	−0.001069
16	8	0	3.056103	0.994209	1.566824
17	1	0	2.978373	1.961377	1.611082
18	8	0	4.063310	−1.257439	0.166268
19	1	0	4.480715	−0.762067	0.891781
20	6	0	−4.279228	−0.689075	1.051119
21	1	0	−5.137692	−1.013542	1.631907
22	6	0	−4.437214	0.203043	−0.014292
23	1	0	−5.424195	0.580503	−0.269275
24	6	0	−3.335980	0.625987	−0.753246
25	1	0	−3.467131	1.334340	−1.568251
26	6	0	−2.042567	0.155131	−0.454243
27	6	0	−1.889724	−0.759125	0.602749
28	1	0	−0.916669	−1.182283	0.816513
29	6	0	−3.000533	−1.161587	1.348246
30	1	0	−2.857311	−1.868704	2.161499
31	1	0	−1.237345	0.880875	−2.176903

Table S41: Calculated, experimental and fitted NMR *J*-coupling constants of **5a** with mean absolute error (MAE) and resulting conformer populations.

Calculated J [Hz]	Conformers (*R*)-(+*gauche*)	(*S*)-(+*gauche*)	Expt. J [Hz]	Fitted J [Hz]
$^1J(^{15}$N,C1)	−9.66	−6.01	10.9	8.9
$^2J(^{15}$N,C2)	−1.60	−1.81	1.3	1.6
$^3J(^{15}$N,C3)	−0.80	0.04	0.0	0.6
$^3J(^{15}$N,C5)	−0.82	−0.18	1.1	0.7
$^2J(^{15}$N,H1)	0.60	−1.67	0.0	0.1
^3J(N**H**,H1)	0.45	14.16	3.4	3.4
Population [%]	79	21	MAE [Hz]	0.6

Table S42: Calculated, experimental and fitted NMR *J*-coupling constants of **5b** with mean absolute error (MAE) and resulting conformer populations.

Calculated J [Hz]	Conformers (*R*)-(−*gauche*)	(*S*)-(−*gauche*)	(*R*)-(+*gauche*)	(*S*)-(+*gauche*)	Expt. J [Hz]	Fitted J [Hz]
$^1J(^{15}$N,C1)	−9.07	−14.61	−15.10	−11.39	13.1	12.0
$^2J(^{15}$N,C2)	−2.03	−1.28	0.30	−1.42	1.3	1.3
$^3J(^{15}$N,C3)	−2.59	−1.58	−1.01	−2.40	2.3	2.3
$^3J(^{15}$N,C5)	−0.71	−2.22	−2.35	−1.01	1.4	1.4
$^2J(^{15}$N,H1)	−1.61	0.78	−0.98	−0.81	0	0.8
$^3J(^{15}$N,H2)	−2.54	−2.10	−1.59	−1.55	2.1	2.0
^3J(N**H**,H1)	13.41	1.31	7.79	8.71	8.5	8.5
Population [%]	33	22	17	28	MAE [Hz]	0.3

Table S43: Calculated, experimental and fitted NMR *J*-coupling constants of **6a** with mean absolute error (MAE) and resulting conformer populations.

Calculated J [Hz]	Conformers (*R*)-(+*gauche*)	(*S*)-(+*gauche*)	(*S*)-(*trans*)	Expt. J [Hz]	Fitted J [Hz]
$^1J(^{15}$N,C1)	−12.10	−9.56	−6.26	11.2	11.5
$^2J(^{15}$N,C2)	−3.49	−4.79	−7.74	3.5	3.8
$^3J(^{15}$N,C3)	−0.34	0.40	−0.04	0.0	0.2
$^3J(^{15}$N,C5)	−0.74	−0.25	−0.55	0.8	0.6
$^2J(^{15}$N,H1)	0.82	−1.02	0.63	0.0	0.4
$^3J(^{15}$N,H2)	−0.66	−1.18	−0.39	0.5	0.8
^3J(N**H**,H1)	2.57	11.64	−0.35	4.6	4.6
Population [%]	78	22	0	MAE [Hz]	0.2

Table S44: Calculated, experimental and fitted NMR *J*-coupling constants of **6b** with mean absolute error (MAE) and resulting conformer populations.

Calculated J [Hz]	Conformers (*R*)-(−*gauche*)	(*R*)-(*trans*)	Expt. J [Hz]	Fitted J [Hz]
$^1J(^{15}$N,C1)	−10.33	−9.04	12.7	10.1
$^2J(^{15}$N,C2)	−0.96	−2.61	0.9	1.3
$^3J(^{15}$N,C3)	−2.38	−1.83	2.2	2.3
$^3J(^{15}$N,C5)	−0.77	−1.21	1.2	0.9
$^2J(^{15}$N,H1)	−0.37	0.45	0.0	0.2
$^3J(^{15}$N,H2)	−0.75	−0.25	1.0	0.7
^3J(N**H**,H1)	11.81	1.42	9.8	9.8
Population [%]	89	19	MAE [Hz]	0.6

Table S45: Calculated, experimental and fitted NMR J-coupling constants of **7b** with mean absolute error (MAE) and resulting conformer populations.

Calculated J [Hz]	Conformers (R)-($-$*gauche*)	(S)-($-$*gauche*)	(R)-($+$*gauche*)	(S)-($+$*gauche*)	Expt. J [Hz]	Fitted J [Hz]
$^1J(^{15}N,C1)$	−9.14	−14.34	−15.05	−11.43	13.1	12.1
$^2J(^{15}N,C2)$	−2.09	−1.39	0.32	−1.46	1.2	1.2
$^3J(^{15}N,C3)$	−2.64	−1.57	−1.00	−2.43	2.3	2.0
$^3J(^{15}N,C5)$	−0.97	−2.71	−2.81	−1.32	1.6	1.9
$^2J(^{15}N,H1)$	−1.55	0.79	−1.04	−0.83	0.0	0.8
$^3J(^{15}N,H2)$	−2.51	−2.12	−1.56	−1.56	2.0	2.0
$^3J(N\mathbf{H},H1)$	13.39	1.03	7.61	8.64	8.6	8.6
Population [%]	37	20	27	16	MAE [Hz]	0.4

6 Crystallographic data

Table S46: The crystallographic data and experimental parameters for compounds **6b** and **8b**

**Compound**	**6b**	**8b**
CCDC deposition number	2404410	2404411
Empirical formula	$C_{12}H_{17}NO_5$	$C_{11}H_{15}NO_4$
Formula weight	255.27	225.24
Temperature, K	180	180
Wavelength, Å^3	1.54178	1.54178
Crystal system	orthorhombic	orthorhombic
Space group	$P2_12_12_1$	$P2_12_12_1$
Unit cell dimensions		
a, Å	6.44150 (10)	6.05670 (10)
b, Å	6.73240 (10)	6.4441 (2)
c, Å	28.1270 (5)	28.6219 (7)
Volume, Å^3	1219.78 (2)	1117.11 (5)
Z	4	4
Density (calculated), mg m^{-3}	1.390	1.339
Absorption coefficient, mm^{-1}	0.913	0.855
$F(000)$	544	480
Crystal size, mm	0.033 x 0.104 x 0.116	0.060 x 0.186 x 0.204
θ range for data collection, °	3.142 to 68.226	3.088 to 70.050
Index ranges	$-7 \leq h \leq 7$, $-7 \leq k \leq 8$, $-33 \leq l \leq 33$	$-7 \leq h \leq 7$, $-7 \leq k \leq 7$, $-34 \leq l \leq 34$
Reflections collected	14156	23689
Independent/used reflections	2234 / 2168 [$I > 2\sigma(I)$]	2122 / 2116 [$I > 2\sigma(I)$]
R_{int}	0.028	0.028
Parameters refined	164	201
Final R indices [I > 2σ(I)], R_1	0.0270	0.0232
wR_2	0.0292	0.0237
Goodness-of-fit on F	1.0714	1.0645
Flack parameter	0.22 (13)	0.12 (11)
Largest diff. peak/hole, eÅ^3	0.17 / -18	0.13 / -0.17

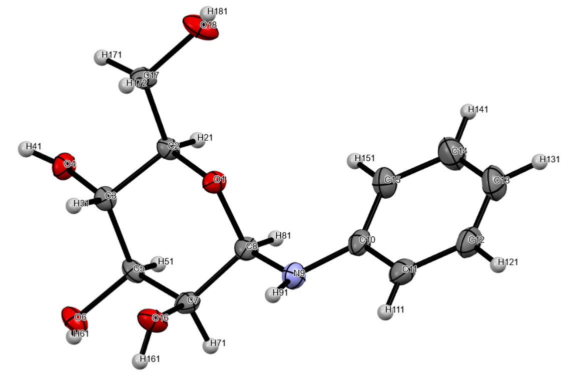

Figure S34: Crystal structure of **6b** showing the (R)-($-$*gauche*) conformer (thermal ellipsoids at 50% probability level).

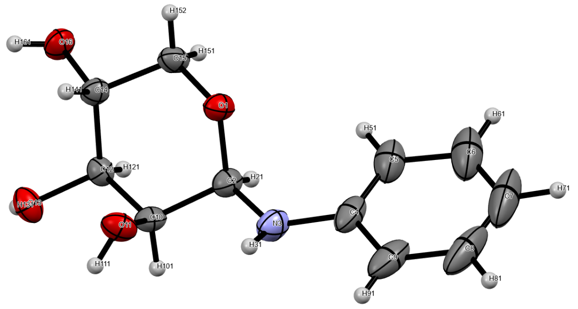

Figure S35: Crystal structure of **8b** showing the (R)-($-$*gauche*) conformer (thermal ellipsoids at 50% probability level).
